# Major Low-Molecular-Weight Metabolites from Freshwater Aquatic Macrophytes: Ecological Aspects

**DOI:** 10.3390/molecules31050895

**Published:** 2026-03-08

**Authors:** Evgeny A. Kurashov, Julia V. Krylova, Alexandra M. Chernova, Yulia V. Bataeva, Eugeny A. Belyakov, Alexander G. Lapirov, Vlada V. Anikina, Viktor A. Grebennikov, Elizaveta Ya. Yavid

**Affiliations:** 1 Papanin Institute for Biology of Inland Waters, Russian Academy of Sciences, Borok 152742, Russia; juliakrylova@mail.ru (J.V.K.); nuphar@mail.ru (A.M.C.); eugenybeliakov@yandex.ru (E.A.B.); a_lapir@ibiw.ru (A.G.L.); 2 Yigal Allon Kinneret Limnological Institute, Israel Oceanographic & Limnological Research Ltd., Migdal 14950, Israel; 3 Russian State Agrarian University—Moscow Timiryazev Agricultural Academy, Moscow 127422, Russia; aveatab@mail.ru; 4 Saint-Petersburg Branch of the Federal State Budgetary Scientific Institution “All-Russian Research Institute of Fisheries and Oceanography” (“GosNIORCH” by L.S. Berg), Saint-Petersburg 196105, Russia; vapity94@mail.ru (V.V.A.); vpauk@mail.ru (V.A.G.); 5 LLC “GES”, Moscow 117393, Russia; eyavid@mail.ru

**Keywords:** freshwater macrophytes, low-molecular-weight metabolome, major low-molecular-weight organic compounds, GC–MS, allelopathy, fatty acids, periphyton, phytoplankton, similarity indices (Jaccard, Sørensen–Czekanowski, Morisita–Horn)

## Abstract

Freshwater macrophytes shape not only the morphological “architecture” of shallow-water ecosystems but also their chemical milieu via low-molecular-weight organic compounds (LMWOCs) that may regulate phytoplankton, periphyton, and the microbiome within the leaf/shoot diffusive boundary layer and the surrounding water column. In this study, GC–MS (gas chromatography–mass spectrometry) was used to identify major LMWOCs of the low-molecular-weight metabolome (LMWM) in 11 widely distributed macrophyte species (*Myriophyllum spicatum* L., *Sparganium emersum* Rehm., *Sparganium gramineum* Georgi, the hybrid *Sparganium* × *foliosum* A. A. Bobrov, Volkova, Mochalova et Chemeris, *Persicaria amphibia* (L.) Delarbre, *Potamogeton perfoliatus* L., *Nuphar lutea* (L.) Sibth. & Sm., *Potamogeton pectinatus* L., *Potamogeton natans* L., *Lobelia dortmanna* L., and *Ceratophyllum demersum* L.). Compounds contributing more than 1% to the total LMWOCs pool were considered major, increasing the ecological realism of interpretations by focusing on metabolites more likely to reach effective concentrations in the plant microenvironment. For interspecific comparisons, the maximum recorded values of relative abundance and concentrations were used to estimate species “potential”. In total, 137 major LMWOCs were detected (four remained unidentified), and their numbers varied markedly among taxa (from 11 in *N. lutea* to 71 in *P. perfoliatus*). Similarity analyses (Jaccard, Sørensen–Czekanowski, Morisita–Horn) indicated that similarity based on compound lists and similarity based on dominance structure may diverge, reflecting differences between the “LMWOCs set” and the quantitative architecture of LMWOCs within the LMWM. Fatty acids formed the core of the major fraction in all species: they were among the top three compounds in all 11 macrophytes and ranked first or second in 10 of 11, highlighting the lipid module as a universal “structure–signaling–defense/allelopathy” hub in aquatic plants. Also, an analysis of the ecological-biochemical role of the main major LMWOCs in the studied aquatic macrophytes is presented. Overall, the data offer a comparable, ecologically oriented framework for interpreting chemical regulation of communities in macrophyte-dominated habitats and for selecting target compounds/species for subsequent bioassay and field studies.

## 1. Introduction

Freshwater aquatic macrophytes are among the key habitat-forming components of aquatic ecosystems, shaping the spatial structure and functioning of shallow waterbodies and the littoral zones of large lakes. In ponds, oxbows and slowly flowing river reaches, canals, wetlands, and shallow lake areas (including the littoral), macrophyte stands create most of the structural (three-dimensional) complexity of habitats. By affecting hydrodynamics, the underwater light climate, suspended-matter retention, and the availability of surfaces for colonization, they ultimately determine the distribution and productivity of aquatic biota [1,2,3]. These effects are regarded as one of the central mechanisms maintaining functional heterogeneity in shallow systems, enhancing resistance to eutrophication, and stabilizing a “clear-water” ecosystem state by competing with phytoplankton for resources [3,4,5].

Alongside morphological habitat engineering, macrophytes also structure freshwater systems biochemically, influencing both the water column and the near-bottom layer. Of particular ecological interest are low-molecular-weight organic compounds (LMWOCs), such as phenolics, terpenoids, fatty acids, and other metabolites. These LMWOCs can alter the growth, physiology, and community structure of phytoplankton, epiphyton/periphyton, and the associated microbiome. This chemical regulation involves the synthesis and release of compounds that may act as defensive metabolites, antimicrobial agents, infochemicals, and allelochemicals affecting competing primary producers and associated biota [6,7,8]. In the literature, such regulation is commonly framed as a combination of direct allelopathic effects and indirect pathways. The latter include changes in the resource environment, shifts in selective pressures on taxa, and modifications of trophic and microbial interactions. The contribution of macrophytes to the stability of the “clear-water” state in shallow lakes is often interpreted as the outcome of several simultaneous mechanisms, among which allelopathy is considered biogeochemically significant [7,9,10]. Moreover, it has been reported that allelopathy caused stronger inhibition than the shading effect and nutrient competition and dominated the combined mechanisms [11].

If even a small fraction of the thousands of chemicals produced by different plant species (aquatic and terrestrial) affects their neighbors, then species-specific interactions, natural selection, coevolution, and community functioning may deviate substantially from predictions of conceptual models based solely on resource-competition theory [12]. For submerged macrophytes, the release of compounds with pronounced algicidal activity has been documented (including hydrolysable polyphenols in *Myriophyllum spicatum*), and this has been linked to the suppression of specific phytoplankton groups and/or shifts in competitive outcomes in the water column [13]. Experimental studies also show that macrophyte chemical effects may target not only phytoplankton [14] but also epiphytic communities, reducing fouling on leaf and shoot surfaces and thereby influencing light availability and the photosynthetic performance of the plants themselves [15,16]. Inputs of LMWOCs may additionally occur via autolysis/leaching from submerged macrophyte tissues [17], underscoring the role of macrophyte stands as a continuous source of organic “fuel” and chemical signals (including allelopathic impact) for microbial and algal communities.

A special role in these biochemical interactions is played by the diffusive (boundary) layer at the surface of leaves and shoots, where limited exchange with bulk water generates the steepest microgradients of oxygen, pH, and dissolved substances. The existence of diffusive boundary layers (DBL)—and their dependence on flow velocity, irradiance, and surface roughness—defines the spatial scale of the chemical microenvironment in which direct contacts occur among macrophytes and epiphyton/periphyton, the microbiome, and phytoplankton. Hydrodynamics and biofouling structure can alter oxygen profiles and processes at the leaf–biofilm interface, thereby modifying conditions for microorganisms and epiphytic algae, affecting macrophyte growth, and potentially changing the effectiveness of chemical regulation mediated by macrophyte LMWOCs [8,18,19,20]. Therefore, the ecological “effect” of macrophyte metabolites should be considered not only as a function of their presence in the water but also as the outcome of transport and transformation within the boundary-layer microenvironment of leaves and shoots.

Recent microbiome research further indicates that aquatic plants and their associated microbial communities function as an integrated system in which microbial metabolism can modify the plant’s chemical profile (and thus its metabolites) and, in turn, influence ecological interactions [21]. Particularly important is the concept of the aquatic-plant exometabolome—the set of LMWOCs that actually enter the external environment, forming an external LMWOCs pool and participating in interorganism interactions. For submerged plants, diverse exudate components have been described, and both the composition and intensity of exometabolite release depend on plant species, growth stage, and environmental factors (light, temperature, trophic status, etc.) [22]. Importantly, the exometabolome is not chemically “static”: its composition and activity are determined not only by plant biosynthesis but also by subsequent transformation in the water and within biofilms.

Accordingly, the ecological effect of the LMWOCs is governed by the dynamics of at least three processes: (i) release/input into the DBL and bulk water, (ii) physicochemical partitioning and transformation across microgradients, and (iii) biotic transformation by the microbiome [18,19,23,24].

In metabolomics, the metabolome is viewed as the ensemble of small molecules reflecting genetic and environmental regulation, and plant LMWOCs profiles obtained by mass-spectrometric approaches are characterized by broad chemical diversity [25]. Contemporary metabolomics emphasizes that the metabolome—including the low-molecular-weight metabolome (LMWM)—represents an integrated response of an organism to genotype and environment, while analytical platforms (including gas chromatography–mass spectrometry (GC–MS)) enable the detection of numerous metabolites and the comparison of profiles across organisms and habitats [25,26]. Studies in aquatic metabolomics indicate that plant LMWMs comprise tens, hundreds, and even thousands of LMWOCs from different functional chemical classes [27,28,29,30,31], and GC–MS profiling allows large metabolite sets to be identified and compared, capturing genetic and environmental differences [32,33]. For aquatic macrophytes inhabiting highly heterogeneous environments (gradients of light, flow, mineralization, trophic state, and fouling), such chemical multicomponent complexity provides a basis for fine-scale tuning of plant interactions with aquatic communities.

Clearly, it is impossible for a single study to elucidate the ecological and biochemical functions of the entire set of compounds in macrophyte LMWMs in aquatic ecosystems. Nevertheless, from an ecological perspective, the most likely “drivers” of community-level impacts are the major components of the LMWM, because they account for the largest share of the total LMWOCs pool and thus have the greatest chance to reach effective concentrations in the microenvironment at the plant surface as well as in the water. In the present study, “major components” are defined as compounds contributing more than 1% of the total sum of LMWOCs concentrations, calculated from peak areas in the total ion chromatogram (TIC) [34,35,36]. This focus is methodologically justified for two reasons. First, major components are the most likely to generate measurable organic fluxes and to reach concentrations comparable to biological response thresholds within the boundary layer and the water column [18,19,37]. Second, for many allelopathic systems, a key challenge is the “gap” between effective doses observed in laboratory experiments and real concentrations in nature [12,38,39]. Therefore, prioritizing major compounds increases the ecological realism of interpretations and facilitates translation from chemical profiles to community-level effects.

At the same time, when focusing only on major components, it is essential to recognize that minor metabolites can be highly bioactive at low concentrations [40,41,42]. “Majority” increases the likelihood of ecologically relevant concentrations, but it does not guarantee maximal biological activity. Thus, detailed, targeted studies of the ecological role of particular plant species in aquatic communities should also consider highly bioactive minor components of the LMWM.

Despite the growing number of studies addressing individual allelochemicals in individual macrophyte taxa, directly comparable interspecific datasets on the major fraction of the LMWM, generated within a single study and interpreted in an ecological context, remain limited. Therefore, to our knowledge, the present work provides the first systematic comparison of major LMWOCs across 11 widespread freshwater macrophyte species, integrating metabolomic analysis with an ecological–functional approach and establishing a necessary basis for prioritizing target compounds and macrophyte species for subsequent research in ecological metabolomics.

Given the above, the aims of this work were (i) to identify the major components of the LMWM in widely distributed freshwater macrophytes (*Myriophyllum spicatum* L., *Sparganium emersum* Rehm., *Sparganium gramineum* Georgi, the hybrid *Sparganium* × *foliosum* A. A. Bobrov, Volkova, Mochalova et Chemeris, *Persicaria amphibia* (L.) Delarbre, *Potamogeton perfoliatus* L., *Nuphar lutea* (L.) Sibth. & Sm., *Potamogeton pectinatus* L., *Potamogeton natans* L., *Lobelia dortmanna* L., *Ceratophyllum demersum* L.); and (ii) to analyze their ecological and biochemical roles in aquatic ecosystems, with particular emphasis on potential allelopathic interactions of macrophytes with phytoplankton and periphyton (epiphytes). All of the listed species typically form well-developed plant associations in characteristic habitats and thus provide a representative model for discussing how major macrophyte metabolites may participate in the chemical regulation of community structure and productivity in shallow freshwater ecosystems.

## 2. Results and Discussion

### 2.1. General Characterization of the Major-Component Composition of the Studied Species

In total, 137 LMWOCs were identified as major compounds across the 11 studied species, because they occurred at concentrations >1% of the sum of all LMWOCs. Four of these major components remained unidentified. The number of major LMWOCs varied widely among the studied taxa, ranging from 11 compounds in *N. lutea* to 71 LMWOCs in *P. perfoliatus* (Table 1).

Only three compounds were major components in all 11 macrophyte species, namely the fatty acids tetradecanoic acid (myristic acid) and hexadecanoic acid (palmitic acid), and the alkane tricosane (Supplementary Materials, Table S1). Two additional LMWOCs were major components in 10 out of 11 species: (E,7R,11R)-3,7,11,15-tetramethylhexadec-2-en-1-ol (phytol) and (9Z,12Z)-octadeca-9,12-dienoic acid (linoleic acid) (Supplementary Materials, Table S1). When considering the distribution of major components among chemical classes in the studied species, the following characteristic patterns can be highlighted.

#### 2.1.1. Species of the Genus *Sparganium*

The distribution of major components in the studied parental species *S. gramineum* and *S. emersum*, as well as in their hybrid *S.* × *foliosum*, is presented in Table 1. The highest number of major LMWOCs (25) was found in the *S.* × *foliosum* hybrid, and the lowest (13) in *S. emersum* (Table 1). For *S. gramineum*, 20 major LMWOCs were noted.

Both the parental species and the hybrid showed a predominance of hydrocarbons among the major components of the LMWM (Table 1). Subdominant groups differed: in *S. emersum* and *S.* × *foliosum*, the largest contributions after hydrocarbons were made by alcohols and carboxylic acids, whereas in *S. gramineum* they were carboxylic acids and aldehydes (Table 1).

Overall, the analysis of the LMWM of *Sparganium* taxa revealed pronounced interspecific differences both in the relative distribution of major LMWOCs across chemical groups and in their number and absolute abundance (Table 1).

For *S. emersum*, characterized by a broad ecological amplitude, the most conservative elements of LMWM organization were the dominant fractions (primarily hydrocarbons and alcohols), as reflected by the relatively low variability of their shares. At the same time, the species’ ecological plasticity appeared to be realized mainly through “plastic modules”—carboxylic acids and, to a lesser extent, ketones—for which increased variability was observed (Table 1). Similarly, the absolute content of major LMWOCs in this species exhibited lower variability than in the other two taxa (Table 1), indicating a tightly regulated, conservative metabolome organization. Such a combination of a stable metabolomic core with variable functional groups may be regarded as one mechanism supporting ecological resilience in aquatic plants with high tolerance to changing environmental conditions [43,44,45].

In *S. gramineum* (an oligosaprobic species), the LMWM was characterized by the maintenance of a conservative core due to the dominant hydrocarbon fraction (low variability), alongside distinct within-group differentiation of the remaining components. At the same time, acids, aldehydes, alcohols, and especially sulfur-containing compounds displayed high to extremely high variability (Table 1), pointing to their regulatory and ecologically adaptive roles. Consequently, the metabolomic plasticity of this species may be associated with dynamics of specific functional groups while maintaining an overall metabolic balance. The observed variability patterns of major LMWM components may indicate a greater sensitivity of its metabolism to environmental conditions (the trophic status of the waterbody). Elevated metabolic sensitivity of specialized taxa to environmental change has been reported in studies of plant metabolomic regulation [46,47].

The hybrid *S.* × *foliosum*, inhabiting a wide trophic range (from oligotrophic to eutrophic lakes), occupied an intermediate yet non-additive position relative to the parental forms. The total number of major components in the hybrid was markedly higher than in the parental taxa, and they were distributed across six chemical groups, whereas in the parental forms only five (Table 1). Thus, the LMWM of *S.* × *foliosum* combines a stable structural core with pronounced hypervariability of individual functional groups (sulfur-containing compounds, ketones, and carboxylic acids) (Table 1), indicating non-additive, hierarchically more complex regulation of metabolism in the hybrid.

It has been shown [48] that hybridization often leads to niche expansion via non-additive phenotypic and physiological effects. This agrees with our results on the variability of major LMWM components, which reflect the hybrid’s capacity to occur across the full trophic spectrum of lakes (from oligotrophic to eutrophic types) combined with a high quantitative level of metabolism. It has also been demonstrated that increased metabolite accumulation in hybrids is linked to non-additive regulation of metabolic pathways [47]. The results obtained allow us to hypothesize qualitatively more complex regulation of individual metabolite groups in *S.* × *foliosum*, which is supported by findings from [49] showing that hybrids are characterized by non-additive and “reconfigured” metabolic pathways. In addition, hybrids may exhibit increased variability of specific metabolic modules while maintaining moderate stability of the overall metabolome [50], consistent with the combination of stable structure and high variability of particular groups in *S.* × *foliosum*. The extremely high variability of sulfur-containing compounds and ketones in *S.* × *foliosum* is further supported by documented transgressive (exceeding the parental range) metabolite variation in hybrids [51].

From a broader evolutionary perspective, the metabolomic patterns observed in the parental taxa *S. gramineum* and *S. emersum* and their hybrid *S.* × *foliosum* may reflect both phylogenetic constraints and adaptive/ecological modulation. The presence of a stable ‘structural core’ in *S.* × *foliosum* is consistent with a phylogenetic signal, reflecting shared biosynthetic capacities inherited from the parental species. In contrast, the non-additive and transgressive variability of specific functional groups suggests a hybrid-specific regulatory reconfiguration, which may increase ecological plasticity, for example, along trophic gradients. Notably, disentangling phylogenetic signal from habitat-driven effects would require dedicated phylogenetic comparative studies and broader sampling, which is beyond the scope of the present study.

Thus, the composition of major LMWOCs in the hybrid *S.* × *foliosum* supports an ecological interpretation of this hybrid as a form with enhanced adaptive capacity, reflecting the general tendency toward the wide occurrence of hybrid *Sparganium* forms in diverse aquatic habitats and their ecological significance [44,45].

#### 2.1.2. Species of the Genus *Potamogeton*

The composition of major LMWOCs in the LMWM was most diverse in the *Potamogeton* species (Table 1; Supplementary Materials, Table S1). The total number of identified major LMWOCs was similar (36 and 31) in *P. pectinatus* and *P. natans*, whereas it was maximal (71) in *P. perfoliatus* among all studied macrophyte species.

The number of major LMWOCs per sample (*n*) in the *Potamogeton* species exhibited low variability (from CV = 0.11 in *P. natans* to 0.19 in *P. perfoliatus*) (Table 1). The relative contribution of major LMWOCs to the total LMWOCs pool also showed very low to low variability (CV = 0.05–0.12) across all three species, whereas the absolute concentrations of major LMWOCs varied strongly (CV = 0.56–1.04) (Table 1).

According to the obtained data (Table 1), the LMWM of *P. pectinatus* is characterized by: (i) the dominance of primary-metabolism classes (carboxylic acids, alcohols, ketones), (ii) a high share of oxygen-containing classes among major compounds, and (iii) a clear separation between a conservative “metabolic core” (carboxylic acids, alcohols, ketones)—where alcohols and ketones show the lowest variability—and an ecologically plastic periphery, i.e., groups with very high variability (phenols, aldehydes, and diverse functional groups).

The LMWM of *P. natans* (Table 1) fundamentally differs from that of *P. pectinatus* and is shifted from an acid-oriented to a lipid/ester-oriented metabolome type. This is supported by: (i) a high proportion of esters and alcohols, indicating enhanced lipophilic surface fractions (including wax esters and long-chain alcohols) that shape barrier and interfacial properties of the leaf surface [52,53], and also pointing to active chemical interactions with the environment [10,54]; (ii) a broader spectrum of compounds classified as diverse functional groups, which may be typical for floating-leaved species [55,56] inhabiting conditions of variable light and hydrodynamics [57]; and (iii) high variability of carboxylic acids, reflecting dependence on environmental conditions (light, trophic state, CO_2_, etc.) [58,59]. In contrast to *P. pectinatus*, which exhibits a conservative, “primary” metabolome, *P. natans* demonstrates a metabolomic strategy of ecological plasticity close to an adaptive/signaling type [7,54,60].

The LMWM of *P. perfoliatus* (Table 1) appears to reflect a moderately plastic, but not extreme, strategy that is intermediate between the conservative type (*P. pectinatus*) and the ecological/signaling type (*P. natans*). This is suggested by key features such as: the absence of a clearly dominant chemical group; an increased contribution of oxidative and stress-associated fractions (aldehydes, ketones); a minimal contribution of phenolic and aromatic compounds; and overall very high variability of absolute concentrations, indicating sensitivity to environmental conditions. As a typical submerged macrophyte with a well-developed leaf apparatus, *P. perfoliatus* exhibits a LMWM oriented toward balancing primary metabolism and ecological adaptation, including allelopathic regulation of phytoplankton development [10,54].

A comparison of the metabolomic profiles of the three *Potamogeton* species demonstrates a clear link between the chemical organization of the LMWM, the morpho-ecological growth form, and the potential role of macrophytes in allelopathic interactions with phytoplankton and epiphytes. These results are consistent with the concept of species specificity and the context dependence of allelopathy in aquatic plants [60].

The floating-leaved species *P. natans* is characterized by a LMWM with an increased proportion of lipophilic and ester compounds, which is consistent with reports on specialized antialgal furanoditerpenes in this species capable of suppressing the growth of microalgae [56]. Experimental studies also show that floating-leaved macrophytes can, in some cases, inhibit cyanobacteria more effectively than fully submerged forms, supporting the interpretation of *P. natans* as a chemically active regulator of their abundance [61].

The fully submerged species *P. pectinatus* and *P. perfoliatus* demonstrate more conservative metabolomic profiles. Nevertheless, submerged macrophytes are also known to exert allelopathic inhibitory effects on phytoplankton [54], and the antialgal activity of their exudates is often associated with lipophilic fractions [62]. For *P. perfoliatus*, interactions with epiphytes are particularly important: epiphytic overgrowth substantially affects the photobiological characteristics and plant production, making chemical regulation of epiphytes functionally significant [63,64].

#### 2.1.3. *Myriophyllum spicatum*

A total of 25 compounds were identified in the major LMWOCs fraction of *M. spicatum*, with carboxylic acids and hydrocarbons dominating both in terms of cumulative relative contribution and absolute concentration (Table 1; Supplementary Materials, Table S1). The mean cumulative share of the accounted groups was high and exhibited very low variability (82.01% of total LMWOCs, CV = 0.04). In contrast, the total concentration of major LMWOCs was characterized by very high variability (CV = 0.69). On average, 17 major LMWOCs were identified per sample (CV = 0.23), indicating the presence of a relatively stable qualitative “core” of the major fraction despite pronounced variability in the contributions of individual chemical classes.

The major LMWOCs of the LMWM of *M. spicatum* are characterized by a marked lipid–hydrophobic dominance, with carboxylic acids and hydrocarbons playing a key role. The predominance of carboxylic acids, both in relative contribution and concentration, indicates a substantial role of the fatty acid pool in shaping the chemical influence of *M. spicatum* on the surrounding aquatic environment. For *M. spicatum*, it has been demonstrated that released fatty acids exhibit anti-cyanobacterial activity and may directly contribute to allelopathic effects in aquatic ecosystems [65,66]. The prominent variability in fatty acid concentrations, combined with more moderate variability in their relative contribution, reflects the dual role of lipids as structural components and as participants in dynamic physiological processes within the plant itself, as well as in biochemical interactions within the aquatic ecosystem (e.g., direct chemical effects on phytoplankton and microbiota).

The substantial contribution of hydrocarbons highlights the importance of hydrophobic components in the major fraction. The high proportion of alkanes and their low variability, together with the limited variability in the number of alkanes among samples, may be related both to relatively conservative biosynthetic control of these compounds and to their sorption and biotransformation on plant surfaces and by epiphytic biota [52].

Alcohols form a relatively stable supporting class, whereas aldehydes and ketones exhibit increased variability, which is consistent with their role as products of oxylipin and oxidative pathways activated under damage or stress conditions [67,68]. These classes represent the “reactive” part of the LMWM, which is sensitive to environmental conditions and largely responsible for plant adaptation to specific habitats.

Overall, the profile of major components in *M. spicatum* indicates a functional differentiation between a stable lipid–hydrocarbon “framework” and highly variable regulatory compounds. This metabolomic pattern is in good agreement with the well-documented high allelopathic activity of the species, where the overall effect is formed by a combination of, for example, polyphenolic and lipid–acid components [13,40,65,66,69].

#### 2.1.4. *Persicaria amphibia*

Across the groups of major LMWOCs, *P. amphibia* exhibited a fatty-acid-dominated structure (Table 1), as carboxylic acids made the largest contribution to the total pool of major components.

The predominance of carboxylic acids (≈53% of the sum of major LMWOCs) and their low relative variability (CV = 0.10) indicate that, in *P. amphibia*, the lipid fraction forms a “conservative” metabolic framework. Their mean number per sample (6) was also high, with low variability of this metric (CV = 0.20). At the same time, the variability of the absolute content of fatty acids in the LMWM was high (CV = 0.55) (Table 1), suggesting that the level of their biosynthesis changes depending on specific aquatic environmental conditions and pointing to a strong regulatory role.

Fatty acids and their derivatives simultaneously perform structural (membranes/cuticular lipids), energetic, and protective functions; many free fatty acids participate in allelopathic interactions, exhibit antimicrobial activity, and may affect the plant-surface microbiome and periphyton [70,71]. In addition, unsaturated fatty acids are precursors of oxylipins—a central stress-signaling cascade associated with plant damage, herbivores, and pathogens [72].

Substantially smaller shares were observed for aldehydes and ketones, with different levels of variability in their relative representation and in the number of compounds in the LMWM—low for aldehydes and very high for ketones (Table 1). High variability was also characteristic of their absolute contents, indicating a close linkage between their synthesis and ambient environmental conditions. Similarly high variability in contents and concentrations was found for alcohols and hydrocarbons (Table 1).

The remaining groups (aromatic hydrocarbons, esters, sulfur-containing compounds, and unidentified components) formed a set of minor constituents characterized by a high level of variability across all metrics. Overall, major LMWOCs accounted on average for 72.71 ± 2.78% of the total LMWOCs pool, with a very low level of variability (CV = 0.05); on average, 15 ± 1 major compounds were recorded per sample, also with very low variability (CV = 0.07), out of a total pool of N = 21.

The moderate shares of aldehydes and alcohols (together ~9%) are consistent with their role as components of “green leaf volatiles” (GLVs), which are rapidly produced upon damage and are involved in direct and indirect defense (signaling, antimicrobial effects, and mediation of trophic interactions) [73].

The high variability of ketones and hydrocarbons (CV for proportion = 0.62–0.65) may reflect the plasticity of surface lipids/waxes, which are known to constitute a dynamic barrier responding to abiotic and biotic factors; waxes include alkanes, aldehydes, ketones, and esters and influence stress tolerance and surface colonization [74,75]. For the amphibious species *P. amphibia*, with contrasting aquatic and terrestrial forms, such plasticity is particularly expected and is consistent with the high ecological variability of *P. amphibia* [76]. Finally, the broader context of the chemical “landscape” of leaf surfaces supports the interpretation of minor wax-related groups as modules of the plant–environment interface, where small fractions may exert disproportionately important effects [77]. Although such effects have been documented mainly for terrestrial plants, similar effects can be expected in aquatic macrophytes, especially in amphibious taxa and species with floating leaves.

#### 2.1.5. *Nuphar lutea*

In this study, we analyzed the LMWM of yellow water lily (*N. lutea*) inhabiting a single lake on the Karelian Isthmus (Northwest Russia) whose trophic status changed among years [78].

The LMWM of *N. lutea* was characterized by an extremely strong dominance of carboxylic acids, which accounted on average for 78.67 ± 8.28% of the total pool of major LMWOCs (Table 1). Despite low variation in relative abundance (CV = 0.11), the absolute concentrations of carboxylic acids exhibited very high variability (3388.91 ± 2850.72 μg·g^−1^ DW; CV = 0.84), indicating substantial fluctuations in the level of their metabolic synthesis, most likely associated with changing environmental conditions in this lake across different years [78].

The second most important group comprised hydrocarbons, contributing 4.32 ± 1.41% with a relatively high CV (0.33) and moderate absolute concentrations (202.64 ± 103.14 μg·g^−1^ DW; CV = 0.51). Aldehydes and esters occurred at minor proportions and were characterized by high variability in both relative abundance and concentrations (Table 1). Alcohols were detected only as a single major component (*n* = 1) and exhibited extremely high variation in concentration (CV = 1.41), highlighting their episodic role within the major LMWOCs pool of this species.

Overall, major LMWOCs constituted most of *N. lutea* LMWOCs pool (87.32 ± 1.77%) with very low variability (CV = 0.02), while the number of major compounds remained consistently low (9 ± 1; CV = 0.16).

The identified LMWM structure of *N. lutea* reflects a highly specialized strategy dominated by a limited number of high-concentration compounds, namely fatty acids, which act as precursors of oxylipins/signaling and defense-related molecules and may serve as potential inhibitors of cyanobacteria and other microorganisms [71,72,79].

This pattern is consistent with the notion that the lipid/fatty-acid block in yellow water lily serves both as a “metabolic framework” (membranes, energy metabolism) and as a source of ecologically relevant derivatives capable of affecting epiphyton and phytoplankton [7,60].

This organization of the major pool differs fundamentally from the more “diversified” profiles obtained for *M. spicatum* and *P. amphibia*. A key feature of *N. lutea* is the discrepancy between the stability of relative composition and the variability of absolute concentrations. This agrees with evidence that, in aquatic macrophytes, the contributions of functional groups and the strength of allelopathy/chemical control of biota are highly species-specific. At the same time, the intensity of allelochemical release/flux and their actual effectiveness in a waterbody are strongly regulated by environmental conditions, microbial degradation, and dilution effects [10,15,80].

The low and stable number of major compounds in *N. lutea* emphasizes that its defensive/allelopathic potential is more likely realized through quantitative amplification of a limited set of metabolites rather than through chemical diversity. From an ecological perspective, this strategy may enable flexible regulation of chemical pressure on microbial, epiphytic, and phytoplankton communities while maintaining a stable set of “functional” molecules. It has been shown that combinations of polyphenols and fatty acids can suppress cyanobacteria; even if the specific molecules differ, the underlying mechanism—locally high effective concentrations and additive/synergistic effects of allelochemical mixtures—appears to be common across allelopathic systems of aquatic plants [66,81].

#### 2.1.6. *Ceratophyllum demersum* and *Lobelia dortmanna*

In both species, “major” LMWOCs constituted the main fraction of the LMWM. However, the structure of the metabolite pools differed markedly.

*C. demersum.*

Major groups accounted for 70.97% of the total LMWOCs pool, with a cumulative concentration of 39.67 μg·g^−1^ DW and 26 major compounds (*n* = 26). The largest contribution was made by ketones, aldehydes, and carboxylic acids, which together comprised more than 50% of the LMWM. Esters, hydrocarbons, and alcohols contributed substantially less to the LMWM of *C. demersum* (Table 1). Overall, the metabolomic profile of *C. demersum* can be characterized as “multiaxial”, with several co-dominant chemical groups.

*L. dortmanna*.

In this species, major groups comprised 78.57% of the total LMWOCs pool, with a substantially higher cumulative concentration (156.16 μg·g^−1^ DW) but a lower number of major compounds (*n* = 13). The LMWM was strongly dominated by carboxylic acids and hydrocarbons, whereas ketones were of secondary importance. Aldehydes and alcohols were present only at trace levels (~1% each; *n* = 1), and esters were not detected among the major LMWOCs (Table 1).

Despite the lack of estimates of spatial variability, the major LMWOCs profiles clearly reflect different “dominance strategies” in these two species. In *C. demersum*, the distribution of the major pool among ketones, aldehydes, and carboxylic acids is typical of active lipid metabolism and oxylipin-related pathways. Free fatty acids serve as substrates for the formation of stress-induced aldehydes and alcohols, as well as other lipid oxidation products, which—together with fatty acids—are involved in signaling, defense, and allelopathic processes [66,67,72]. The substantial contribution of such “reactive” compound classes is consistent with the role of *C. demersum* as a chemically active component of shallow-water biocenoses, capable of generating locally high concentrations of biologically active metabolites. For this and other submerged macrophytes, inhibitory effects of exometabolites on phytoplankton and cyanobacteria, as well as the role of allelopathy in maintaining clear-water states, have been demonstrated [7,54].

In *L. dortmanna*, the dominance of carboxylic acids and hydrocarbons (>71% of the pool; 141.82 μg·g^−1^ DW) combined with a low number of major compounds indicates a “structural–barrier” orientation of the LMWM. Long-chain lipids and their derivatives are known to form the basis of hydrophobic plant surface layers, regulating tissue permeability and mechanical stability [53]. This profile is consistent with the physiology of isoetid plants, which are characterized by restricted leaf gas exchange and a substantial contribution of root-mediated uptake of inorganic carbon from the sediment [82,83]. Thus, the more “constructive” lipid–barrier metabolomic profile observed in *L. dortmanna* may represent an adaptive strategy to oligotrophic, soft-water habitats.

### 2.2. Similarity Among Species Based on Their Major LMWOCs

Table 2 and Table 3 present similarity estimates among the studied species based on data on their major LMWOCs, calculated using the Jaccard similarity coefficient (J), the Sørensen–Czekanowski coefficient (Qs), and the Morisita–Horn index (Cmh).

Similarity matrices constructed from major LMWOCs using these three indices (Jaccard, Sørensen–Czekanowski, and Morisita–Horn) revealed stable “blocks” of similarity and demonstrated that similarity in composition (presence/absence) and similarity in the structure of dominant components (abundance-weighted) partly diverge. This pattern is expected for metabolomic data, where a relatively small set of compounds may account for most of the total concentration and thus “bring species closer” according to the Morisita–Horn index, even when the overlap in component lists is only moderate [84].

The most internally consistent cluster is formed by the genus *Sparganium* (*S. emersum, S. gramineum*, and *S.* × *foliosum*). According to the J and Qs metrics, *Sparganium* species exhibit relatively high intragroup similarity, with J = 0.50–0.57 and Qs = 0.67–0.73 between species pairs and the hybrid. Similarity based on the Morisita–Horn index is even higher (Cmh = 0.86–0.96), indicating that not only the set of major LMWOCs but also the dominance structure is highly concordant. Biologically, this is consistent with the notion that closely related taxa often retain similar chemotypes and functional chemical strategies (e.g., defense, antifouling, allelopathy, signaling lipids) [7,85]. The high degree of similarity between the hybrid taxon and its parental species agrees well with evidence that the chemical phenotype of hybrids is often shaped by inheritance and redistribution of parental specialized metabolites, resulting in additive or non-additive, intermediate, or dominant patterns in both compound composition and concentrations [86,87,88,89].

A particularly distinct pair of species is *P. amphibia* and *L. dortmanna*, which exhibit the highest quantitative similarity that cannot be explained by taxonomic relatedness. For this pair, very high similarity according to the Morisita–Horn index (Cmh = 0.90) is observed alongside much more moderate compositional overlap (J = 0.26; Qs = 0.41). This combination implies similarity specifically in dominant molecules and indicates convergence at the level of dominant classes or compounds, i.e., leading major components form a similar “mass” of the metabolomic pool, while a substantial part of the major LMWOCs lists remains different. Ecologically, this is consistent with the hypothesis that different hydrophytes may converge toward similar basic strategies: (i) chemical defense and (ii) effects on microbial and algal assemblages through allelopathy, where major LMWOCs present at high concentrations are most likely to reach biologically effective levels [7,10,65]. In our case, fatty acids—represented by palmitic, linoleic, α-linolenic, and other acids—were the dominant components in these species (Table 1; Supplementary Table S1). These LMWOCs may function both as structural membrane lipids and as biologically active molecules involved in allelopathic, stress-related, and defensive responses [67,71,73].

Closely associated with this species pair is *N. lutea*, which also shows elevated similarity with them, as well as with *M. spicatum*, according to the Morisita–Horn index (Cmh = 0.69–0.81; Table 3). At the same time, similarity based on J and Qs indices is more moderate (Table 2). This pattern is typical when similar dominant metabolic pathways—primarily lipid and fatty-acid pathways and their derivatives (oxylipins and signaling molecules)—shape a comparable “mass” structure of the LMWM in ecologically different plants [72,79,90]. In aquatic macrophytes, fatty acids and their associated products and functions are often considered a key component of chemical defense and interspecific interactions, including effects on phytoplankton and cyanobacteria [7,10,65].

*M. spicatum* exhibits moderate similarity with *Sparganium* species according to Qs (0.44–0.53) and Cmh (0.47–0.58). This is consistent with the presence of well-documented chemical–ecological mechanisms in *M. spicatum*, including allelopathically active polyphenols and a range of fatty acids, which may generate a prominent dominance structure and exert strong effects on phytoplankton, including cyanobacteria [65,66]. At the same time, the compositional “tail” of major compounds may differ among genera, thereby reducing J and Qs values.

Within the genus *Potamogeton*, no single dense cluster of similarity is observed among the studied species, in contrast to what might be expected by analogy with *Sparganium*. Instead, only moderate similarity occurs between individual species pairs. For example, *P. pectinatus* and *P. natans* show Cmh = 0.54 at low J (0.16) and Qs (0.27), indicating relatively high quantitative similarity in dominant components but low similarity in the lists of major LMWOCs. This pattern may reflect the pronounced ecological plasticity of the genus *Potamogeton* (variation in habitat conditions, seasonality, stressors), under which a conserved “core” of dominant compounds or classes is maintained, whereas the set of accompanying major components varies [29,91,92]. In contrast, *P. perfoliatus* generally exhibits lower similarity with most species, indicating a more specific major LMWOCs pool.

In the similarity analysis, *C. demersum* showed predominantly low J and Qs values with all species (Table 2), low Cmh values with most species, and relatively higher similarity only with *P. pectinatus* (Cmh = 0.58; Table 3). This pattern highlights its chemical specificity, possibly related to the absence of a clearly dominant compound group among its major LMWOCs (Table 1). Nevertheless, for *C. demersum*, as for other studied macrophytes, allelopathic activity has been documented in both experimental and field studies [54,93,94,95].

Thus, beyond a purely statistical interpretation, the interspecific similarity matrices (Table 2 and Table 3) can be interpreted in relation to species’ ecological traits. Similarity in the dominance structure of major LMWOCs can be expected among species sharing comparable functional types and habitats, whereas contrasting environmental settings across different waterbody types (e.g., oligotrophic vs. eutrophic systems) may shift metabolite allocation and relative importance, thereby altering dominance patterns in the LMWM. Accordingly, discrepancies between list-based similarity and similarity based on quantitative dominance likely reflect shared eco-biochemical regularities in LMWM formation in aquatic macrophytes, while the specific quantitative architecture of the metabolome is, to a substantial extent, shaped by ecological context.

### 2.3. Ecological-Biochemical Role of the Main Major LMWOCs of the Aquatic Macrophytes

Section 2.1 and Section 2.2 described the overall structure of the major LMWOCs pool and interspecific differences in the composition and chemical classes of major LMWOCs. In this section, we focus on those compounds that constitute the core of the major pool based on relative contribution and examine the ecological and biochemical roles of the most important major LMWOCs (as well as their chemical groups) identified in the studied macrophytes. These compounds are most likely to determine functional effects in aquatic ecosystems within macrophyte-dominated habitats.

Importantly, when discussing the ecological roles of LMWOCs, it is essential to consider the context of the plant–water interface, where a DBL may generate steep concentration gradients and prolong the residence time of released metabolites relative to the bulk water. This microenvironment constitutes a key locus for interactions with epiphytes and the microbiome and may therefore increase the functional relevance of major LMWOCs even when their concentrations in bulk water are low. Accordingly, we interpret dominant classes of major LMWOCs not only in terms of their biochemical origin but also with respect to their potential to operate within DBL-scale microenvironments.

Table 4 presents the top three most abundant major LMWOCs for each studied macrophyte species. Notably, carboxylic acids are included among the top three major LMWOCs in all species, and in 10 out of 11 species, they occupy either the first or second position, or both.

#### 2.3.1. Fatty Acids

The predominance of fatty acids—primarily palmitic, linoleic, and α-linolenic acids—indicates the dominance of a lipid metabolic block within the major LMWOCs pool. This pattern is particularly evident in *N. lutea*, where these three acids together constitute a substantial proportion of the major pool (α-linolenic acid 31.31%, palmitic acid 29.06%, linoleic acid 25.03%). A similar pattern is observed in *P. amphibia* (palmitic acid 42.53%, α-linolenic acid 10.43%, myristic acid 8.33%), *P. pectinatus* (palmitic acid 23.92%, myristic acid 14.55%), and *P. perfoliatus* (α-linolenic acid 18.41%, palmitic acid 13.55%). Functionally, such a “fatty-acid core” is consistent with the well-established role of fatty acids—particularly unsaturated ones—as key precursors of oxylipins involved in stress responses, chemical signaling, antimicrobial and anti-grazer defense, and allelopathic interactions [79,81].

In aquatic macrophytes, fatty acids can be regarded as a functional node linking structure, signaling, and ecological impact, realized in the aquatic environment primarily through bioavailable free fatty acids (FFAs) released during tissue damage, exudation, and detrital decomposition.

From a *Metabolome*→*Defense* perspective, the observed dominance of linoleic acid (C18:2) and α-linolenic acid (C18:3) among the top 3 components (Table 4) is of particular importance. These fatty acids serve as substrates for lipid-mediated signaling and oxylipin/jasmonate-dependent cascades; therefore, the high C18:3/C18:2 maxima observed in *N. lutea* and *P. perfoliatus* may be interpreted as reflecting an increased defensive capacity of the lipid module and readiness for stress-induced responses to epiphytic, microbial, and cyanobacterial pressure [96,97].

The transition from *Defense/Allelopathy*→*Periphyton/Phytoplankton* is most likely to occur during phases when lipids are converted into FFAs. Experimental studies have demonstrated that FFAs exert cytotoxic effects on algae and cyanobacteria primarily via membrane disruption, including increased permeability, ion leakage, and secondary physiological disturbances, with unsaturated fatty acids generally exhibiting stronger effects than saturated ones [98]. At the same time, in an experimental study [99], it was shown that the effect of nonanoic acid against the cyanobacterium *Synechocystis aquatilis* Sauvageau (suppression coefficients were more than 20) was more pronounced than that of palmitoleic acid (suppression coefficients were not more than 3.5). In *M. spicatum*, the release of fatty acids with strong anticyanobacterial activity has been experimentally confirmed [65]. In this context, the high fatty-acid contents observed in this and other studied species support the interpretation of species rich in fatty acids—especially unsaturated ones—as potentially lipid-mediated allelopathically active macrophytes influencing phytoplankton and biofilm formation.

At the periphyton level, fatty acids may act as selective allelochemical agents reshaping the bacterial core of biofilms. The antibacterial activity of FFAs is associated with damage to the cellular membrane, disruption of electron transport and oxidative phosphorylation, impairment of cellular energy production, inhibition of enzyme activity, reduced nutrient uptake, formation of peroxidation and auto-oxidation products, or direct bacterial cell lysis [100,101]. As a result, both the rate and intensity of surface colonization and the functional structure of epiphytic communities may be altered [100]. Importantly, the most realistic ecological scenario involves the action of allelochemical mixtures rather than single compounds, with fatty acids contributing additive or synergistic effects alongside other allelochemicals [102,103,104].

Direct allelopathic effects on algae and cyanobacteria have been demonstrated for α-linolenic, linoleic, palmitoleic, dodecanoic, tetradecanoic, pentadecanoic, hexadecanoic, octadecanoic, cis-6-octadecenoic, cis-9-octadecenoic, heptanoic, octanoic, nonanoic, cis-5,8,11,14,17-eicosapentaenoic, and cis-4,7,10,13,16,19-docosahexaenoic acids [65,98,99,105,106,107,108,109,110,111,112]. Notably, the first seven acids in this list may occur as major components in the LMWM of the macrophytes examined in the present study (Supplementary Materials, Table S1).

At the ecosystem level, chemical pressure exerted by FFA-allelochemicals should be regarded as an important component of an integrated mechanism through which macrophytes can reduce phytoplankton competitiveness and maintain clearer-water states. However, this effect is likely achieved not only through allelopathy but also via alternative mechanisms such as light attenuation, competition for nutrients, and reduced nutrient availability for phytoplankton [9,10].

#### 2.3.2. Alcohols

According to the maximum relative content (in %) (Supplementary Materials, Table S1), the alcohol profile in 11 studied macrophyte species is formed by three “cores”: (i) terpenoid alcohols (including labdane-type diterpenes), (ii) chlorophyll-derived alcohols (primarily phytol), and (iii) aliphatic or “green” alcohols (C6–C8 compounds and long-chain fatty alcohols). These groups are potentially associated both with the plants’ intrinsic chemical defense and with epiphytic microbiota and periphyton within the DBL adjacent to plant surfaces.

Among all major alcohols identified, only two compounds—manool and phytol—ranked among the three most abundant components by maximum relative content, and only in three species: *M. spicatum*, *P. perfoliatus*, and *P. natans* (Table 4).

In aquatic macrophytes, alcohol metabolites play a dual role: (1) as intraplastidial and membrane-associated components and products of metabolic remobilization (phytol), and (2) as hydrophobic, surface-active protective molecules (terpenoid alcohols and long-chain fatty alcohols) localized in the integumentary structures and/or on leaf surfaces [113,114,115,116].

This protective “logic” is particularly evident in *P. perfoliatus* and *P. natans*, where manool (16.44–19.61%) and accompanying labdane-type alcohols dominate. Labdane diterpenoids are well known for their antibacterial activity and may affect bacterial energy metabolism, including inhibition of ATP synthesis and hydrolysis [117].

In aquatic environments, the effective “radius of action” of hydrophobic alcohols is generally confined to the microscale, making the DBL a key factor. Periphyton has been shown to increase the thickness of this boundary layer and to modify pH and O_2_ microprofiles at the leaf surface, while microsensor measurements reveal pronounced gradients of pH, O_2_, and redox potential around submerged leaves [19,118]. These conditions favor the local accumulation and retention of lipophilic terpenoid alcohols (including manool) and phytol, allowing them to act during the early stages of epiphyte and microbial colonization and to influence biofilm structure. The observed species specificity of epiphytic bacterial communities on macrophytes is consistent with the notion that the chemical “background” of plant surfaces acts as a selective factor, as demonstrated for *P. perfoliatus* and *M. spicatum* [119].

Among the major alcohols detected, manool deserves particular attention. Manool (a labdane-type diterpene alcohol) is a hydrophobic specialized plant metabolite that, in aquatic systems, most likely acts within contact interaction zones (macrophyte organ surfaces, epiphytic matrices, and detrital particles), where lipophilic terpenes can accumulate locally and affect biofilms [120]. Importantly for hydrophytes, manool has been identified as a common low-molecular-weight metabolite in *P. natans*, rendering it ecologically relevant for freshwater communities [97]. Available data indicate multi-level biological activity: antibacterial effects have been demonstrated against panels of clinically relevant strains, including mastitis-associated bacteria [117,121]. Manool has also been proposed as a promising agent for the treatment of periodontal infections due to its antibacterial properties [122]. Moreover, the antibacterial efficacy of manool, together with sclareol (a major component in *P. natans*), has been demonstrated by [123]. Phytochemical studies of *P. natans* and *Ruppia maritima* L. further suggest that high concentrations of biologically active ent-labdane diterpenes in these plants can affect other aquatic organisms and may exert anti-algal effects, thereby contributing to the maintenance of balance in aquatic systems [56,124,125]. Overall, in aquatic ecosystems, the ecological and biochemical roles of manool and sclareol appear to be primarily associated with antibacterial and anti-algal effects, particularly within biofilms and periphyton.

Another important compound is phytol, an isoprenoid (diterpene) alcohol that forms the phytyl side chain of chlorophylls *a* and *b* in living plant and algal cells and is therefore directly linked to the turnover of the photosynthetic apparatus in aquatic macrophytes, periphyton, and phytoplankton. In aquatic ecosystems, where phototrophs frequently experience fluctuations in light availability, temperature, and nutrient supply, chlorophyll degradation and phytol release are enhanced during periods of photostress. Recent studies consider the “fate of phytol” as a regulated module connecting chlorophyll catabolism with prenyl metabolism and antioxidant defense [116].

Phytol was the leading alcohol in most of the studied species (up to 16.39% in *M. spicatum* and 13.05% in *S. emersum*) (Supplementary Materials, Table S1), which is biochemically consistent with its origin from chlorophyll and with known pathways of phytol remobilization for tocopherol (vitamin E) synthesis and adaptation to oxidative stress [116]. In addition, phytol exhibits a broad spectrum of antibacterial and antifungal activities, and its mechanism of action has been elucidated, including induction of oxidative cell death in *Pseudomonas aeruginosa* [126,127].

Within the “macrophytes–periphyton–phytoplankton” framework, these findings support a working hypothesis of a local influence of phytol and lipophilic chlorophyll degradation products on the early stages of biofilm formation and on the composition of surface-associated microbial communities. Phytol has also been shown to exert protective or deterrent effects against aquatic insects and herbivorous larvae [128]. Furthermore, higher phytol concentrations combined with the presence of linoleic acid in the leaves of *Nuphar lutea* have been reported to reduce feeding by *Galerucella nymphaeae* L. [129], a typical consumer of *N. lutea* and *Nymphaea candida* J. et C. Presl. [130]. Collectively, these data suggest that phytol should be regarded not merely as a by-product of chlorophyll metabolism, but as an important component of plant chemical defense and resistance to pathogens and microbial colonization of plant surfaces.

In addition to terpenoid and chlorophyll-derived alcohols, long-chain fatty alcohols (primary *n*-alcohols, predominantly C14–C26+)—typical constituents of waxes and surface lipid coatings—were present among major LMWOCs in several species. In the studied macrophytes, these included, for example, tetradecan-1-ol (C14:0) in *M. spicatum* (3.70%); hexadecan-1-ol (C16:0) in *P. perfoliatus*, *P. natans*, and *P. pectinatus* (1.61–4.47%); heptadecan-1-ol (C17:0) in *P. perfoliatus* (4.37%); octadecan-1-ol (C18:0) in *P. perfoliatus* (1.33%); *(Z)*-icos-9-en-1-ol (C20:1) in *S. gramineum* (2.23%); and, among even longer homologues, pentacosan-1-ol (C25:0) and hexacosan-1-ol (C26:0) in *P. perfoliatus* (2.27% and 2.28%, respectively) (Supplementary Materials, Table S1).

The most plausible ecological and biochemical functions of these compounds in aquatic habitats are associated with the formation of a hydrophobic barrier interface, modulation of the immediate microenvironment through changes in surface physicochemical properties that may affect adhesion and early establishment of microbial biofilms and fouling communities, and indirect defense via a “physicochemical filter” that reduces vulnerability to biotic stressors. These interpretations are well supported by contemporary reviews on wax composition and biosynthesis and on the roles of fatty alcohols in forming protective surface layers in plants [52,53,131,132]. For one long-chain fatty alcohol, docosanol (C22:0), substantial antibiofilm and antivirulence activity against bacteria has been demonstrated [133], suggesting a potential capacity of long-chain fatty alcohols to influence biofilm formation processes in macrophytes; however, direct evidence for aquatic plants is currently lacking.

Finally, C6 alcohols of the GLVs pool should be specifically mentioned. These compounds reached major component levels only in representatives of the genus *Potamogeton*: *P. perfoliatus* (hexan-2-ol, 1.04%, and (*E*)-hex-2-en-1-ol, 1.93%) and *P. pectinatus* ((*E*)-hex-2-en-1-ol, 7.71%) (Supplementary Materials, Table S1). In addition, other stress-associated volatile alcohols and alcohol-like compounds of different biosynthetic origin—such as the terpenoid alcohol geranyllinalool and the apocarotenoid β-ionol—were detected in comparable concentrations in *Potamogeton* species (Supplementary Materials, Table S1). GLVs are closely linked to tissue damage and oxidative stress and function as rapid stress-induced signals involved in inter-organismal communication [134,135,136]. Multiple protective roles have been demonstrated for GLVs in plants, including both direct defensive effects and signal-mediated modulation of defense responses, including antipathogenic activity [73,136,137]. It is therefore likely that, in aquatic environments, such low-molecular-weight volatile alcohols and functionally related stress-associated compounds can act and retain ecological relevance within the near-surface DBL surrounding macrophyte surfaces.

#### 2.3.3. Hydrocarbons

Among the major hydrocarbons identified, three n-alkanes showed the highest maximum relative contributions: tricosane (C23), pentacosane (C25), and heptacosane (C27) (Table 5). Moreover, in *M. spicatum*, *S.* × *foliosum, S. gramineum*, and *S. emersum*, these compounds were also included in the top three most abundant major LMWOCs by relative contribution (%) (Table 4).

Data reported in [138] indicate that the same n-alkanes also ranked among the three most abundant in submerged macrophytes, whereas in emergent plants they occupied the 2nd (heptacosane, C27), 3rd (pentacosane, C25), and 4th (tricosane, C23) positions.

Long-chain aliphatic hydrocarbons (primarily n-alkanes) play a pivotal role in the formation of cuticular waxes in aquatic macrophytes and generate a cuticular–waxy “first-contact layer” at the leaf/shoot–water interface, thereby affecting surface wettability [139,140]. In aquatic habitats, their ecological function is expressed mainly as a structural protective module that influences surface wettability, the DBL, and the likelihood of initial attachment of epiphytes and microorganisms, thus mediating plant interactions with periphyton, the surface microbiome, and phytoplankton [113,141,142,143]. Our results reveal a pronounced dominance signal of mid-chain n-alkanes (C23–C27), which is widely used to diagnose the contribution of submerged and floating macrophytes to organic matter pools in aquatic systems, in contrast to emergent aquatic plants and terrestrial vegetation [138,144]. This pattern is most evident in *Sparganium* species, where C23, C25, and C27 dominate (Table 5), consistent with an enhanced wax-barrier function [138,139]. In *M. spicatum* and *P. perfoliatus*, the representation of this structural-protective hydrocarbon block is somewhat lower than in *Sparganium* (Table 5; Supplementary Materials, Table S1), yet it remains substantial. This may suggest that, in species exhibiting high maximum hydrocarbon levels, a strategy of mitigating epiphyte-induced shading, maintaining tissue light regimes, and supporting photosynthetic performance could potentially be effective. By reducing “film-like” epiphytic pressure (shading plus competition for dissolved resources at the surface), macrophyte competitiveness increases and may indirectly contribute to the stability of the clear-water state in shallow aquatic ecosystems.

#### 2.3.4. Aldehydes

In the metabolomic profiles of the 11 investigated macrophyte species (Supplementary Materials, Table S1), major aldehydes were represented predominantly by lipid-derived compounds and exhibited strong species specificity. The major aldehydes included (i) GLVs/C6 aldehydes (primarily hexanal and (*E*)-hex-2-enal), (ii) medium- and long-chain aliphatic aldehydes (C9–C18; nonanal, pentadecanal, octadecanal, etc.), and (iii) the aromatic aldehydes benzaldehyde and 2-phenylacetaldehyde. The most distinct GLVs profile was observed in *P. perfoliatus*, where (*E*)-hex-2-enal reached 10.86%. Hexanal showed high relative abundances in several species and peaked at 5.08% in *C. demersum*. Among C9 aldehydes, nonanal was prominent (up to 7.58% in *S. gramineum*), whereas among C18 aldehydes, octadecanal reached 3.40% in *S. gramineum*. The long-chain aldehyde pentadecanal, detected in eight species, was particularly abundant in *M. spicatum* (5.82%), whereas in *N. lutea* it combined a moderate relative contribution (1.00%) with a high absolute concentration (74.28 μg·g^−1^), indicating a potentially substantial hydrophobic pool in tissues/surface-associated lipids. Aromatic aldehydes (benzaldehyde and 2-phenylacetaldehyde), as major components, were in our dataset primarily characteristic of the genus *Potamogeton*, peaking in *P. pectinatus* (benzaldehyde 2.27%; 2-phenylacetaldehyde 1.68%), whereas in *P. natans* they were not among major LMWOCs. Benzaldehyde as a major component was also recorded in *C. demersum* (Supplementary Materials, Table S1).

The particularly pronounced GLVs-type C6 aldehydes in *Potamogeton* species (together with other GLVs) (Supplementary Materials, Table S1) are characteristic of oxylipin-related chemistry and are consistent with the role of these compounds in rapid defensive responses. Direct antimicrobial activity and membrane-targeting effects have been reported for GLVs aldehydes, supporting their contribution to antifouling and the “chemical hygiene” of shoot surfaces in the periphyton zone [73,145,146].

Among the six GLVs considered, (3*E*)-hexenal ((*E*)-hex-3-enal) was reported as the most effective compound, showing a prominent bacteriostatic effect [145]. The major LMWOCs (*E*)-hex-2-enal detected in *P. amphibia* and *P. perfoliatus* (Supplementary Materials, Table S1) likely fulfill similar functions.

Second, in other taxa, C9–C18 aldehydes (nonanal, pentadecanal, octadecanal) predominate, plausibly reflecting an oxidative lipid/surface-wax component and potentially constraining the development of microbial biofilms [146,147,148], which classifies them as components of antifouling and surface protection in aquatic plants.

Aromatic aldehydes (benzaldehyde, 2-phenylacetaldehyde) may strengthen antibacterial and, potentially, algastatic pressure within the leaf boundary layer [149,150].

Thus, aldehydes constitute a reactive and “fast” fraction of the LMWM of aquatic macrophytes, linking plant stress physiology to community-level effects. At the ecosystem scale, this aligns with current concepts that allelopathy and chemical defense by aquatic macrophytes can reduce phytoplankton and/or epiphytes in shallow-water ecosystems, improve the light climate within plant stands, and promote the resilience of a clear-water state [10,11,151,152,153], while aldehydes most likely act as part of a multicomponent chemical “cocktail” [102,103,104].

#### 2.3.5. Esters

In the present study, within this group, we considered a set of compounds dominated by esters, as well as furans and other lipophilic oxygenated constituents assigned to this group under the adopted GC–MS classification.

Within the lipophilic “Esters” fraction, three main subsets can be distinguished: alkyl esters of fatty acids, terpenoid carboxylates, and furan-type oxygenates (Supplementary Materials, Table S1). Distinct interspecific contrasts were observed, which may be relevant for the chemical and functional structuring of the plant–water boundary layer. *P. natans* was most distinctive, with a strong dominance of the terpenoid carboxylates (methyl athecate (11.21%) and methyl sandaracopimarate (10.56%)), accompanied by ethyl octadeca-9,12-dienoate (2.50%). In *C. demersum* and several *Potamogeton* taxa, the profile shifted towards fatty-acid alkyl esters: methyl octadecanoate reached 6.28% in *C. demersum*, 4.54% in *P. pectinatus*, and 2.69% in *P. perfoliatus*. *M. spicatum* exhibited elevated maximum relative abundances of 3,7,11,15-tetramethylhexadec-2-enyl acetate (4.11%) along with increased furans (2-pentylfuran, 2.63%; 2-[(*Z*)-pent-2-enyl]furan, 1.6%), whereas in *N. lutea* and *P. amphibia* this fraction was largely furan-driven (~1.0–1.3%). In *Sparganium* spp. and *L. dortmanna*, “Esters” compounds did not reach the major-component threshold (>1%).

It has been shown that a range of lipophilic esters can be adsorbed/accumulated in natural biofilms [154]. By analogy, this suggests that ester-type metabolites produced by macrophytes may concentrate within epiphytic biofilms and modify their properties (e.g., adhesion and community composition), potentially affecting epiphyte load and, indirectly, the light regime.

Fatty-acid methyl esters (FAMEs) from marine microalgae exhibit antimicrobial activity against various Gram-positive and Gram-negative bacteria; their mode of action is linked to membrane damage and leakage of intracellular contents [155]. Moreover, these FAMEs significantly reduced the expression of genes associated with quorum sensing and biofilm formation [151,155]. Vijay et al. [156] further reported antibacterial, antivirulence, and antibiofilm activities of fatty-acid methyl esters.

Taken together, these observations support the hypothesis that ester-type components in macrophytes may modulate epiphytic communities and periphyton properties and, through changes in biofouling and light conditions, indirectly influence phytoplankton and ecosystem-level outcomes. However, this requires direct experimental validation.

The predominance of fatty-acid alkyl esters in *C. demersum* and *Potamogeton* species. may reflect membrane-active control of early biofilm colonizers through antibacterial and antifungal activities, including at low minimum inhibitory concentrations [157,158].

Finally, 2-pentylfuran has been described as a bioactive volatile metabolite in microbe–plant systems capable of stimulating the growth of the model plant *Arabidopsis thaliana* [159]. Its occurrence as a major constituent in six of the eleven studied macrophytes suggests that furans may act as potential signal-modulating components in aquatic macrophytes and within the epiphytic biofilm microzone; nevertheless, direct evidence for such roles in aquatic systems is still lacking.

Given that ester-type LMWOCs in aquatic macrophytes remain poorly characterized, the direction and magnitude of their effects are likely context-dependent (ester structure, concentration, and community composition). Therefore, any prediction for epiphyton in specific situations should be considered a species-specific hypothesis.

#### 2.3.6. Ketones

Ketones occurred as major components in 9 out of 11 macrophyte species. Two dominant blocks were identified: (i) apocarotenoid/ionone-like ketones and (ii) the isoprenoid ketone 6,10,14-trimethylpentadecan-2-one (phytone). The highest relative abundances of β-ionone were found in *C. demersum* (6.74%) and *P. perfoliatus* (5.28%), whereas phytone reached 9.09% in *P. pectinatus* and remained at 4.57–5.23% in *M. spicatum*, *P. amphibia*, and *C. demersum* (Supplementary Materials, Table S1).

Among all ketones, only β-ionone entered the Top 3 most abundant major LMWOCs (in *C. demersum*) (Table 4). A distinctive signal was the pronounced dominance of the steroid-like ketone androst-4-ene-3,6,17-trione in *P. perfoliatus* (13.13%). No major ketones were detected in *N. lutea* and *S. gramineum*. *L. dortmanna* contained the cyclic ketone cyclohexadec-8-en-1-one (4.75%).

Aquatic macrophytes have been shown to emit biogenic volatile organic compounds (BVOCs), including ketones [153], and in aquatic environments BVOCs (including apocarotenoid/ionone-like ketones) may function as chemical messengers and/or allelopathic signals affecting other phototrophs [160]. Ketones are therefore considered to have substantial allelochemical potential [160].

β-Ionone is a proven effective allelochemical and plant defense element: it has been experimentally shown to disrupt the photosynthetic system of *Microcystis aeruginosa* (including PSII-related targets), which is consistent with anti-cyanobacterial pressure exerted by macrophytes in waterbodies [151,161,162]. It has also been shown that β-ionone and other apocarotenoids comprise flavors, aromas, pigments, growth regulators, and defense compounds; serve as ecological cues; act as insect attractants or repellents; and display antibacterial and fungicidal properties [162,163]. β-Ionone is listed among major allelopathic agents that can induce strong inhibitory effects on phototrophs, up to lethal outcomes, by triggering programmed cell death [160,164].

It has been established that β-ionone and geranylacetone (identified as a major component in *P. perfoliatus*) are synthesized and released into the environment by red and green algae, presumably to control surrounding organisms in allelopathic interactions [165,166]. At the ecosystem level, such properties of apocarotenoids may contribute to reduced phytoplankton dominance and to the maintenance of a macrophyte-dominated state in waterbodies [7,60].

Phytone is a widespread BVOCs compound not only among plants but also among insects [167,168]. It exhibits allelopathic, antimicrobial, antifungal, and pheromonal activity [169,170,171]. Phytone is interpreted as a product/marker of photo- and oxidative processing of phytol chains (chlorophyll degradation), reflecting the intensity of light/oxidative stress and simultaneously having the potential to influence the microbiome/periphyton via BVOCs-mediated interactions [151,162,172].

For ketoisophorone, which is a major component in *P. perfoliatus* and *P. pectinatus* (Supplementary Materials, Table S1), the literature indicates multiple ecological functions. First, essential oils in which ketoisophorone occurs as a major or minor component exhibit conspicuous phytotoxic and allelopathic properties [173,174]. Second, this ketone is relevant to pollination ecology in plants and acts as a pheromone in insect mating behavior [175,176].

In aquatic ecosystems, ketoisophorone (4-oxoisophorone), together with other ketones, is regularly reported among the volatile organic compounds (VOCs) of aquatic phototrophs (micro- and macrophytes), and in some cases it has been described as a major ketone [27,95,177,178,179]. These observations are consistent with the possibility that, in water, ketoisophorone may serve as a marker of physiological status and stress-associated restructuring of macrophyte VOCs profiles and may participate in allelopathic interactions at the levels of the microbiome, periphyton, and phytoplankton.

Along with “classical” GLVs, which by definition are mainly C6 aldehydes, C6 alcohols, and their acyl esters [136], our dataset revealed low-molecular-weight volatile ketones as major LMWOCs—hexan-2-one, hexan-3-one, heptan-2-one, oct-7-en-2-one, and others (Supplementary Materials, Table S1). These compounds are better interpreted not as GLVs sensu stricto but as GLVs-like VOCs [136]. Notably, in our material, “green” alcohols (GLVs) reached major-compound levels only in representatives of *Potamogeton*, whereas ketone major VOCs were characteristic of other taxa as well (Supplementary Materials, Table S1). In aquatic ecosystems, such VOCs are considered potentially important infochemicals and markers of physiological status [151]. An additional argument for their natural relevance is that, in a freshwater enrichment culture “algae–bacteria”, hexan-2-one and hexan-3-one were directly detected as exometabolites with a possible allelopathic role [180]. Defensive, repellent, and antibacterial properties have been described for 2-heptanone and its derivatives, including in red algae [181,182,183].

More generally, experimental evidence indicates that odor-active VOCs blends of cyanobacterial biofilms, including ketones, can serve as chemical cues in aquatic environments (demonstrated using nematodes), i.e., VOCs have real ecological functions in micro- and periphyton habitats [184].

Finally, the identified major occurrence of androst-4-ene-3,6,17-trione in *P. perfoliatus* requires careful analysis and verification. However, the occurrence/conversion of progestogens/androgens in plants, the involvement of aquatic plants in the fate of hormone-like micropollutants, and active microbial degradation of steroids make its contribution to biofilm restructuring and cascading effects on periphyton and phytoplankton ecologically plausible [185,186].

Overall, ionone-like and isoprenoid ketone profiles in aquatic macrophytes provide a basis for chemically mediated pressure on phototrophic and microbial compartments (periphyton/phytoplankton), which—other factors being equal—may shift aquatic systems toward a more “macrophyte” state.

#### 2.3.7. Aromatic Hydrocarbons

Only two aromatic hydrocarbons (AH) reached the level of major compounds in the macrophytes studied: 1,2-xylene (o-xylene) was detected in *P. perfoliatus* with a maximum relative abundance of 1.12%, and 1-methyl-7-(propan-2-yl)phenanthrene (retene) was detected in *P. amphibia* with a maximum relative abundance of 1.00% (Supplementary Materials, Table S1). In the remaining studied species, aromatic hydrocarbons were not recorded as major compounds.

It is important to note that, for AH, the duality of possible origin is critical: detection in plant samples may reflect (i) a plant-associated LMWOCs pool [80], but also (ii) sorption/re-emission from water and bottom sediments, inputs from the catchment/atmosphere, and anthropogenic sources [187,188,189,190]. It has been estimated that the global potential of secondary organic aerosol formation from biogenic plant benzenoids is approximately comparable to that from anthropogenic benzenoids [191]. Experimentally, inhibition of the freshwater green alga *Selenastrum capricornutum* by volatile aromatic hydrocarbons (including xylene components in mixtures), with possible additive and synergistic effects, has been demonstrated [192], which is consistent with the possibility of local “chemical pressure” within the boundary layer of macrophyte stands.

Retene may originate from vascular plants, as well as possibly from algae and cyanobacteria [193,194]. Retene can also be regarded as a pyrogenic/terrigenous PAH marker associated with inputs from wood combustion and/or catchment flows, as well as with potential accumulation in areas impacted by the pulp and paper industry [195]. This is particularly relevant for Lake Ladoga, where our *P. perfoliatus* samples were collected in different habitats and whose catchment hosted active pulp and paper mills [196].

Given these considerations, interpreting o-xylene and retene as major “plant metabolites” is possible, but it requires caution and rigorous experimental verification, while their biological effects on aquatic organisms remain relevant regardless of origin [151,189].

Thus, conceptually, a biogenically associated contribution of AH from vegetation in general to processes in aquatic ecosystems is plausible [191]; however, the mere occurrence of o-xylene and retene as major components of the LMWM in *P. perfoliatus* and *P. amphibia* does not constitute evidence of their purposeful biosynthesis and allelopathy.

#### 2.3.8. Diverse Functional Groups

In the “Diverse functional groups” class, based on maximum relative abundances (Supplementary Materials, Table S1), the most pronounced contribution was observed in *Potamogeton* species, whereas in other taxa (*M. spicatum*, *Sparganium* spp., *N. lutea*, *P. amphibia*, *L. dortmanna*) the contents of LMWOCs assigned to this class were below the threshold for major components (<1% of total LMWOCs). In *P. natans*, the highest value was recorded for a steroidal acetate (10.19%), and relatively high values were also observed for polyalthic acid (6.31%). In *P. perfoliatus*, high proportions were found for cyclopenta[a]phenanthrene-type derivatives with retention index (RI) = 2244 (pregnanolone-like steroid; tentative library ID, 7.59%) and RI = 2467 (tentative library ID, 5.20%). In *P. pectinatus*, the leading component was sandaracopimarinol (7.35%), accompanied by diterpene esters (methyl daniellate 2.10%**,** methyl lambertianate 1.41%). In *C. demersum*, a moderate contribution of a pregnanolone-like steroid (tentative library ID, 1.67%) was observed (Supplementary Materials, Table S1).

The ability of plants to carry out biotransformations of pregnane steroids and to form pregnanolone-like derivatives is supported by experimental evidence and direct findings in specific plant objects [197,198,199,200]. However, under GC–MS library identification without confirmation by an authentic standard, the stereochemistry of steroids is generally not verified; therefore, the steroid peaks reported above are more appropriately interpreted as pregnanolone-like (tentative ID).

It should also be noted that steroid derivatives in general (including ecdysteroids in some plants) may contribute to antifeedant defense and can function as molting and metamorphosis hormones in crustaceans and insects when they consume plants [80,201,202,203].

Occurrences of sandaracopimarinol, a typical resin-derived diterpene alcohol, have been documented mainly in coniferous/cypress taxa (*Tetraclinis articulata*, *Cryptomeria japonica*, *Pinus nigra*) [204,205,206,207]. This compound, within a hexane heartwood extract, was shown to inhibit the growth of the cyanobacterium *Microcystis aeruginosa* [205].

Polyalthic acid (a furanolabdane diterpene/labdane-type diterpenoid acid) is reliably described in plants as an isolated and structurally characterized natural product; its stereochemistry was established in a classical study on *Polyalthia fragrans* (Annonaceae) [208]. This compound has also been isolated from *Vitex rotundifolia* (Lamiaceae), described as a “major stable compound” in *Daniella oliveri* (Fabaceae), and characterized as bioactive [209,210].

The potential allelopathic relevance of compounds within the “Diverse functional groups” class appears to be particularly pertinent to Potamogetonaceae. Specifically, ent-labdane diterpenes have demonstrated effects on algae (*Selenastrum capricornutum*) and other aquatic organisms [125], and specific antialgal diterpenes/lactones have been described from *P. natans* [56,211]. Accordingly, in *P. natans*, the combination of a relatively high share of polyalthic acid (6.31%) with abundant steroidal esters (10.19%) implies a potentially substantial chemically mediated constraint on the microphyte component (phytoplankton/epiphytic algae) and/or the bacterial phase of biofilms. This is consistent with the reported broad antimicrobial profile of polyalthic acid [212,213].

For *P. pectinatus*, the possible presence of sandaracopimarinol with a high contribution to the LMWM (7.35%), together with reports of ent-labdanes in this species showing strong algicidal activity against *Raphidocelis subcapitata* [214,215], supports the hypothesis that a complex of lipophilic terpene compounds may influence periphyton communities. For *P. perfoliatus*, the high contribution of steroid derivatives (7.59% and 5.20%; tentative IDs) may plausibly reflect both stress adaptation and involvement in regulating the microenvironment near plant surfaces (leaves, stems).

Lipophilic steroid–terpenoid components are most functionally relevant at the plant surface–water interface, where they can potentially affect biofilm/periphyton development and, indirectly, competitive interactions with phytoplankton [7,10,20]. Overall, the present data support the hypothesis that *Potamogeton*-dominated stands, as well as stands formed by other macrophytes, may contribute to clear-water states via chemically mediated weakening of periphyton and/or phytoplankton [10,56,125,211].

#### 2.3.9. Sulfur-Containing LMWOCs

The last group in which major compounds were detected was sulfur-containing LMWOCs. Within this “sulfur” group, GC–MS revealed three major compounds that were confined to a limited number of taxa. 1,3-benzothiazole was recorded in *P. amphibia* (2.09%) and *P. perfoliatus* (1.05%). Two lipophilic S-esters—pentadecyl propan-2-yl sulfite and octadecyl propan-2-yl sulfite—were found only in *Sparganium*: in *S.* × *foliosum* (1.59% and 2.19%, respectively) and in *S. gramineum*, where octadecyl propan-2-yl sulfite reached a maximum of 10.367%. In the LMWM of the remaining species, sulfur-containing components were not detected as major constituents.

1,3-Benzothiazole is considered a naturally occurring volatile and is a widespread constituent of essential oils in terrestrial plants, where it is primarily associated with signaling functions [216,217,218]. In a study on allelopathic exudates of two *Potamogeton* species [62], the authors explicitly reported benzothiazole (as well as 2-(methylthio)-benzothiazole) among compounds identified by GC–MS in the most active lipophilic fraction (CH_2_Cl_2_ extract) of incubation water that inhibited *Selenastrum capricornutum* and a toxic strain of *Microcystis aeruginosa*. Importantly, these compounds were not detected in the culture-medium control, yet were present in treatments with macrophytes at some of the highest relative abundances. This pattern indicates a direct association with the macrophytes (*Potamogeton maackianus* and *Potamogeton malaianus*) and supports considering benzothiazole as a candidate compound involved in chemical interactions between macrophytes and microalgae/cyanobacteria (an allelopathic “candidate”). However, the relative contribution of plant metabolism versus microbial transformation requires additional verification (further experimental work). Despite this caveat, there are grounds to regard benzothiazole as an endogenous metabolite of freshwater macrophytes with potential signaling and allelopathic functions.

Regarding pentadecyl propan-2-yl sulfite and octadecyl propan-2-yl sulfite, there is evidence that these compounds occur in GC–MS profiles of essential oils and extracts from terrestrial plants, including *Isodon rugosus* (Lamiaceae) and *Gomphrena globosa* (Amaranthaceae) [219,220]. Moreover, octadecyl propan-2-yl sulfite from *G. globosa* has been reported to exhibit antibacterial activity [221].

Natural sulfur-containing plant metabolites have been reported to exhibit antimicrobial activity against various bacterial and fungal pathogens, making them promising candidates for antibiotic development [222]. Furthermore, many of them exhibit the ability to induce apoptosis and inhibit tumor cell proliferation [222].

It was noted that biogenic sulphides produced by charophytes may be most effective at the cell-surface boundary of these macroalgae [223]. The same author emphasized that, in general, little is known about the chemical ecology of biogenic sulfur compounds in freshwater systems [223]. At the same time, recent studies indicate that, in terrestrial plants, organic sulfur compounds can exert allelopathic as well as antibacterial activities [224,225,226]. For aquatic ecosystems, it has been suggested that such compounds most likely have signaling roles and may also act as allelochemicals [7,93,223].

Overall, the patterns identified for major LMWOCs across different chemical classes support the view that these compounds primarily exert ecologically meaningful effects within DBL-mediated microenvironments at the plant–water interface and on macrophyte surfaces, where small-scale gradients and frequent contact with epiphytes and the microbiome may enable functionally relevant metabolites to produce community-level effects. At the same time, allelopathic effects of macrophytes on phytoplankton at the scale of entire shallow-water ecosystems—together with other mechanisms that promote a ‘clear-water’ ecosystem state and that have been well documented across studies [1,2,3,4,7,9,10]—also indicate the functional relevance of macrophyte metabolites beyond the DBL.

## 3. Materials and Methods

### 3.1. Plant Material

In this study, we analyzed the major components of the LMWM of the following widely distributed freshwater macrophytes: *Myriophyllum spicatum* L., *Sparganium emersum* Rehm., *Sparganium gramineum* Georgi, the hybrid *Sparganium* × *foliosum* A. A. Bobrov, Volkova, Mochalova et Chemeris, *Persicaria amphibia* (L.) Delarbre, *Potamogeton perfoliatus* L., *Nuphar lutea* (L.) Sibth. & Sm., *Potamogeton pectinatus* L., *Potamogeton natans* L., *Lobelia dortmanna* L., and *Ceratophyllum demersum* L.

Macrophyte samples were collected from diverse habitats in several water bodies: lakes in the Novgorod, Moscow, Tver, and Yaroslavl regions (Russia) (*S. emersum, S. gramineum, S.* × *foliosum, L. dortmanna*); lakes of the Karelian Isthmus, Northwestern Russia (*N. lutea, P. natans*, *M. spicatum*); Lake Ladoga (*P. perfoliatus, P. pectinatus, P. amphibia, M. spicatum*); floodplain lakes of the Volga–Akhtuba floodplain (Astrakhan Region, Russian Federation) (*P. pectinatus*, *C. demersum*); and Lake Naroch (Belarus) (*M. spicatum*) (Table 6).

Plant material was collected at the peak of the growing season (late July–early August) over the course of different years from 2010 to 2023 in the above-mentioned water bodies. In compliance with [227], the harvested plants were thoroughly rinsed to remove epiphytes and other attached materials. A sufficient number of shoots was collected so that the dry-weight equivalent (at least 50–100 g) was adequate to prepare an integral sample for gas chromatography–mass spectrometry (GC–MS), i.e., a composite sample comprising different shoots of the plant collected from the same habitat.

The plant material was air-dried to constant weight in a shaded, darkened area indoors or under a canopy, protected from direct sunlight, at temperatures not exceeding 35 °C. Such a traditional way of drying in the shade is regarded to be the most suited [228,229]. The dried samples were packed in dark bags and stored in the laboratory at room temperature under dry conditions, protected from sunlight. Subsequent GC–MS analysis of the samples was performed no earlier than one month and no later than one year after collection and drying, in accordance with regulatory guidelines for the storage of plant raw materials [230].

Below, we provide a general ecological and biological characterization of the studied macrophyte species.

#### 3.1.1. *Myriophyllum spicatum* L.

*M. spicatum* (Eurasian watermilfoil) is a perennial, fully submerged macrophyte of the family Haloragaceae, producing long stems (up to several meters) and whorled, finely dissected leaves; reproductive shoots form an emergent terminal spike, and the small flowers appear to be predominantly wind pollinated. The species combines sexual reproduction (by seeds) with a prominent capacity for vegetative spread: stem fragmentation and subsequent rooting of fragments are regarded as one of the key mechanisms enabling rapid expansion of stands, and its seasonal dynamics and “life strategy” (including overwintering and resumption of growth) have been described in detail for temperate-zone populations [231,232,233,234]. The native range of *M. spicatum* is associated with Eurasia and North Africa; however, during the 20th century the species became widely dispersed and naturalized beyond its original range and is now recorded across the Northern Hemisphere—in Europe, Asia, North America, and North Africa; it has also been reported from eastern Africa (Somalia) and insular areas in the western Pacific (the Philippines) [234]. For North America, its introduction history and spread across the United States and Canada were summarized in a classic paper [235]. Ecologically, Eurasian watermilfoil is primarily associated with still and slow-flowing waters (lakes, ponds, reservoirs, and low-energy river reaches), can grow across a range of littoral depths (up to ~10 m where light is sufficient), shows broad tolerance to environmental conditions with respect to temperature, pH and salinity (including persistence in slightly brackish waters), and often performs well in water bodies experiencing anthropogenic impacts and elevated trophic status [232,236,237].

#### 3.1.2. Genus *Sparganium* L.

The genus *Sparganium* (Typhaceae) comprises rhizomatous perennial aquatic macrophytes with pronounced phenotypic plasticity; the flowers are unisexual and arranged in globose heads, and the persistence and expansion of stands are ensured both by seed reproduction and by active clonal spread via creeping rhizomes [238,239,240]. *S. emersum* is a widely distributed taxon across Eurasia and western North America, typically associated with shallow zones of lakes and with calm/slow-flowing sections of rivers and channels; shoot and leaf morphology varies along depth and hydrodynamic gradients [238,239,241]. *S. gramineum* is more habitat-restricted: in European Russia it is described as a long-rhizomed polycarpic perennial occurring mainly in the littoral zone of mesotrophic (more rarely oligotrophic) waterbodies; in northern European soft-water lakes it is listed among typical components of macrophyte communities associated with low mineral content and high water transparency [242,243]. The hybrid *S. × foliosum* is regarded as the nothotaxon of the parental pair *S. emersum × S. gramineum*, diagnosable by a suite of intermediate characters and occurring predominantly in areas where the parents co-occur [44,45].

#### 3.1.3. Genus *Potamogeton*

Macrophytes of the genus *Potamogeton* (Potamogetonaceae) are a cosmopolitan group of aquatic plants, predominantly submerged, rooted hydrophytes with creeping rhizomes, high morphological plasticity, frequent hybridization and polyploidy; the genus is characterized by spike-like inflorescences and diverse overwintering/vegetative renewal strategies (including specialized winter buds/turions), which together confer broad ecological amplitude and a major role in structuring shallow-water communities [55,244,245,246]. *P. perfoliatus* is a submerged, rooted macrophyte of the shallow littoral zone and an important component of lake/pond communities; this species often supports substantial epiphyton accumulation on its leaves, which can markedly alter plant photobiological traits [65,244,247]. Consequently, careful cleaning of shoots is required when analyzing its LMWOCs. The native range of the species spans the temperate Northern Hemisphere and extends to Sumatera and eastern/southeastern Australia [246]. *P. pectinatus* (in many modern treatments, *Stuckenia pectinata* (L.) Börner) is a submerged, narrow-leaved “sago pondweed” that is nearly cosmopolitan [246] and can tolerate elevated salinity and irradiance; these traits make it a characteristic species of fresh to slightly brackish, often eutrophic habitats [248]. *P. natans* (floating-leaved pondweed) is a perennial hydrophyte bearing both floating and submerged leaves, typical of shallow zones of standing and slow-flowing freshwaters (occasionally slightly brackish); it is native to the temperate and subtropical regions of the Northern Hemisphere [246,249].

#### 3.1.4. *Persicaria amphibia* (L.) Delarbre

*P. amphibia* (Polygonaceae; synonym *Polygonum amphibium* L.) is a widely distributed amphibious perennial helophyte, native across subarctic and temperate parts of the Northern Hemisphere and also recorded from parts of Africa (Ethiopia–Kenya and South Africa) [250,251]. It is rhizomatous/stoloniferous and highly plastic, producing both an aquatic rooted floating-leaved morph and a terrestrial/emergent shoreline morph; the extent of habitat-driven variation is commonly treated as the “*P. amphibia* complex” [76,252,253]. Ecologically, the species is typical of shallow littoral zones of lakes and ponds, river/stream margins, and wet floodplain habitats, and it tolerates strong water-level fluctuations and drawdown, which explains its success in dynamic shoreline environments [76,250,253].

#### 3.1.5. *Nuphar lutea* (L.) Sm.

*N. lutea* is a perennial rooted macrophyte with floating leaves and a stout rhizome, widely distributed across the Palearctic [254,255]. The species is characterized by distinct heterophylly (floating and submerged leaves), clonal growth forming patches/stands, and solitary yellow flowers at the water surface [256]. It typically occurs at depths of 0.5–1 m in lakes as well as in ponds, reservoirs, backwaters, and slow-flowing rivers, and it is sensitive to water pollution [254]. Ecologically, *N. lutea* is typical of water bodies with soft bottom sediments, most often in shallow littoral zones. Its functional traits and production/biogeochemical parameters vary substantially among water bodies differing in trophic status and habitat conditions [257,258]. As a habitat-forming (structuring) species, the yellow water lily often forms plant associations together with other aquatic macrophytes [259].

#### 3.1.6. *Lobelia dortmanna* L.

The species’ range includes Northern, Central, and Eastern Europe as well as North America. It is considered a relict species and is rare throughout its distribution. It grows in the littoral zone of freshwater waterbodies on clean sandy bottoms, typically at depths of about 60–80 cm. Plants occur singly or in small groups and rarely form extensive stands. The species is sensitive to water-level fluctuations. A major limiting factor is waterbody pollution; therefore, *L. dortmanna* is regarded as an indicator of water purity [260]. Ecologically, it is associated with soft-water, low-mineralization oligotrophic to weakly mesotrophic lakes with a well-lit shallow zone and mineral (usually sandy), low-organic substrates, and it is sensitive to siltation and organic enrichment followed by sediment deoxygenation [261,262]. One of the species’ main physiological adaptations is active root uptake of CO_2_ from sediment pore water to support photosynthesis, which is especially important in clear oligotrophic lakes [82,263].

#### 3.1.7. *Ceratophyllum demersum* L.

*C. demersum* (Ceratophyllaceae) is a widely distributed (cosmopolitan) freshwater submerged macrophyte, either free-floating or loosely anchored to the substrate by thin rhizoid-like shoots arising from the stem base. The plant acquires nutrients directly from the water column [254,264]. It develops long, brittle stems and whorled leaves that are deeply dissected into narrow segments (often bearing small teeth/spines), which increases the exchange surface with the surrounding water and promotes efficient gas and ion exchange in the water column [55,265]. Ecologically, the species is most typical of still and slow-flowing freshwater habitats (lakes, ponds, canals, oxbow lakes and river backwaters). Plants often form large monospecific stands that may extend down to ~10 m depth under sufficient light. Growth of *C. demersum* has been reported at depths where only about 1% of surface irradiance is available. Its high shade tolerance is consistent with a low light compensation point and the capacity to remain functional beneath ice in temperate lakes. Stands of *C. demersum* may have a strong competitive advantage over free-floating competitors, in part by elevating water pH during intense photosynthesis [55,254,264,266,267].

### 3.2. Sample Extraction

Essential oils containing LMWOCs were obtained from dried plant material. A weighed portion of the sample was 10–15 g of dry plant material. Before distillation, the dried plant material was crushed into a powder using a Waring BB-25ES blender (Waring Commercial, Stamford, CT, USA). The generally accepted Clevenger method (a method of steam hydrodistillation) was used for extracting LMWOCs from plant material samples by distilling the samples with water vapor for 6 h at 100 °C [268,269,270,271]. The resultant distillate was extracted with 5 mL hexane (CAS 110-54-3; Cryochrom, Saint Petersburg, Russia). Hydrodistillation using a Clevenger-type apparatus (JSC LenReactiv, Saint Petersburg, Russia) was selected for extracting LMWOCs from dried plant material because this procedure is an officially recommended method for obtaining essential oils from dry botanical raw materials [269,270,272]. In addition, as noted by A.V. Tkachev [273], steam distillation of essential-oil-bearing plant material enables a sufficiently complete recovery of essential oils in a relatively unaltered form, provides the lowest experimental error, and is particularly suitable when the oil content is low; moreover, because the receiver is positioned outside the distillation flask, the method also allows the determination of thermolabile essential-oil components in the raw material. Accordingly, steam distillation remains the most widely used approach for essential oil isolation [273]. We further employed a modification in which an organic solvent (hexane) was added directly to the Clevenger receiver. This adjustment mitigates a known limitation of the method, namely that a substantial fraction of ecologically relevant LMWM constituents with densities close to or exceeding 1.0 g·cm^−3^ may remain in the aqueous phase [273]. The hexane extract for subsequent chromatography mass spectrometric analysis was kept in hermetically sealed vials in a freezer at −18 °C.

### 3.3. GC-MS Analysis

GC–MS was used to determine the qualitative and quantitative composition of LMWOCs. Analyses were performed using (i) a SHIMADZU GCMS-QP2010 Ultra GC–MS system (Shimadzu Corporation, Kyoto, Japan) equipped with an MTX-1 non-polar capillary column (30 m × 0.25 mm i.d., 0.25 μm film thickness) and (ii) a Thermo Scientific TRACE ISQ GC–MS (Thermo Fisher Scientific, Waltham, MA, USA) system equipped with a TRACE TR-5MS capillary column (15 m × 0.25 mm i.d., 0.25 μm film thickness). Helium was used as the carrier gas. Mass spectra were acquired in full-scan mode over the *m*/*z* range 30–1090. The oven temperature program was as follows: 35 °C (3 min), ramp at 2 °C/min to 60 °C (hold 3 min), ramp at 2 °C/min to 80 °C (hold 3 min), ramp at 4 °C/min to 120 °C (hold 3 min), ramp at 5 °C/min to 150 °C (hold 3 min), and ramp at 15 °C/min to 240 °C (hold 10 min). Chromatograms were processed step-by-step after data acquisition. Compounds were identified using the NIST 2014 and Wiley mass spectral libraries [274,275]. For more accurate identification, linear retention indices were used [273,276], calculated using a C7–C30 n-alkane standard mixture. Quantification was performed using benzophenone as an internal standard.

Compound diversity was assessed by qualitative analysis, i.e., by compound identification. Identification reliability was evaluated using the library match factor (Match) and reverse match factor (R.Match); for the compounds reported in this study, these values were typically ≥800–900 (good agreement), and for many of the most abundant compounds (i.e., the major components emphasized in this work) they exceeded 900 (excellent agreement). Quantitative analysis was performed using benzophenone (Acros Organics, Geel, Belgium; EEC No. 204-337-6; CAS No. 119-61-9) as an internal standard, enabling determination of the concentrations of the identified compounds.

Major components of LMWM were operationally defined as compounds contributing >1% of the total signal (based on peak area relative to the sum of peak areas of all detected LMWOCs). This threshold is commonly used in GC–MS profiling to distinguish dominant constituents from trace compounds and to improve comparability among samples [36,277,278].

When compiling the final lists of LMWOCs for the studied species, including the relative content and concentrations of major components, we used the maximum observed values of relative content and concentration for each component recorded for each species. Thus, the resulting final lists reflect the highest possible level (i.e., the potential) of relative content and the highest possible level (i.e., the potential) of concentration of particular major components in the LMWM of a given macrophyte species. Quantitative comparisons among the studied macrophyte species were performed using these maximum LMWOCs values.

### 3.4. Similarity Assessment and Statistical Analyses

The similarity of the sets of major components in the studied macrophyte species with respect to the qualitative composition of LMWOCs was assessed using the Jaccard similarity coefficient (J) and the Sørensen–Czekanowski coefficient (Qs) [279,280,281], calculated as:

$$J=\frac{c}{a+b-c};\;Qs=\frac{2c}{a+b},$$

where *c* is the number of LMWOCs common to samples A and B; *a* is the number of LMWOCs present in A; *b* is the number of LMWOCs present in B.

Similarity estimates between samples based on quantitative data (i.e., the abundances of individual compounds) were obtained using the Morisita (Morisita–Horn) index [282]:

$$Cmh=\frac{2\sum_{i}\left(an_{i}\cdot bn_{i}\right)}{(da+db)\cdot aN\cdot bN},$$

where *an_i_* is the abundance of the *i*-th compound (or compound group) in sample A; *bn_i_* is the same for sample B; (*aN*) is the total abundance of LMWOCs in sample A; (*bN*) is the same for sample B; *da* = ∑(*an_i_*^2^)/*aN*^2^, *db* = ∑( *bn_i_*^2^)/*bN*^2^.

J and Qs describe overlap in the set of major compounds, whereas the Morisita–Horn index emphasizes dominant molecules and is therefore more sensitive to abundance structure. Moreover, the Morisita–Horn index does not depend on sample size and diversity and thus has a significant advantage [84].

To estimate the mean content of different chemical groups of major LMWOCs in the LMWM of the studied species, arithmetic means and standard errors of the mean were calculated. The relative variability of the chemical group contents with respect to their mean values was assessed using the coefficient of variation (CV). For the interpretation of the coefficient of variation, a harmonized scale was applied: <10%—very low variability, 10–20%—low, 20–30%—moderate, 30–50%—high, and >50%—very high. The use of this scale was based on publications employing the Pimentel-Gomes classification, while the threshold of CV >50% was adopted from studies assessing climatic and hydrometeorological variability [283,284,285]. Statistical analyses were performed using STATISTICA software, version 10 [286].

## 4. Conclusions

The comparative analysis of the major fraction of the LMWM in 11 freshwater macrophyte species revealed pronounced interspecific differentiation while maintaining a shared functional “core” of LMWOCs. In total, 137 major LMWOCs were recorded (four remained unidentified), and their numbers differed markedly among species (from 11 to 71), indicating distinct ecological-biochemical strategies in organizing the principal LMWM pool that constitutes the most abundant and, likely, ecologically relevant component of chemical activity of a given macrophyte in aquatic ecosystems and its adaptation to specific environmental conditions.

Fatty acids represented the most universal dominant module in macrophytes. They were among the top three major components in all species and ranked first or second (or even all three) in 10 of 11 species. This supports the interpretation of the lipid module as a key hub linking (i) tissue structure and surface barrier fractions, (ii) stress signaling (oxylipin cascades), and (iii) potential allelopathic effects on phytoplankton, periphyton, and the microbiome.

The results of a comparison of the similarities/dissimilarities of the studied species based on their major LMWOCs are important. Species similarity depended on what was compared—compound “sets” or “dominance”. Similarity matrices demonstrated a fundamental divergence between similarity based on the presence/absence of compounds and similarity based on dominant molecules (Jaccard/Sørensen–Czekanowski vs. Morisita–Horn indices). Ecologically, this implies that different species may possess distinct lists of major metabolites while exhibiting a similar quantitative metabolomic architecture driven by shared dominant classes/compounds—i.e., the most plausible candidates to reach biologically meaningful concentrations. This, in turn, may enable the implementation of similar adaptive strategies in aquatic ecosystems and the capacity to modify the surrounding water environment in a favorable direction.

Overall, our study provides a basis and a comparable interspecific framework for interpreting the chemical regulation of aquatic communities in macrophyte-dominated habitats, focusing on the major fraction as the most likely driver of ecosystem-level effects, including those expressed in the microenvironment of the DBL. At the same time, we show that the “chemical profile” of a macrophyte should be considered not only as a list of compounds but also as a dominance structure, which may converge across ecologically and taxonomically different plants.

From a theoretical perspective, our findings refine current understanding of how macrophytes shape the chemical component of shallow-water ecosystem functioning and influence other aquatic organisms via dominant metabolic modules that are most likely to exert effects within the plant’s DBL and the water column. From a practical perspective, we compiled a set of priority compound classes and candidate species for targeted tests of anti-cyanobacterial/anti-fouling activity and for monitoring the functional state of macrophyte communities.

In addition, our study highlights the prominent role of fatty acids as major constituents and supports their potential as allelochemicals for incorporation into composites of next-generation algicidal preparations [81,99] aimed at mitigating harmful algal blooms (HABs) caused by unwanted algae and cyanobacteria in aquatic ecosystems. Composites combining fatty acids with other allelochemicals may be developed on the basis of dedicated experimental evidence and tailored to enhance activity against particular bloom-forming taxa. The accumulation of the necessary theoretical and applied knowledge on aquatic-plant allelochemicals may facilitate the development of a convergent, nature-inspired technology for HAB prevention based on next-generation algicides composed of allelochemical substances that are either synthetic analogues or natural macrophyte metabolites extracted from plant biomass. The environmental compatibility of such an approach may be supported by its conceptual grounding in naturally occurring allelopathy, one of the mechanisms contributing to the maintenance of a clear-water state in natural water bodies. Implementation of this technology may enable more advanced strategies for phytoremediation and the rehabilitation of impacted aquatic ecosystems. Allelochemical-based HAB mitigation approaches may help maintain high water quality, support recovery after disturbance, and promote the sustainable use of freshwater resources across practical sectors. In this context, plant-allelochemical-based next-generation algicides may be considered a potentially effective alternative to conventional algicides, which often cause substantial adverse impacts on aquatic ecosystems [81,287,288]. Finally, our interspecific comparison of dominant metabolites across freshwater macrophytes may contribute to optimizing the selection of macrophyte taxa for phytoremediation.

Several limitations should be acknowledged. First, our analysis focused on major components (>1% of the total LMWOCs pool), whereas minor metabolites may exhibit high bioactivity even at low concentrations; therefore, the biological significance of macrophyte metabolites is not fully captured by “majority” criteria alone. Second, interspecific comparisons were performed using the maximum observed values of each compound within the LMWM (as an estimate of species “potential”). This approach improves comparability in terms of upper boundaries but does not replace assessments of typical (mean/median) levels of LMWOCs in macrophytes and their actual fluxes to the environment. This should be the subject of future research. Our inferences regarding potential ecological functions of LMWOCs are based on chemical profiles and literature-driven interpretation and require direct verification using bioassays and field measurements of concentrations/gradients in the water and at plant surfaces.

Our results further highlight that the number of potentially relevant metabolites is large; hence, investigating ecological–biochemical functions of macrophyte LMWOCs requires a prioritization strategy when designing and conducting experimental studies. Even the number of major LMWOCs, as shown here, is substantial. To identify the principal regulatory mechanisms in aquatic ecosystems, it may be reasonable to focus primarily on major compounds. However, even in this case, the required research effort remains considerable. Importantly, such work should be embedded within an integrated research framework combining sequential laboratory studies, experiments with mesocosms in natural water bodies, and, subsequently, whole-ecosystem experiments. Only such an approach can resolve the real ecological roles of macrophyte LMWOCs and their place within the complex regulatory matrix of aquatic ecosystems. This workload may be partially reduced by QSAR studies [101,199], which can predict biological activities of LMWOCs without extensive “wet-lab” screening across large compound sets. Following QSAR-based identification of the most promising molecules, they can be examined in a more targeted manner, with reduced effort and resources, to confirm and characterize specific chemical–ecological functions.

Future research directions that appear particularly promising include: (i) moving from “within-organism” LMWOC profiles to measurements of the exometabolome and compound concentrations within the DBL at plant surfaces and in the water column; (ii) integrating endo- and exometabolomic chemical profiles with analyses of the microbiome/periphyton/phytoplankton and hydrodynamics (effects on transport and transformation); (iii) targeted bioassay-based experiments (especially at the whole-ecosystem level) for dominant classes, primarily fatty acids and other highly active allelochemicals; and (iv) assessing seasonal and interannual variability in macrophyte LMWOCs and shifting from “potential” (max) to “realized impact”.

## Figures and Tables

**Table 1:** Groups of major LMWOCs of the LMWM of the studied macrophyte species and the number of major LMWOCs.

Compounds	%			C			*n*		
	Mean	SEM	CV	Mean	SEM	CV	Mean	SEM	CV
*Sparganium gramineum*									
Alcohols	5.15	1.31	0.44	5.38	0.60	0.19	1	0	0.40
Aldehydes	8.84	2.35	0.46	10.87	4.97	0.79	3	1	0.29
Hydrocarbons	39.75	3.78	0.16	46.46	11.09	0.41	5	1	0.37
Fatty acids	14.51	5.82	0.69	14.19	4.09	0.50	2	1	0.67
Sulfur-containing	4.83	2.61	0.93	6.77	4.40	1.12	1	0	0.67
TOTAL/N = 20	73.08	1.18	0.03	83.67	17.06	0.35	13	1	0.15
*Sparganium emersum*									
Alcohols	13.02	0.05	0.004	14.20	1.36	0.10	1	0	0.00
Aldehydes	2.68	0.19	0.071	2.93	0.50	0.17	2	0	0.00
Hydrocarbons	48.10	9.92	0.206	53.03	16.03	0.30	6	1	0.13
Fatty acids	11.70	8.80	0.752	12.30	8.33	0.68	1	0	0.00
Ketones	1.97	0.91	0.463	2.10	0.78	0.37	1	0	0.00
TOTAL/N = 13	77.47	0.36	0.005	84.56	8.77	0.10	11	1	0.07
*Sparganium* × *foliosum*									
Alcohols	9.17	1.24	0.30	12.70	1.91	0.34	1	0	0.61
Aldehydes	5.19	1.24	0.53	6.68	0.91	0.31	2	0	0.19
Hydrocarbons	49.83	3.92	0.18	71.85	10.92	0.34	6	1	0.21
Fatty acids	7.67	2.78	0.81	10.68	3.62	0.76	1	0	0.35
Sulfur-containing	0.89	0.69	1.73	1.17	0.99	1.89	1	0	1.67
Ketones	0.79	0.41	1.15	0.93	0.46	1.10	1	0	1.10
TOTAL/N = 25	73.53	1.96	0.06	104.00	10.85	0.23	11	1	0.15
*Potamogeton pectinatus*									
Alcohols	15.16	0.67	0.06	19.56	8.23	0.59	4	0	0.00
Aldehydes	5.45	4.02	1.04	6.70	4.11	0.87	2	1	0.89
Hydrocarbons	9.13	3.58	0.56	9.92	2.21	0.32	3	1	0.33
Fatty acids	26.16	10.74	0.58	34.02	17.58	0.73	3	0	0.22
Esters	2.98	1.00	0.48	3.30	0.63	0.27	1	0	0.43
Ketones	12.31	1.89	0.22	14.97	4.53	0.43	5	0	0.00
Diverse functional groups	3.62	4.43	1.73	7.35	9.00	1.73	1	1	1.73
Phenols	1.56	1.91	1.73	1.37	1.68	1.73	0	0	1.73
TOTAL/N = 36	76.37	6.71	0.12	97.20	38.43	0.56	20	2	0.12
*Potamogeton natans*									
Alcohols	23.25	6.16	0.37	124.71	13.84	0.16	4	0	0.13
Aldehydes	1.60	0.45	0.39	8.73	1.56	0.25	1	0	0.00
Hydrocarbons	1.69	1.04	0.87	12.00	9.28	1.09	1	1	0.87
Fatty acids	10.77	6.13	0.81	88.21	79.19	1.27	2	1	0.50
Esters	25.50	4.64	0.26	159.23	67.05	0.60	5	1	0.20
unidentified	0.47	0.57	1.73	4.96	6.07	1.73	0	0	1.73
Ketones	0.49	0.61	1.73	5.26	6.45	1.73	0	0	1.73
Diverse functional groups	18.73	3.53	0.27	106.94	26.04	0.34	5	1	0.29
TOTAL/N = 31	82.51	2.65	0.05	510.05	202.36	0.56	20	1	0.11
*Potamogeton perfoliatus*									
Aromatic Hydrocarbons	0.19	0.20	2.45	0.22	0.24	2.45	0	0	2.45
Alcohols	18.51	2.81	0.34	21.59	12.73	1.32	5	0	0.22
Aldehydes	11.59	3.90	0.75	5.48	0.66	0.27	4	1	0.52
Hydrocarbons	13.45	2.81	0.47	13.24	5.89	0.99	6	1	0.34
Fatty acids	15.69	5.88	0.84	20.73	10.85	1.17	3	1	0.59
Esters	1.04	0.73	1.56	0.27	0.19	1.56	1	0	1.67
Ketones	12.73	3.73	0.66	11.85	7.07	1.33	4	1	0.61
Diverse functional groups	6.59	2.67	0.91	5.05	2.86	1.27	3	1	0.66
Phenols	0.33	0.36	2.45	0.07	0.08	2.45	0	0	2.45
TOTAL/N = 71	80.11	3.31	0.09	78.50	36.65	1.04	26	2	0.19
*Myriophyllum spicatum*									
Alcohols	12.64	5.17	0.58	33.65	7.62	0.32	2	0	0.38
Aldehydes	4.22	2.61	0.87	9.19	5.67	0.87	1	1	1.20
Hydrocarbons	23.60	4.24	0.25	86.85	46.21	0.75	5	0	0.16
Fatty acids	31.55	12.02	0.54	133.08	105.26	1.12	6	0	0.10
Esters	3.97	1.11	0.40	12.89	5.30	0.58	1	0	0.40
unidentified	0.44	0.53	1.73	1.11	1.36	1.73	0	0	2.00
Ketones	5.60	2.82	0.71	13.64	3.66	0.38	2	1	0.58
TOTAL/N = 25	82.01	2.48	0.04	290.41	141.10	0.69	17	2	0.23
*Persicaria amphibia*									
Aromatic Hydrocarbons	0.33	0.41	1.73	0.43	0.52	1.73	0	0	1.73
Alcohols	2.83	0.81	0.40	2.57	0.57	0.31	1	0	0.00
Aldehydes	6.15	0.71	0.16	6.11	1.91	0.44	2	0	0.00
Hydrocarbons	3.73	1.63	0.62	3.39	1.92	0.80	2	1	0.49
Fatty acids	52.74	3.64	0.10	56.36	21.93	0.55	6	1	0.20
Esters	0.34	0.42	1.73	0.14	0.17	1.73	0	0	1.73
unidentified	0.39	0.47	1.73	0.49	0.60	1.73	0	0	1.73
Sulfur-containing	1.11	0.74	0.95	1.20	1.17	1.38	1	0	0.87
Ketones	5.09	2.35	0.65	6.25	4.36	0.99	2	1	0.65
TOTAL/N = 21	72.71	2.78	0.05	76.93	29.06	0.53	15	1	0.07
*Nuphar lutea*									
Alcohols	3.99	2.89	0.72	239.97	294.63	1.23	1	0	0.00
Aldehydes	0.50	0.71	1.41	37.14	52.52	1.41	1	1	1.41
Hydrocarbons	2.46	3.48	1.41	182.42	257.98	1.41	1	1	1.41
Fatty acids	78.67	8.28	0.11	3388.91	2850.72	0.84	5	0	0.00
Esters	1.69	0.57	0.34	64.93	43.72	0.67	2	1	0.47
TOTAL/N = 11	87.32	1.77	0.02	3913.37	3499.58	0.89	9	1	0.16
*Ceratophyllum demersum*									
Alcohols	5.93			3.32			3		
Aldehydes	16.09			8.99			6		
Hydrocarbons	6.98	–	–	3.90	–	–	4	–	–
Fatty acids	14.62	–	–	8.17	–	–	4	–	–
Esters	7.57	–	–	4.23	–	–	2	–	–
Ketones	19.78	–	–	11.06	–	–	7	–	–
TOTAL/N = 26	70.97	–	–	39.67	–	–	26	–	–
*Lobelia dortmanna*									
Alcohols	1.34	–	–	2.66	–	–	1	–	–
Aldehydes	1.12	–	–	2.23	–	–	1	–	–
Hydrocarbons	23.96	–	–	47.63	–	–	5	–	–
Fatty acids	47.39	–	–	94.19	–	–	5	–	–
Ketones	4.75	–	–	9.45	–	–	1	–	–
TOTAL/N = 13	78.57	–	–	156.16	–	–	13	–	–

Note: %—percentage content of a group of LMWOCs from the sum of all LMWOCs; C—absolute content of a group of LMWOCs, μg·g^−1^ DW; *n*—number of major LMWOCs in a group in a specific sample; “*n*” is an integer value for each sample; where *n* is shown as 0 while CV is non-zero, this indicates that the mean value of *n* (averaged over several samples) was <0.5 and therefore rounded to 0 in the table; N—total number of major compounds for a species; Mean—arithmetic mean; SEM—standard error of the mean; CV—coefficient of variation; «–»—the calculation of the indicator is not applicable.

**Table 2:** Similarity matrix of major components based on presence/absence data: J—Jaccard similarity coefficient (lower left triangle); Qs—Sørensen–Czekanowski coefficient (upper right triangle, italics).

*J/Qs*	M.s.	S. f.	P.a.	P.per.	N.l.	S.g.	S.e.	P.pec.	P.n.	L.d.	C.d.
**M.s.**		*0.44*	*0.52*	*0.35*	*0.61*	*0.44*	*0.53*	*0.30*	*0.32*	*0.42*	*0.44*
**S. f.**	0.28		*0.30*	*0.19*	*0.39*	*0.67*	*0.68*	*0.23*	*0.29*	*0.32*	*0.32*
**P.a.**	0.35	0.18		*0.35*	*0.56*	*0.29*	*0.35*	*0.39*	*0.27*	*0.41*	*0.43*
**P.per.**	0.22	0.10	0.21		*0.24*	*0.20*	*0.21*	*0.45*	*0.25*	*0.17*	*0.40*
**N.l.**	0.44	0.24	0.39	0.14		*0.45*	*0.50*	*0.26*	*0.33*	*0.58*	*0.44*
**S.g.**	0.29	0.50	0.17	0.11	0.29		*0.73*	*0.18*	*0.31*	*0.36*	*0.31*
**S.e.**	0.36	0.52	0.21	0.12	0.33	0.57		*0.25*	*0.36*	*0.38*	*0.37*
**P.pec.**	0.18	0.13	0.24	0.29	0.15	0.10	0.14		*0.27*	*0.21*	*0.43*
**P.n.**	0.19	0.17	0.16	0.15	0.20	0.19	0.22	0.16		*0.27*	*0.32*
**L.d.**	0.27	0.19	0.26	0.09	0.41	0.22	0.24	0.12	0.16		*0.26*
**C.d.**	0.28	0.19	0.28	0.25	0.29	0.18	0.23	0.28	0.19	0.15	

Note: M.s.—*M. spicatum*, S.e.—*S. emersum*, S.g.—*S. gramineum*, S. f.—the hybrid *S. × foliosum*, P.a.—*P. amphibia*, P.per.—*P. perfoliatus*, N.l.—*N. lutea*, P.pec.—*P. pectinatus*, P.n.—*P. natans*, L.d.—*L. dortmanna*, C.d.—*C. demersum*.

**Table 3:** Similarity matrix based on concentration data of major components (Morisita–Horn index, Cmh).

Cmh	M.s.	S. f.	P.a.	P.per.	N.l.	S.g.	S.e.	P.pec.	P.n.	L.d.	C.d.
**M.s.**		0.54	0.48	0.45	0.69	0.47	0.58	0.38	0.36	0.48	0.37
**S. f.**	0.54		0.24	0.28	0.22	0.91	0.96	0.24	0.22	0.47	0.15
**P.a.**	0.48	0.24		0.43	0.81	0.29	0.37	0.64	0.59	0.90	0.48
**P.per.**	0.45	0.28	0.43		0.41	0.27	0.34	0.51	0.53	0.39	0.46
**N.l.**	0.69	0.22	0.81	0.41		0.26	0.33	0.43	0.49	0.76	0.41
**S.g.**	0.47	0.91	0.29	0.27	0.26		0.86	0.23	0.25	0.53	0.15
**S.e.**	0.58	0.96	0.37	0.34	0.33	0.86		0.31	0.31	0.56	0.20
**P.pec.**	0.38	0.24	0.64	0.51	0.43	0.23	0.31		0.54	0.53	0.58
**P.n.**	0.36	0.22	0.59	0.53	0.49	0.25	0.31	0.54		0.55	0.43
**L.d.**	0.48	0.47	0.90	0.39	0.76	0.53	0.56	0.53	0.55		0.37
**C.d.**	0.37	0.15	0.48	0.46	0.41	0.15	0.20	0.58	0.43	0.37	

**Table 4:** Top 3 most abundant major LMWOCs by relative contribution (%), with Cmax values (μg·g^−1^ DW).

Species	Top1	Top2	Top3
*Myriophyllum spicatum*	Linoleic acid 17.00% (102.45)	Phytol 16.39% (41.75)	Tricosane 13.47% (61.14)
*Sparganium* × *foliosum*	Pentacosane 39.35% (65.39)	Heptacosane 17.39% (27.49)	Palmitic acid 11.03% (16.82)
*Sparganium gramineum*	Pentacosane 31.42% (51.25)	Palmitic acid 17.17% (17.60)	Octadecyl propan-2-yl sulfite 10.37% (17.34)
*Sparganium emersum*	Pentacosane 28.66% (33.47)	Palmitic acid 15.05% (15.27)	Tricosane 13.73% (13.94)
*Persicaria amphibia*	Palmitic acid 42.53% (54.45)	α-Linolenic acid 10.43% (8.09)	Myristic acid 8.33% (12.25)
*Potamogeton perfoliatus*	α-Linolenic acid 18.41% (21.79)	Manool 16.44% (44.18)	Palmitic acid 13.55% (32.14)
*Potamogeton pectinatus*	Palmitic acid 23.92% (30.88)	Myristic acid 14.55% (22.37)	6,10,14-Trimethylpentadecan-2-one 9.09% (8.55)
*Potamogeton natans*	Manool 19.61% (74.72)	Palmitic acid 16.57% (176.50)	Methyl athecate 11.21% (90.48)
*Nuphar lutea*	α-Linolenic acid 31.31% (807.98)	Palmitic acid 29.06% (2156.62)	Linoleic acid 25.03% (1857.81)
*Lobelia dortmanna*	Palmitic acid 34.40% (68.38)	Pentacosane 11.92% (23.69)	Cyclohexadec-8-en-1-one 4.75% (9.45)
*Ceratophyllum demersum*	Palmitic acid 7.86% (4.39)	β-Ionone 6.74% (3.77)	Methyl octadecanoate 6.28% (3.51)

**Table 5:** Maximum relative contributions of the key major n-alkanes in the studied macrophyte species.

Species	Tricosane (C23)	Pentacosane (C25)	Heptacosane (C27)
*Sparganium* × *foliosum*	10.84	39.35	17.39
*Sparganium emersum*	13.73	28.66	10.95
*Sparganium gramineum*	4.24	31.42	7.56
*Myriophyllum spicatum*	13.47	13.44	2.79
*Potamogeton perfoliatus*	10.79	8.73	2.16
*Lobelia dortmanna*	1.39	11.92	–
*Potamogeton pectinatus*	7.83	2.26	–
*Potamogeton natans*	1.11	1.33	–
*Persicaria amphibia*	2.22	–	–
*Nuphar lutea*	2.59	–	–
*Ceratophyllum demersum*	1.04	–	–

Note: –: the compound is not included in the major components of the species.

**Table 6:** Locations of sampling of the studied macrophyte species.

Macrophytes	Macrophyte Growth Form	Water Bodies	Coordinates
*Myriophyllum spicatum*	submerged rooted	Shchuchiy Bay (Lake Ladoga, Russia)	61.084505, 30.093161
		Lake Uzkoe (Karelian Isthmus, Northwestern Russia)	61.125463, 29.932658; 61.120393, 29.943418
		Lake Naroch (Belarus)	54.888553, 26.742566
*Sparganium emersum*	emergent rooted; amphibious	Lake Vysokovskoe (Ivanovo Region, Russia);	57.172625, 40.940477
		Lake Zaozerye (Yaroslavl Region, Russia)	56.822257, 39.355154
*Sparganium gramineum*	submerged rooted	Lake Sabro (Tver Region, Russia)	57.158032, 32.867236
		Lake Glubokoye (Moscow Region, Russia)	55.753909, 36.504919
		Lake Chashnitsy (Yaroslavl Region, Russia)	56.942048, 39.378603
		Lake Levinskoye (Ivanovo Region, Russia)	56.772300, 42.084149
*Sparganium* × *foliosum*	submerged rooted	Lake Tikhmen (Tver Region, Russia)	57.434576, 33.470680
		Lake Sabro (Tver Region, Russia)	57.158032, 32.867236
		Lake Vysokovskoe (Ivanovo Region, Russia);	57.172625, 40.940477
		Lake Glubokoye (Moscow Region, Russia)	55.753909, 36.504919
		Lake Chashnitsy (Yaroslavl Region, Russia)	56.942048, 39.378603
		Lake Zaozerye (Yaroslavl Region, Russia)	56.822257, 39.355154
*Persicaria amphibia*	amphibious; floating-leaved rooted in aquatic form	Lake Ladoga (Russia)	61.565967, 31.464794; 61.485983, 30.230267; 61.706917, 31.000617
*Potamogeton perfoliatus*	submerged rooted	Lake Ladoga (Russia)	60.130663, 32.292573; 61.619328, 31.174475; 61.136099, 29.945312; 60.271365, 32.631411; 61.319334, 31.647898; 60.525585, 32.687798
*Potamogeton pectinatus*	submerged rooted	Volkhov Bay (Lake Ladoga, Russia)	60.132193, 32.311577
		Lake Kurnistoye (Astrakhan Region, Russia)	48.490487, 45.587603;
		Lake Obvalovannoe (Astrakhan Region, Russia)	48.488700, 45.582416
*Potamogeton natans*	floating-leaved rooted	Lake Suuri (Karelian Isthmus, Northwestern Russia)	61.132199, 29.920507
		Lake Kuznechnoye (Karelian Isthmus, Northwestern Russia)	61.117554, 29.879505
		Lake Bolshoe Borovskoye (Karelian Isthmus, Northwestern Russia)	61.102181, 29.960318
*Nuphar lutea*	floating-leaved rooted	Lake Kuznechnoye (Karelian Isthmus, Northwestern Russia)	61.118322, 29.878214; 61.112895, 29.892411
*Lobelia dortmanna*	submerged rooted	Lake Yansorskoye (Vologda region, Russia)	61.099805, 37.906015
*Ceratophyllum demersum*	submerged free-floating/rootless	Lake Makarkino (Astrakhan Region, Russia)	48.484439, 45.571462

## Data Availability

The data supporting the findings of this study are included in the article and Supplementary Materials. Raw GC–MS data (instrument raw files) and compound identification spectra/outputs are available from the corresponding author upon reasonable request.

## Supplementary Materials

The following supporting information can be downloaded at: https://www.mdpi.com/article/10.3390/molecules31050895/s1, Table S1: Component composition of major LMWOCs of investigated species (RI—retention index; %—maximum percentage content of a substance from the sum of all substances; C—maximum absolute content of substance, μg/g dry plant weight).

## Abbreviations

The following abbreviations are used in this manuscript:

AH	Aromatic hydrocarbons
BVOCs	Biogenic volatile organic compounds
C	Absolute content of a group of LMWOCs, μg·g^−1^ DW μg/g dry plant weight
Cmh	Morisita–Horn index
CV	Coefficient of variation
DBL	Diffusive boundary layer
DW	Dry plant weight
FFAs	Free fatty acids
GC–MS	Gas chromatography-mass spectrometry
GLVs	Green leaf volatiles
HAB	Harmful algal bloom
J	Jaccard similarity coefficient (lower left triangle)
LMWM	Low-molecular-weight metabolome
LMWOCs	Low-molecular-weight organic compounds
*n*	Number of major LMWOCs in a group in a specific sample
N	Total number of major compounds for a species
Qs	Sørensen–Czekanowski coefficient
QSAR	Quantitative structure-activity relationship
RI	Retention index
SEM	Standard error of the mean
VOCs	Volatile organic compounds

## References

[1] Jeppesen E., Søndergaard M., Søndergaard M., Christoffersen K. (1998). The Structuring Role of Submerged Macrophytes in Lakes.

[2] Thomaz S.M., da Cunha E.R. (2010). The role of macrophytes in habitat structuring in aquatic ecosystems: Methods of measurement, causes and consequences on animal assemblages’ composition and biodiversity. Acta Limnol. Bras..

[3] Meerhoff M., González-Sagrario M.A. (2022). Habitat complexity in shallow lakes and ponds: Importance, threats, and potential for restoration. Hydrobiologia.

[4] Scheffer M., Hosper S.H., Meijer M.L., Moss B., Jeppesen E. (1993). Alternative equilibria in shallow lakes. Trends Ecol. Evol..

[5] Phillips G., Willby N. (2016). Submerged macrophyte decline in shallow lakes: What have we learnt in the last forty years?. Aquat. Bot..

[6] Sand-Jensen K. (1980). Phytoplankton and Epiphyte Development and Their Shading Effect on Submerged Macrophytes in Lakes of Different Nutrient Status. Int. Rev. Gesamten Hydrobiol. Hydrogr..

[7] Gross E.M. (2003). Allelopathy of aquatic autotrophs. Crit. Rev. Plant Sci..

[8] Nishihara G.N., Ackerman J.D. (2009). Diffusive boundary layers do not limit the photosynthesis of the aquatic macrophyte, Vallisneria americana, at moderate flows and saturating light levels. Limnol. Oceanogr..

[9] Van Donk E., Van de Bund W.J. (2002). Impact of submerged macrophytes including charophytes on phyto- and zooplankton communities: Allelopathy versus other mechanisms. Aquat. Bot..

[10] Hilt S., Gross E.M. (2008). Can allelopathically active submerged macrophytes stabilise clear-water states in shallow eutrophic lakes?. Basic Appl. Ecol..

[11] Liu X., Sun T., Yang W., Li X., Ding J., Fu X. (2024). Meta-analysis to identify inhibition mechanisms for the effects of submerged plants on algae. J. Environ. Manag..

[12] Inderjit, Callaway R.M. (2003). Experimental designs for the study of allelopathy. Plant Soil..

[13] Gross E.M., Meyer H., Schilling G. (1996). Release and ecological impact of algicidal hydrolysable polyphenols in *Myriophyllum spicatum*. Phytochemistry.

[14] Reynolds S.A., Aldridge D.C. (2021). Embracing the allelopathic potential of invasive aquatic plants to manipulate freshwater ecosystems. Front. Environ. Sci..

[15] Erhard D., Gross E.M. (2006). Allelopathic activity of *Elodea canadensis* and *Elodea nuttallii* against epiphytes and phytoplankton. Aquat. Bot..

[16] Hilt S. (2006). Allelopathic inhibition of epiphytes by submerged macrophytes. Aquat. Bot..

[17] Otsuki A., Wetzel R.G. (1974). Release of dissolved organic matter by autolysis of a submersed macrophyte, *Scirpus subterminalis*. Limnol. Oceanogr..

[18] Wetzel R.G. (1992). Gradient-dominated ecosystems: Sources and regulatory functions of dissolved organic matter in freshwater ecosystems. Hydrobiologia.

[19] Dong B., Han R., Wang G., Cao X. (2014). O_2_, pH, and redox potential microprofiles around Potamogeton malaianus measured using microsensors. PLoS ONE.

[20] Han B., Zhang S., Wang P., Wang C. (2018). Effects of water flow on submerged macrophyte-biofilm systems in constructed wetlands. Sci. Rep..

[21] Hu S., He R., He X., Zeng J., Zhao D. (2023). Niche-Specific Restructuring of Bacterial Communities Associated with Submerged Macrophyte under Ammonium Stress. Appl. Environ. Microbiol..

[22] Shi Y., Zhang X., Zhao M., Zheng X., Gu J., Wang Z., Fan C., Gu W. (2024). The Status of Research on the Root Exudates of Submerged Plants and Their Effects on Aquatic Organisms. Water.

[23] Müller N., Hempel M., Philipp B., Gross E.M. (2007). Degradation of gallic acid and hydrolysable polyphenols is constitutively activated in the freshwater plant-associated bacterium. *Matsuebacter* sp. FB25. Aquat. Microb. Ecol..

[24] Srivastava J.K., Chandra H., Kalra S.J.S., Mishra P., Khan H., Yadav P. (2017). Plant–microbe interaction in aquatic system and their role in the management of water quality: A review. Appl. Water Sci..

[25] Fiehn O. (2002). Metabolomics—The link between genotypes and phenotypes. Plant Mol. Biol..

[26] Sumner L.W., Amberg A., Barrett D., Beale M.H., Beger R., Daykin C.A., Fan T.W., Fiehn O., Goodacre R., Griffin J.L. (2007). Proposed minimum reporting standards for chemical analysis Chemical Analysis Working Group (CAWG) Metabolomics Standards Initiative (MSI). Metabolomics.

[27] Kurashov E.A., Mitrukova G.G., Krylova Y.V. (2014). Component composition of the essential oil of *Ceratophyllum demersum* (Ceratophyllaceae) during the growing season. Plant Resour..

[28] Tang Y., Qian C., Zhao L., Wang C., Tang B., Peng X., Cheng Y., Guo X. (2023). Comparative metabolomics analysis of *Elodea nuttallii* and *Cladophora* sp. in aquaculture systems. Aquaculture.

[29] Krylova J.V., Kurashov E.A., Protopopova E.V., Khodonovich V.V., Yavid E.Y., Kuchareva G.I. (2024). Composition of the low molecular weight metabolome of *Potamogeton perfoliatus* (Potamogetonaceae) as an indicator of the transformation of the ecological state of the littoral zone. Inland Water Biol..

[30] Sarkar A., Roy S. (2024). Metabolome profile variation in Azolla filiculoides exposed to Bisphenol A assists in the identification of stress-responsive metabolites. Aquat. Toxicol..

[31] Yang X., Chen H., Wu L., Guo X., Xue D. (2025). Diversity and correlation analysis of microbiomes and metabolites of Sphagnum palustre in various microhabitats. BMC Plant Biol..

[32] Hempel M., Blume M., Blindow I., Gross E.M. (2008). Epiphytic bacterial community composition on two common submerged macrophytes in brackish water and freshwater. BMC Microbiol..

[33] Sun L., Wang J., Wu Y., Gao T., Liu C. (2021). Community Structure and Function of Epiphytic Bacteria Associated with *Myriophyllum spicatum* in Baiyangdian Lake, China. Front. Microbiol..

[34] Zhang T., Bao F., Yang Y., Hu L., Ding A., Ding A., Wang J., Cheng T., Zhang Q.A. (2019). Comparative Analysis of Floral Scent Compounds in Intraspecific Cultivars of Prunus mume with Different Corolla Colours. Molecules.

[35] Choo K.S.O., Bollen M., Dykes G.A., Coorey R. (2021). Aroma-volatile profile and its changes in Australian grown black Périgord truffle (Tuber melanosporum) during storage. Int. J. Food Sci. Technol..

[36] Miastkowska M., Kantyka T., Bielecka E., Kałucka U., Kamińska M., Kucharska M., Kilanowicz A., Cudzik D., Cudzik K. (2021). Enhanced Biological Activity of a Novel Preparation of *Lavandula angustifolia* Essential Oil. Molecules.

[37] Mickle A.M., Wetzel R.G. (1978). Effectiveness of submersed angiosperm-epiphyte complexes on exchange of nutrients and organic carbon in littoral systems. II. Dissolved organic carbon. Aquat. Bot..

[38] Inderjit, Weston L.A. (2000). Are Laboratory Bioassays for Allelopathy Suitable for Prediction of Field Responses?. J. Chem. Ecol..

[39] Hickman D.T., Comont D., Rasmussen A., Birkett M.A. (2023). Novel and holistic approaches are required to realize allelopathic potential for weed management. Ecol. Evol..

[40] Leu E., Krieger-Liszkay A., Goussias C., Gross E.M. (2002). Polyphenolic allelochemicals from the aquatic angiosperm *Myriophyllum spicatum* inhibit photosystem II. Plant Physiol..

[41] Ianora A., Bentley M.G., Caldwell G.S., Casotti R., Cembella A.D., Engström-Öst J., Halsband C., Sonnenschein E., Legrand C., Llewellyn C.A. (2011). The Relevance of Marine Chemical Ecology to Plankton and Ecosystem Function: An Emerging Field. Mar. Drugs.

[42] Soto-Cruz F.J., Zorrilla J.G., Rial C., Varela R.M., Molinillo J.M.G., Igartuburu J.M., Macías F.A. (2021). Allelopathic Activity of Strigolactones on the Germination of Parasitic Plants and Arbuscular Mycorrhizal Fungi Growth. Agronomy.

[43] Yu Y., Li F., Belyakov E.A., Yang W., Lapirov A.G., Xu X. (2022). Molecular confirmation of the hybrid origin of *Sparganium longifolium* (Typhaceae). Sci. Rep..

[44] Belyakov E.A., Mikhaylova Y.V., Machs E.M., Zhurbenko P.M., Rodionov A.V. (2022). Hybridization and diversity of aquatic macrophyte *Sparganium* L. (Typhaceae) as revealed by high-throughput nrDNA sequencing. Sci. Rep..

[45] Bobrov A.A., Volkova P.A., Mochalova O.A., Chemeris E.V. (2023). High diversity of aquatic *Sparganium* (Xanthosparganium, Typhaceae) in North Eurasia is mostly explained by recurrent hybridization. Perspect. Plant Ecol. Evol. Syst..

[46] Dan Z., Chen Y., Zhao W., Wang Q., Huang W. (2019). Metabolome-based prediction of yield heterosis contributes to the breeding of elite rice. Life Sci. Alliance.

[47] Li Z., Zhu A., Song Q., Chen H.Y., Harmon F.G., Chen Z.J. (2020). Temporal Regulation of the Metabolome and Proteome in Photosynthetic and Photorespiratory Pathways Contributes to Maize Heterosis. Plant Cell.

[48] Porretta D., Canestrelli D. (2023). The ecological importance of hybridization. Trends Ecol. Evol..

[49] Dan Z., Chen Y., Li H., Zeng Y., Xu W., Zhao W., He R., Huang W. (2021). The metabolomic landscape of rice heterosis highlights pathway biomarkers for predicting complex phenotypes. Plant Physiol..

[50] Le Q.T.N., Sugi N., Yamaguchi M., Hirayama T., Kobayashi M., Suzuki Y., Kusano M., Shiba H. (2023). Morphological and metabolomics profiling of intraspecific *Arabidopsis* hybrids in relation to biomass heterosis. Sci. Rep..

[51] Kirk H., Cheng D., Choi Y.H., Vrieling K., Klinkhamer P.G. (2012). Transgressive segregation of primary and secondary metabolites in F(2) hybrids between *Jacobaea aquatica* and *J. vulgaris*. Metabolomics Off. J. Metabolomic Soc..

[52] Kunst L., Samuels A.L. (2003). Biosynthesis and secretion of plant cuticular wax. Prog. Lipid Res..

[53] Samuels L., Kunst L., Jetter R. (2008). Sealing plant surfaces: Cuticular wax formation by epidermal cells. Annu. Rev. Plant Biol..

[54] Körner S., Nicklisch A. (2002). Allelopathic growth inhibition of selected phytoplankton species by submerged macrophytes. J. Phycol..

[55] Sculthorpe C.D. (1967). The Biology of Aquatic Vascular Plants.

[56] DellaGreca M., Fiorentino A., Isidori M., Monaco P., Temussi F., Zarrelli A. (2001). Antialgal furano-diterpenes from *Potamogeton natans* L.. Phytochemistry.

[57] Sand-Jensen K. (1989). Environmental variables and their effect on photosynthesis of aquatic plant communities. Aquat. Bot..

[58] Guschina I.A., Harwood J.L. (2006). Lipids and lipid metabolism in eukaryotic algae. Prog. Lipid Res..

[59] Arts M.T., Brett M.T., Kainz M.J. (2009). Lipids in Aquatic Ecosystems.

[60] Mohamed Z.A. (2017). Macrophytes-cyanobacteria allelopathic interactions and their implications for water resources management—A review. Limnologica.

[61] Cui Z., Yang C., Ma L., Gu X., Shen X., Wan B., Tao Y., Sang Y., Huang Q. (2025). Floating-leaved and submerged macrophytes suppress filamentous cyanobacteria blooms and 2-MIB episodes in eutrophic shallow lakes. J. Hazard. Mater..

[62] Zhang S.H., Sun P.S., Ge F.J., Wu Z.-B. (2011). Different Sensitivities of *Selenastrum capricornutum* and Toxic Strain *Microcystis aeruginosa* to Exudates from Two *Potamogeton* Species. Pol. J. Environ. Stud..

[63] Asaeda T., Sultana M., Manatunge J., Fujino T. (2004). The effect of epiphytic algae on the growth and production of *Potamogeton perfoliatus* L. in two light conditions. Environ. Exp. Bot..

[64] Tóth V.R. (2025). The impact of epiphytic algae on the foliar traits of *Potamogeton perfoliatus*. Front. Plant Sci..

[65] Nakai S., Yamada S., Hosomi M. (2005). Anti-cyanobacterial fatty acids released from *Myriophyllum spicatum*. Hydrobiologia.

[66] Nakai S., Zou G., Okuda T., Nishijima W., Hosomi M., Okada M. (2012). Polyphenols and fatty acids responsible for anti-cyanobacterial allelopathic effects of submerged macrophyte. *Myriophyllum spicatum*. Water Sci. Technol..

[67] Matsui K. (2006). Green leaf volatiles: Hydroperoxide lyase pathway of oxylipin metabolism. Curr. Opin. Plant Biol..

[68] Holopainen J.K., Gershenzon J. (2010). Multiple stress factors and the emission of plant VOCs. Trends Plant Sci..

[69] Nakai S., Inoue Y., Hosomi M., Murakami A. (2000). *Myriophyllum spicatum*-released allelopathic polyphenols inhibiting growth of blue-green algae *Microcystis aeruginosa*. Water Res..

[70] Casillas-Vargas G., Ocasio-Malavé C., Medina S., Morales-Guzmán C., Del Valle R.G., Carballeira N.M., Sanabria-Ríos D.J. (2021). Antibacterial fatty acids: An update of possible mechanisms of action and implications in the development of the next-generation of antibacterial agents. Prog. Lipid Res..

[71] Nezbrytska I., Usenko O., Konovets I., Leontieva T., Abramiuk I., Goncharova M., Bilous O. (2022). Potential use of aquatic vascular plants to control cyanobacterial blooms: A review. Water.

[72] Wasternack C., Feussner I. (2018). The Oxylipin Pathways: Biochemistry and Function. Annu. Rev. Plant Biol..

[73] Scala A., Allmann S., Mirabella R., Haring M.A., Schuurink R.C. (2013). Green leaf volatiles: A plant’s multifunctional weapon against herbivores and pathogens. Int. J. Mol. Sci..

[74] Xue D., Zhang X., Lu X., Chen G., Chen Z.H. (2017). Molecular and Evolutionary Mechanisms of Cuticular Wax for Plant Drought Tolerance. Front. Plant Sci..

[75] Jetter R., Kunst L. (2008). Plant surface lipid biosynthetic pathways and their utility for metabolic engineering of waxes and hydrocarbon biofuels. Plant J..

[76] Partridge J.W. (2001). *Persicaria amphibia* (L.) Gray (*Polygonum amphibium* L.). J. Ecol..

[77] Ossola R., Farmer D. (2024). The Chemical Landscape of Leaf Surfaces and Its Interaction with the Atmosphere. Chem. Rev..

[78] Anikina V.V., Yavid E.Y., Krylova J.V., Kurashov E.A., Grebennikov V.A., Protopopova E.V., Bash P.V. (2025). Interannual Variability of Low-Molecular-Weight Metabolome of *Nuphar lutea* (L.) Sm. (Nymphaeaceae) in Lake Kuznechnoye (Leningrad Oblast) in Different Trophic States. Contemp. Probl. Ecol..

[79] Blée E. (2002). Impact of phyto-oxylipins in plant defense. Trends Plant Sci..

[80] Kurashov E.A., Krylova J.V., Mitrukova G.G., Chernova A.M. (2014). Low-molecular-weight metabolites of aquatic macrophytes growing on the territory of Russia and their role in hydroecosystems. Contemp. Probl. Ecol..

[81] Kurashov E.A., Krylova J.V., Protopopova E.V., Pereira L., Gonçalves A.M. (2021). The use of allelochemicals of aquatic macrophytes to suppress the development of cyanobacterial blooms. Plankton Communities.

[82] Wium-Andersen S. (1971). Photosynthetic uptake of free CO_2_ by the roots of *Lobelia dortmanna*. Physiol. Plant..

[83] Sand-Jensen K., Pedersen O., Binzer T., Borum J. (2005). Contrasting oxygen dynamics in the freshwater isoetid *Lobelia dortmanna* and the marine seagrass *Zostera marina*. Ann. Bot..

[84] Wolda H. (1981). Similarity indices, sample size and diversity. Oecologia.

[85] Wink M. (2003). Evolution of secondary metabolites from an ecological and molecular phylogenetic perspective. Phytochemistry.

[86] Orians C.M. (2000). The effects of hybridization in plants on secondary chemistry: Implications for the ecology and evolution of plant-herbivore interactions. Am. J. Bot..

[87] Kirk H., Choi Y.H., Kim H.K., Verpoorte R., van der Meijden E. (2005). Comparing metabolomes: The chemical consequences of hybridization in plants. New Phytol..

[88] Cheng D., Vrieling K., Klinkhamer P.G. (2011). The effect of hybridization on secondary metabolites and herbivore resistance: Implications for the evolution of chemical diversity in plants. Phytochem. Rev. Proc. Phytochem. Soc. Eur..

[89] Isidorov V.A., Stocki M., Vetchinikova L. (2019). Inheritance of specific secondary volatile metabolites in buds of white birch *Betula pendula* and *Betula pubescens* hybrids. Trees.

[90] Jouhet J., Alves E., Boutté Y., Darnet S., Domergue F., Durand T., Fischer P., Fouillen L., Grube M., Joubès J. (2024). Plant and algal lipidomes: Analysis, composition, and their societal significance. Prog. Lipid Res..

[91] Kaplan Z. (2002). Phenotypic plasticity in *Potamogeton* (Potamogetonaceae). Folia Geobot..

[92] Arnold T., Mealey C., Leahey H., Miller A.W., Hall-Spencer J.M., Milazzo M., Maers K. (2012). Ocean acidification and the loss of phenolic substances in marine plants. PLoS ONE.

[93] Wium-Andersen S., Anthoni U., Houen G. (1983). Elemental sulphur, a possible allelopathic compound from *Ceratophyllum demersum*. Phytochemistry.

[94] Gross E.M., Erhard D., Iványi E. (2003). Allelopathic activity of *Ceratophyllum demersum* L. and *Najas marina* ssp. *intermedia* (Wolfgang) Casper. Hydrobiologia.

[95] Kurashov E.A., Mitrukova G.G., Krylova Y.V. (2018). Interannual Variability of Low-Molecular Metabolite Composition in *Ceratophyllum demersum* (Ceratophyllaceae) from a Floodplain Lake with a Changeable Trophic Status. Contemp. Probl. Ecol..

[96] Lim G.H., Singhal R., Kachroo A., Kachroo P. (2017). Fatty Acid- and Lipid-Mediated Signaling in Plant Defense. Annu. Rev. Phytopathol..

[97] Krylova J., Kurashov E. (2023). Characteristics of the Low Molecular Weight Metabolome of *Potamogeton natans* L. (Potamogetonaceae) from Lakes of Different Trophic State (Karelian Isthmus, Northwest Russia). Glob. J. Bot. Sci..

[98] Wu J.T., Chiang Y.R., Huang W.Y., Jane W.N. (2006). Cytotoxic effects of free fatty acids on phytoplankton algae and cyanobacteria. Aquat. Toxicol..

[99] Krylova Y.V., Kurashov E.A., Protopopova E.V., Khodonovich V.V., Iavid E.Y. (2023). Saturated and unsaturated fatty acids as potential allelochemicals for aquatic ecosystems rehabilitation [Nasyshchennyye i nenasyshchennyye zhirnyye kisloty kak potentsial’nyye allelokhemiki dlya reabilitatsii vodnykh ekosistem]. Ecosyst. Transform..

[100] Desbois A.P., Smith V.J. (2010). Antibacterial free fatty acids: Activities, mechanisms of action and biotechnological potential. Appl. Microbiol. Biotechnol..

[101] Kurashov E.A., Fedorova E.V., Krylova J.V., Kapustina L.L., Mitrukova G.G., Protopopova E.V. (2023). Using SAR Methodology for Identification of Freshwater Macrophyte Allelochemicals with High Anti-Cyanobacterial Effect against Planktonic Cyanobacteria. J. Sib. Fed. Univ. Biol..

[102] Rojo C., Segura M., Rodrigo M.A. (2013). The allelopathic capacity of submerged macrophytes shapes the microalgal assemblages from a recently restored coastal wetland. Ecol. Eng..

[103] Gao Y., Liu B., Ge F.J., He Y., Lu Z., Zhou Q., Zhang Y.Y., Wu Z.B. (2015). Joint effects of allelochemical nonanoic acid, N-phenyl-1-naphtylamine and caffeic acid on the growth of *Microcystis aeruginosa*. Allelopath. J..

[104] Zuo S., Zhou S., Ye L., Ma S. (2016). Synergistic and antagonistic interactions among five allelochemicals with antialgal effects on bloom-forming. Microcystis aeruginosa. Ecol. Eng..

[105] Aliotta G., Greca M.D., Monaco P., Pinto G., Pollio A., Previtera L. (1990). In vitro algal growth inhibition by phytotoxins of *Typha latifolia* L.. J. Chem. Ecol..

[106] Aliotta G., Monaco P., Pinto G., Pollio A., Previtera L. (1991). Potential allelochemicals from *Pistia stratiotes* L.. J. Chem. Ecol..

[107] Nakai S., Zhou S., Hosomi M., Tominaga M. (2006). Allelopathic growth inhibition of cyanobacteria by reed. Allelopath. J..

[108] Ni L., Jie X., Wang P., Li S., Hu S., Li Y., Li Y., Acharya K. (2015). Characterization of unsaturated fatty acid sustained-release microspheres for long-term algal inhibition. Chemosphere.

[109] Ni L., Jie X., Wang P., Li S., Wang G., Li Y., Li Y., Acharya K. (2015). Effect of linoleic acid sustained-release microspheres on *Microcystis aeruginosa* antioxidant enzymes activity and microcystins production and release. Chemosphere.

[110] Wang H.Q., Zhu H.J., Zhang L.Y., Xue W.J., Yuan B. (2014). Identification of antialgal compounds from the aquatic plant *Elodea nuttallii*. Allelopath. J..

[111] Song H., Lavoie M., Fan X., Tan H., Liu G., Xu P., Fu Z., Paerl H.W., Qian H. (2017). Allelopathic interactions of linoleic acid and nitric oxide increase the competitive ability of *Microcystis aeruginosa*. ISME J..

[112] Kurashov E., Kapustina L., Krylova J., Mitrukova G., Grigoryeva N. (2020). The use of fluorescence microscopy to assess the suppression of the development of cyanobacteria under the influence of allelochemicals of aquatic macrophytes. Fluorescence Methods for Investigation of Living Cells and Microorganisms.

[113] Borisjuk N., Peterson A.A., Lv J., Qu G., Luo Q., Shi L., Chen G., Kishchenko O., Zhou Y., Shi J. (2018). Structural and Biochemical Properties of Duckweed Surface Cuticle. Front. Chem..

[114] Ensikat H.J., Ditsche-Kuru P., Neinhuis C., Barthlott W. (2011). Superhydrophobicity in perfection: The outstanding properties of the lotus leaf. Beilstein J. Nanotechnol..

[115] Vom Dorp K., Hölzl G., Plohmann C., Eisenhut M., Abraham M., Weber A.P., Hanson A.D., Dörmann P. (2015). Remobilization of Phytol from Chlorophyll Degradation Is Essential for Tocopherol Synthesis and Growth of Arabidopsis. Plant Cell.

[116] Gutbrod K., Romer J., Dörmann P. (2019). Phytol metabolism in plants. Prog. Lipid Res..

[117] Bisio A., Schito A.M., Pedrelli F., Danton O., Reinhardt J.K., Poli G., Tuccinardi T., Bürgi T., De Riccardis F., Giacomini M. (2020). Antibacterial and ATP Synthesis Modulating Compounds from *Salvia tingitana*. J. Nat. Prod..

[118] Iwan Jones J., Eaton J.W., Hardwick K. (2000). The influence of periphyton on boundary layer conditions: A pH microelectrode investigation. Aquat. Bot..

[119] Hempel M., Grossart H., Gross E.M. (2009). Community composition of bacterial biofilms on two submerged macrophytes; an artificial substrate in a pre-alpine lake. Aquat. Microb. Ecol..

[120] Gershenzon J., Dudareva N. (2007). The function of terpene natural products in the natural world. Nat. Chem. Biol..

[121] Fonseca A.P., Estrela F.T., Moraes T.S., Carneiro L.J., Bastos J.K., Santos R.A., Ambrósio S.R., Martins C.H.G., Veneziani R.C.S. (2013). In Vitro Antimicrobial Activity of Plant-Derived Diterpenes against Bovine Mastitis Bacteria. Molecules.

[122] Mendes F.S.F., Garcia L.M., Moraes T.D.S., Casemiro L.A., Alcântara C.B., Ambrósio S.R., Veneziani R.C.S., Miranda M.L.D., Martins C.H.G. (2020). Antibacterial activity of *Salvia officinalis* L. against periodontopathogens: An in vitro study. Anaerobe.

[123] Iobbi V., Brun P., Bernabé G., Dougué Kentsop R.A., Donadio G., Ruffoni B., Fossa P., Bisio A., De Tommasi N. (2021). Labdane Diterpenoids from *Salvia tingitana* Etl. Synergize with Clindamycin against Methicillin-Resistant *Staphylococcus aureus*. Molecules.

[124] DellaGreca M., Fiorentino A., Isidori M., Monaco P., Zarrelli A. (2000). Antialgal ent-labdane diterpenes from *Ruppia maritima*. Phytochemistry.

[125] Cangiano T., Dellagreca M., Fiorentino A., Isidori M., Monaco P., Zarrelli A. (2002). Effect of ent-labdane diterpenes from Potamogetonaceae on *Selenastrum capricornutum* and other aquatic organisms. J. Chem. Ecol..

[126] Pejin B., Savic A., Sokovic M., Glamoclija J., Ciric A., Nikolic M., Radotic K., Mojovic M. (2014). Further in vitro evaluation of antiradical and antimicrobial activities of phytol. Nat. Prod. Res..

[127] Lee W., Woo E.R., Lee D.G. (2016). Phytol has antibacterial property by inducing oxidative stress response in *Pseudomonas aeruginosa*. Free Radic. Res..

[128] Vencl F., Morton T. (1998). The shield defense of the sumac flea beetle, *Blepharida rhois* (Chrysomelidae: Alticinae). Chemoecology.

[129] Kurashov E.A., Krylova J.V., Mitrukova G.G., Chernova A.M., Biscarini C., Pierleoni A., Naselli-Flores L. (2014). The regularities of synthesis of low-molecular weight organic compounds by water macrophytes depending on biotic and abiotic factors. Lakes: The Mirrors of the Earth. Balancing Ecosystem Integrity and Human Wellbeing, Proceedings of the 15th World Lake Conference, Perugia, Italy, 1–5 September 2014.

[130] Smirnov N.N. (1960). Nutrition of *Galerucella nymphaeae* L. (Chrysomelidae), mass consumer of water-lily. Hydrobiologia.

[131] Rowland O., Domergue F. (2012). Plant fatty acyl reductases: Enzymes generating fatty alcohols for protective layers with potential for industrial applications. Plant Sci. Int. J. Exp. Plant Biol..

[132] Bernard A., Joubès J. (2013). Arabidopsis cuticular waxes: Advances in synthesis, export and regulation. Prog. Lipid Res..

[133] Lakshmi S.A., Bhaskar J.P., Krishnan V., Sethupathy S., Pandipriya S., Aruni W., Pandian S.K. (2020). Inhibition of biofilm and biofilm-associated virulence factor production in methicillin-resistant *Staphylococcus aureus* by docosanol. J. Biotechnol..

[134] Shah J. (2009). Plants under attack: Systemic signals in defence. Curr. Opin. Plant Biol..

[135] Meents A.K., Mithöfer A. (2020). Plant-Plant Communication: Is There a Role for Volatile Damage-Associated Molecular Patterns? *Front*. Plant Sci..

[136] Matsui K., Engelberth J. (2022). Green Leaf Volatiles—The Forefront of Plant Responses Against Biotic Attack. Plant Cell Physiol..

[137] Kant M.R., Bleeker P.M., Van Wijk M., Schuurink R.C., Haring M.A. (2009). Plant Volatiles in Defence. Adv. Bot. Res..

[138] Zhao Q., Zhou A., He Y. (2024). Quantification of allochthonous and autochthonous organic carbon in large and shallow Lake Wuliangsu based on distribution patterns and δ13C signatures of n-alkanes. Org. Geochem..

[139] Jetter R., Kunst L., Samuels A.L., Riederer M., Müller C. (2006). Composition of plant cuticular waxes. Biology of the Plant Cuticle.

[140] Koch K., Ensikat H.J. (2008). The hydrophobic coatings of plant surfaces: Epicuticular wax crystals and their morphologies, crystallinity and molecular self-assembly. Micron.

[141] Goldsborough L.G., Hickman M. (1991). A comparison of periphytic algal biomass and community structure on *Scirpus validus* and on a morphologically similar artificial substratum. J. Phycol..

[142] Reisberg E.E., Hildebrandt U., Riederer M., Hentschel U. (2013). Distinct phyllosphere bacterial communities on *Arabidopsis* wax mutant leaves. PLoS ONE.

[143] Schoelynck J., Papierowska E., Sikorska D., Szatyłowicz J. (2024). How wet are water plants? Determination of macrophyte leaf water repellency. Ecohydrol. Hydrobiol..

[144] Ficken K., Li B., Swain D., Eglinton G. (2000). An n-alkane proxy for the sedimentary input of submerged/floating freshwater aquatic macrophytes. Org. Geochem..

[145] Nakamura S., Hatanaka A. (2002). Green-leaf-derived C6-aroma compounds with potent antibacterial action that act on both Gram-negative and Gram-positive bacteria. J. Agric. Food Chem..

[146] Trombetta D., Saija A., Bisignano G., Arena S., Caruso S., Mazzanti G., Uccella N., Castelli F. (2002). Study on the mechanisms of the antibacterial action of some plant alpha,beta-unsaturated aldehydes. Lett. Appl. Microbiol..

[147] Zhang J.H., Sun H.L., Chen S.Y., Zeng L., Wang T.T. (2017). Anti-fungal activity, mechanism studies on α-Phellandrene and Nonanal against *Penicillium cyclopium*. Bot. Stud..

[148] Ricciardelli A., Casillo A., Corsaro M.M., Tutino M.L., Parrilli E., van der Mei H.C. (2020). Pentadecanal and pentadecanoic acid coatings reduce biofilm formation of *Staphylococcus epidermidis* on PDMS. Pathog. Dis..

[149] Friedman M., Henika P.R., Mandrell R.E. (2003). Antibacterial activities of phenolic benzaldehydes and benzoic acids against *Campylobacter jejuni*, *Escherichia coli*, *Listeria monocytogenes*, and *Salmonella enterica*. J. Food Prot..

[150] Murray D., Jefferson B., Jarvis P., Parsons S.A. (2010). Inhibition of three algae species using chemicals released from barley straw. Environ. Technol..

[151] Pozzer A.C., Gómez P.A., Weiss J. (2022). Volatile organic compounds in aquatic ecosystems—Detection, origin, significance and applications. Sci. Total Environ..

[152] Portilla K., Velarde E., Decaestecker E., Teixeira de Mello F., Muylaert K. (2023). Potential Submerged Macrophytes to Mitigate Eutrophication in a High-Elevation Tropical Shallow Lake—A Mesocosm Experiment in the Andes. Water.

[153] Peng Q., Yang Y., Ou W., Wei L., Li Z., Deng X., Gao Q. (2024). The characteristics and environmental significance of BVOCs released by aquatic macrophytes. Chemosphere.

[154] Wang L., Li Y., Zhang P., Zhang S., Li P., Wang P., Wang C. (2019). Sorption removal of phthalate esters and bisphenols to biofilms from urban river: From macroscopic to microcosmic investigation. Water Res..

[155] Balakrishnan J., Ganapathi P., Kannan S., Marudhamuthu M., Shanmugam K. (2021). Anti-listerial activity of microalgal fatty acid methyl esters and their possible applications as chicken marinade. Int. J. Food Microbiol..

[156] Vijay K., Kiran G.S., Divya S., Thangavel K., Thangavelu S., Dhandapani R., Selvin J. (2021). Fatty Acid Methyl Esters From the Coral-Associated Bacterium *Pseudomonas aeruginosa* Inhibit Virulence and Biofilm Phenotypes in Multidrug Resistant *Staphylococcus aureus*: An in vitro Approach. Front. Microbiol..

[157] Chandrasekaran M., Kannathasan K., Venkatesalu V. (2008). Antimicrobial activity of fatty acid methyl esters of some members of Chenopodiaceae. Z. Naturforschung. C J. Biosci..

[158] Davoodbasha M., Edachery B., Nooruddin T., Lee S.Y., Kim J.W. (2018). An evidence of C16 fatty acid methyl esters extracted from microalga for effective antimicrobial and antioxidant property. Microb. Pathog..

[159] Zou C., Li Z., Yu D. (2010). Bacillus megaterium strain XTBG34 promotes plant growth by producing 2-pentylfuran. J. Microbiol..

[160] Zuo Z. (2023). Emission of cyanobacterial volatile organic compounds and their roles in blooms. Front. Microbiol..

[161] Shao J., Xu Y., Wang Z., Jiang Y., Yu G., Peng X., Li R. (2011). Elucidating the toxicity targets of β-ionone on photosynthetic system of *Microcystis aeruginosa* NIES-843 (Cyanobacteria). Aquat. Toxicol..

[162] Aloum L., Alefishat E., Adem A., Petroianu G. (2020). Ionone Is More than a Violet’s Fragrance: A Review. Molecules.

[163] Paparella A., Shaltiel-Harpaza L., Ibdah M. (2021). β-Ionone: Its Occurrence and Biological Function and Metabolic Engineering. Plants.

[164] Pan N., Xu H., Chen W., Liu Z., Liu Y., Huang T., Du S., Xu S., Zheng T., Zuo Z. (2024). Cyanobacterial VOCs β-ionone and β-cyclocitral poisoning *Lemna turionifera* by triggering programmed cell death. Environ. Pollut..

[165] Jüttner F. (1979). Nor-carotenoids as the major volatile excretion products of Cyanidium. Z. Naturforsch. (Sect. C).

[166] DellaGreca M., Di Marino C., Zarrelli A., D’Abrosca B. (2004). Isolation and Phytotoxicity of Apocarotenoids from *Chenopodium album*. J. Nat. Prod..

[167] Schulz S., Yildizhan S., van Loon J.J. (2011). The biosynthesis of hexahydrofarnesylacetone in the butterfly *Pieris brassicae*. J. Chem. Ecol..

[168] Tóth P., Undas A.K., Verstappen F., Bouwmeester H. (2016). Floral Volatiles in Parasitic Plants of the Orobanchaceae. Ecological and Taxonomic Implications. Front. Plant Sci..

[169] Cakir A., Kordali S., Kilic H., Kaya E. (2005). Antifungal properties of essential oil and crude extracts of *Hypericum linarioides* Bosse. Biochem. Syst. Ecol..

[170] Radulović N., Stojanović G., Palić R. (2006). Composition and antimicrobial activity of *Equisetum arvense* L. essential oil. Phytother. Res..

[171] Kalinová B., Kindl J., Jiros P., Zácek P., Vasícková S., Budesínský M., Valterová I. (2009). Composition and electrophysiological activity of constituents identified in male wing gland secretion of the bumblebee parasite *Aphomia sociella*. J. Nat. Prod..

[172] Rontani J., Giral P., Baillet G., Raphel D. (1992). “Bound” 6,10,14-trimethylpentadecan-2-one: A useful marker for photodegradation of chlorophylls with a phytol ester group in seawater. Org. Geochem..

[173] Yang S.X., Sun J.Z., Yang J., Yuan Y., Yang Y., Qin J.C., Kuang Y., Sampietro D.A. (2020). Herbicidal, fumigant and insecticidal potential of essential oil from flowers of *Buddleja alternifolia* Maxim. Allelopath. J..

[174] Formisano C., Rigano D., Senatore F., De Feo V., Bruno M., Rosselli S. (2007). Composition and allelopathic effect of essential oils of two thistles: *Cirsium creticum* (Lam.) D’Urv. ssp. triumfetti (Lacaita). Werner and *Carduus nutans* L.. J. Plant Interact..

[175] Fawaz E.Y., Allan S.A., Bernier U.R., Obenauer P.J., Diclaro J.W. (2014). Swarming mechanisms in the yellow fever mosquito: Aggregation pheromones are involved in the mating behavior of *Aedes aegypti*. J. Vector Ecol..

[176] Zhuang X., Klingeman W.E., Hu J., Chen F. (2008). Emission of volatile chemicals from flowering dogwood (*Cornus florida* L.) flowers. J. Agric. Food Chem..

[177] Kurashov E.A., Krylova Y.V., Mitrukova G.G. (2012). Component composition of volatile low-molecular organic substances of *Ceratophyllum demersum* L. during fruiting. Water Chem. Ecol..

[178] Moran L., Bou G., Aldai N., Ciardi M., Morillas-España A., Sánchez-Zurano A., Barron L.J.R., Lafarga T. (2022). Characterisation of the volatile profile of microalgae and cyanobacteria using solid-phase microextraction followed by gas chromatography coupled to mass spectrometry. Sci. Rep..

[179] Koteska D., Sanchez Garcia S., Wagner-Döbler I., Schulz S. (2022). Identification of Volatiles of the Dinoflagellate Prorocentrum cordatum. Mar. Drugs.

[180] Kurashov E.A., Bataeva Y.V., Krylova J.V., Dyatlov I.A. (2023). Gas Chromatography-Mass Spectrometric Study of Low-Molecular-Weight Exogenous Metabolites of Algae-Bacterial Communities in the Laboratory Accumulative Culture. Water.

[181] Balderrama N., Nunez J., Guerrieri F., Giurfa M. (2002). Different functions of two alarm substances in the honeybee. J. Comp. Physiol. A.

[182] Nylund G.M., Cervin G., Persson F., Hermansson M., Steinberg P.D., Pavia H. (2008). Seaweed defence against bacteria: A poly-brominated 2-heptanone from the red alga *Bonnemaisonia hamifera* inhibits bacterial colonization. Mar. Ecol. Prog. Ser..

[183] Nylund G.M., Persson F., Lindegarth M., Cervin G., Hermansson M., Pavia H. (2010). The red alga *Bonnemaisonia asparagoides* regulates epiphytic bacterial abundance and community composition by chemical defence. FEMS Microbiol. Ecol..

[184] Höckelmann C., Moens T., Jüttner F. (2004). Odor compounds from cyanobacterial biofilms acting as attractants and repellents for free-living nematodes. Limnol. Oceanogr..

[185] Chiang Y.R., Wei S.T., Wang P.H., Wu P.H., Yu C.P. (2020). Microbial degradation of steroid sex hormones: Implications for environmental and ecological studies. Microb. Biotechnol..

[186] Shiko G., Paulmann M.J., Feistel F., Ntefidou M., Hermann-Ene V., Vetter W., Kost B., Kunert G., Zedler J.A.Z., Reichelt M. (2023). Occurrence and conversion of progestogens and androgens are conserved in land plants. New Phytol..

[187] Zahed M.A., Pardakhti A., Mohajeri L., Bateni F. (2010). Wet deposition of hydrocarbons in the city of Tehran-Iran. Air Qual. Atmos. Health.

[188] Limmer M., Burken J.G. (2016). Phytovolatilization of Organic Contaminants. Environ. Sci. Technol..

[189] Duan W., Meng F., Wang F., Liu Q. (2017). Environmental behavior and eco-toxicity of xylene in aquatic environments: A review. Ecotoxicol. Environ. Saf..

[190] Fan S., Liu H., Zheng G., Wang Y., Wang S., Liu Y., Liu X., Wan Y. (2018). Differences in phytoaccumulation of organic pollutants in freshwater submerged and emergent plants. Environ. Pollut..

[191] Misztal P.K., Hewitt C.N., Wildt J., Blande J.D., Eller A.S., Fares S., Gentner D.R., Gilman J.B., Graus M., Greenberg J. (2015). Atmospheric benzenoid emissions from plants rival those from fossil fuels. Sci. Rep..

[192] Herman D.C., Inniss W.E., Mayfield C.I. (1990). Impact of volatile aromatic hydrocarbons, alone and in combination, on growth of the freshwater alga *Selenastrum capricornutum*. Aquat. Toxicol..

[193] Wang J., Xu S., Wu N., Xu Q., Liu X. (2023). Detection of retene in Proterozoic strata: A new understanding of its environmental indicator significance. Res. Sq..

[194] Zakrzewski A., Kosakowski P., Kowalski T. (2024). Terrigenous or not? δ13C reveals the origin of the retene and dehydroabietic acid methyl ester. Chem. Geol..

[195] Ramdahl T. (1983). Retene—A molecular marker of wood combustion in ambient air. Nature.

[196] Kurashov E.A. (2011). Litoral Zone of Lake Ladoga.

[197] Hirotani M., Furuya T. (1975). Metabolism of 5β-pregnane-3,20-dione and 3β-hydroxy-5β-pregnan-20-one by Digitalis suspension cultures. Phytochemistry.

[198] Lin J.T., Heftmann E. (1981). Stereospecific reduction of progesterone by *Pisum sativum*. Phytochemistry.

[199] Kurashov E.A., Fedorova E.V., Krylova J.V., Mitrukova G.G. (2016). Assessment of the Potential Biological Activity of Low Molecular Weight Metabolites of Freshwater Macrophytes with QSAR. Scientifica.

[200] OSADHI—Online Structural and Analytics Based Database for Herbs of India. (1). https://neist.res.in/osadhi/phytodetail.php?phyto=Allopregnanolone.

[201] Dinan L. (2001). Phytoecdysteroids: Biological aspects. Phytochemistry.

[202] Dinan L., Savchenko T., Whiting P. (2001). On the distribution of phytoecdysteroids in plants. Cell. Mol. Life Sci. CMLS.

[203] Timofeev N.P. (2006). Achievements and problems in the study, use, and prediction of the biological activity of ecdysteroids. Butlerov Commun..

[204] Sugimoto N., Kuroyanagi M., Kato T., Sato K., Tada A., Yamazaki T., Tanamoto K. (2006). Identification of the main constituents in sandarac resin, a natural gum base. Shokuhin Eiseigaku Zasshi.

[205] Suzuki Y., Saijo H., Takahashi K., Kofujita H., Ashitani T. (2018). Growth-inhibitory components in Sugi (*Cryptomeria japonica*) extracts active against *Microcystis aeruginosa*. Cogent Environ. Sci..

[206] OSADHI—Online Structural and Analytics based Database for Herbs of India. (2). https://neist.res.in/osadhi/phytodetail.php?phyto=Sandaracopimarinol.

[207] NCBI PubChem Compound Summary for CID 12314286, Sandaracopimarinol. PubChem. https://pubchem.ncbi.nlm.nih.gov/compound/12314286.

[208] Gopinath K.W., Govindachari T.R., Parthasarathy P.C., Viswanathan N. (1961). Structure and Stereochemistry of Polyalthic Acid, a new Diterpene Acid. Helv. Chim. Acta.

[209] Miyazawa M., Shimamura H., Nakamura S.I., Kameoka H. (1995). Antimutagenic Activity of (+)-Polyalthic Acid from *Vitex rotundifolia*. J. Agric. Food Chem..

[210] Atolani O., Olatunji G.A. (2014). Isolation and evaluation of antiglycation potential of polyalthic acid (furano-terpene) from *Daniella oliveri*. J. Pharm. Anal..

[211] Cangiano T., DellaGreca M., Fiorentino A., Isidori M., Monaco P., Zarrelli A. (2001). Lactone diterpenes from the aquatic plant *Potamogeton natans*. Phytochemistry.

[212] Santiago M.B., Santos V.C.O., Teixeira S.C., Silva N.B.S., Oliveira P.F., Ozelin S.D., Furtado R.A., Tavares D.C., Ambrósio S.R., Veneziani R.C.S. (2023). Polyalthic Acid from *Copaifera lucens* Demonstrates Anticariogenic and Antiparasitic Properties for Safe Use. Pharmaceuticals.

[213] Mizuno C.S., Alves Dos Santos R., Nascimento Silveira N. (2025). The Biological Activities of Ent-Polyalthic Acid. Chem. Biodivers..

[214] Waridel P., Wolfender J.L., Lachavanne J.B., Hostettmann K. (2003). Ent-Labdane diterpenes from the aquatic plant *Potamogeton pectinatus*. Phytochemistry.

[215] Waridel P., Wolfender J., Lachavanne J., Hostettmann K. (2004). Ent-Labdane glycosides from the aquatic plant *Potamogeton lucens* and analytical evaluation of the lipophilic extract constituents of various *Potamogeton* species. Phytochemistry.

[216] Karagiannis S., Lanaridis P., Salaha M. (2000). Determination of benzothiazole in grapes and wines. OENO One.

[217] Rohloff J., Bones A.M. (2005). Volatile profiling of *Arabidopsis thaliana*—Putative olfactory compounds in plant communication. Phytochemistry.

[218] Hu Z., Shen Y., Shen F., Luo Y., Su X. (2009). Evidence for the signaling role of methyl jasmonate, methyl salicylate and benzothiazole between poplar (*Populus simonii* × *P. pyramidalis* ‘Opera 8277’) cuttings. Trees.

[219] Sadiq A., Zeb A., Ullah F., Ahmad S., Ayaz M., Rashid U., Muhammad N. (2018). Chemical Characterization, Analgesic, Antioxidant, and Anticholinesterase Potentials of Essential Oils from *Isodon rugosus* Wall. ex. Benth. Front. Pharmacol..

[220] Esmat A.U., Mittapally S., Begum S. (2020). GC-MS Analysis of Bioactive Compounds and Phytochemical Evaluation of the Ethanolic Extract of *Gomphrena globosa* L. Flowers. J. Drug Deliv. Ther..

[221] Babu A., Anand D., Saravanan P. (2017). Phytochemical Analysis of *Ficus arnottiana* (Miq.) Miq. Leaf Extract Using GC-MS Analysis. Int. J. Pharmacogn. Phytochem. Res..

[222] Nwankwo N.E., David J.C. (2025). A review of sulfur-containing compounds of natural origin with insights into their Pharmacological and toxicological impacts. Discov. Chem..

[223] Watson S.B. (2003). Cyanobacterial and eukaryotic algal odour compounds: Signals or by-products? A review of their biological activity. Phycologia.

[224] Cheng F., Cheng Z., Meng H., Tang X. (2016). The garlic allelochemical diallyl disulfide affects tomato root growth by influencing cell division, phytohormone balance and expansin gene expression. Front. Plant Sci..

[225] Cheng F., Cheng Z.-H., Meng H.-W. (2016). Transcriptomic insights into the allelopathic effects of the garlic allelochemical diallyl disulfide on tomato roots. Sci. Rep..

[226] Xie Y., Tian L., Han X., Yang Y. (2021). Research Advances in Allelopathy of Volatile Organic Compounds (VOCs) of Plants. Horticulturae.

[227] (2011). Algae, Sea Grasses and Products Made from Them. Methods for Determining Organoleptic and Physical Indicators.

[228] Hassanpouraghdam M.B., Hassani A., Vojodi L., Farsad-Akhtar N. (2010). Drying method affects essential oil content and composition of Basil (*Ocimum basilicum* L.). J. Essent. Oil Bear. Plants.

[229] Caputo L., Amato G., Bartolomeis P., De Martino L., Manna F., Nazzaro F., De Feo V., Barba A.A. (2022). Impact of drying methods on the yield and chemistry of *Origanum vulgare* L. essential oil. Sci. Rep..

[230] Skvortsova O.N. (1998). Regulatory documents on the storage of medicinal plant materials. New Pharm. [Novaya Apteka].

[231] Patten B.C. (1956). Notes on the biology of *Myriophyllum spicatum* L. in a New Jersey Lake. Bull. Torrey Bot. Club.

[232] Aiken S.G., Newroth P.R., Wile I. (1979). The biology of Canadian weeds. 34. *Myriophyllum spicatum* L.. Can. J. Plant Sci..

[233] Nichols S.A., Shaw B.H. (1986). Ecological life histories of the three aquatic nuisance plants, *Myriophyllum spicatum*, *Potamogeton crispus* and *Elodea canadensis*. Hydrobiologia.

[234] Gubanov I.A., Gubanov I.A., Kiseleva K.V., Novikov V.S., Tikhomirov V.N., Tikhomirov M. (2003). *Myriophyllum spicatum* L.—Spiked Water-milfoil. Illustrated Guide to Plants of Central Russia.

[235] Reed C.F. (1977). History and distribution of Eurasian watermilfoil in the United States and Canada. Phytologia.

[236] Anderson R.R., Brown R.G., Rappleye R.D. (1965). Mineral composition of Eurasian water milfoil; *Myriophyllum spicatum*, L.. Chesap. Sci..

[237] Smith C.S., Barko J.W. (1990). Ecology of Eurasian watermilfoil. J. Aquat. Plant Manag..

[238] Cook C.D.K., Nicholls M.S. (1986). A monographic study of the genus *Sparganium* (Sparganiaceae). Part 1: Subgenus. *Xanthosparganium*. Bot. Helv..

[239] Cook C.D.K., Nicholls M.S. (1987). A monographic study of the genus *Sparganium* (Sparganiaceae). Part 2: Subgenus. *Sparganium*. Bot. Helv..

[240] Sulman J.D., Drew B.T., Drummond C., Hayasaka E., Sytsma K.J. (2013). Systematics, biogeography, and character evolution of Sparganium (Typhaceae): Diversification of a widespread, aquatic lineage. Am. J. Bot..

[241] Mochalova O.A., Efimov D.Y. (2022). Environmental Patterns of Distribution of *Sparganium emersum* and *S. hyperboreum* (Typhaceae) in Northeast Asia. Inland Water Biol..

[242] Belyakov E.A., Lapirov A.G. (2018). Morphological and Ecological Cenotic Features of the Relict Species *Sparganium gramineum* Georgi (Typhaceae) in Waterbodies of European Russia. Inland Water Biol..

[243] Murphy K.J. (2002). Plant communities and plant diversity in softwater lakes of northern Europe. Aquat. Bot..

[244] Wiegleb G., Kaplan Z. (1998). An account of the species of *Potamogeton* L. (Potamogetonaceae). Folia Geobot..

[245] Choi K., Hwang Y., Hong J.K., Kang J.S. (2023). Comparative Plastid Genome and Phylogenomic Analyses of *Potamogeton* Species. Genes.

[246] Plants of the World Online (Kew Science) *Potamogeton* L.. https://powo.science.kew.org/taxon/urn%3Alsid%3Aipni.org%3Anames%3A30005042-2?utm_source=chatgpt.com.

[247] Sleptsov I.V., Mikhailov V.V., Filippova V.A., Barinova S., Gabysheva O.I., Gabyshev V.A. (2025). Microalgae Indicators of Metabolic. Changes in *Potamogeton perfoliatus* L. Under Different Growing Conditions of Urban Territory Lakes in a Permafrost Area. Sustainability.

[248] Malea L., Nakou K., Papadimitriou A., Exadactylos A., Orfanidis S. (2021). Physiological Responses of the Submerged Macrophyte *Stuckenia pectinata* to High Salinity and Irradiance Stress to Assess Eutrophication Management and Climatic Effects: An Integrative Approach. Water.

[249] Hellquist C.B., Thorne R.F., Haynes R.R. (2012). *Potamogeton natans*, in Jepson Flora Project (eds.) Jepson eFlora. https://ucjeps.berkeley.edu/eflora/eflora_display.php?tid=39591.

[250] Chopik V.I., Dudchenko L.G., Krasnova A.N. (1983). Wild Useful Plants of Ukraine. Handbook.

[251] Plants of the World Online *Persicaria amphibia* (L.) Delarbre. Royal Botanic Gardens, Kew. https://powo.science.kew.org/taxon/urn%3Alsid%3Aipni.org%3Anames%3A30193627-2?utm_source=chatgpt.com.

[252] Mitchell R.S. (1968). Variation in the Polygonum amphibium Complex and Its Taxonomic Significance.

[253] Hinds H.R., Freeman C.C. (2005). Persicaria. Flora of North America Editorial Committee, Flora of North America North of Mexico.

[254] Gubanov I.A., Kiseleva K.V., Novikov V.S., Tikhomirov V.N. (2003). Illustrated Guide to Plants of Central Russia.

[255] Plants of the World Online *Nuphar lutea* (L.) Sm.: Taxon information (Native Range; Life Form; First Publication in Fl. Graec. Prodr. 1:361, 1809). https://powo.science.kew.org/taxon/urn%3Alsid%3Aipni.org%3Anames%3A30385379-2?utm_source=chatgpt.com.

[256] Padgett D.J. (2007). A monograph of Nuphar (Nymphaeaceae). Rhodora.

[257] Kok C.J., Van der Velde G., Landsbergen K.M. (1990). Production, nutrient dynamics and initial decomposition of floating leaves of *Nymphaea alba* L. and *Nuphar lutea* (L.) Sm. (Nymphaeaceae) in alkaline and acid waters. Biogeochemistry.

[258] Henriot C.P., Cuenot Q., Levrey L.H., Loup C., Chiarello L., Masclaux H., Bornette G. (2019). Relationships between key functional traits of the waterlily *Nuphar lutea* and wetland nutrient content. PeerJ.

[259] Raspopov I.M. (1985). Higher Aquatic Vegetation of Large Lakes of the North-West USSR.

[260] Gubanov I.A., Kiseleva K.V., Novikov V.S., Tikhomirov V.N. (2004). Illustrated Guide to Plants of Central, R. Volume 3. Angiosperms (Flowering) Plants.

[261] Møller C.L., Sand-Jensen K. (2011). High sensitivity of *Lobelia dortmanna* to sediment oxygen depletion following organic enrichment. New Phytol..

[262] Nielsen S.R., Martinsen K.T., Pedersen O., Baastrup-Spohr L. (2023). Reasons for the dramatic loss of *Lobelia dortmanna*, a keystone plant species of softwater lakes in the. Northern Hemisphere. Freshw. Biol..

[263] Winkel A., Borum J. (2009). Use of sediment CO_2_ by submersed rooted plants. Ann. Bot..

[264] Plants of the World Online (POWO) *Ceratophyllum demersum* L. Kew Science. https://powo.science.kew.org/taxon/urn:lsid:ipni.org:names:30006047-2.

[265] Jones E.N. (1931). The morphology and biology of *Ceratophyllum demersum*. Univ. Iowa Stud. Nat. Hist..

[266] Cook C.D.K. (1996). Aquatic Plant Book.

[267] Szabó S., Fedor N., Koleszár G., Braun M., Korponai J., Kočić A., Hilt S., Oláh V. (2025). Submerged macrophytes can maintain stable dominance over free-floating competitors through high pH. Freshw. Biol..

[268] Clevenger J.F. (1928). Apparatus for the determination of volatile oil. J. Am. Pharm. Assoc..

[269] (1980). Medicinal Plant Materials. Methods for Determining Moisture, Ash Content, Extractive and Tannin Content of Essential Oil.

[270] (1989). Essential Oil Crop Fruits. Industrial Raw Materials. Methods for Determining the Mass Fraction of Essential Oil.

[271] Anderson T.A., Guthrie E.A., Walton B.T. (1993). Bioremediation in the rhizosphere. Environ. Sci. Technol..

[272] (1989). State Pharmacopoeia of the USSR (Gosudarstvennaya Farmakopeya SSSR).

[273] Tkachev A.V. (2008). Study of Plant Volatiles (Issledovanie Letuchikh Veshchestv Rastenii).

[274] Maurer H.H., Pfleger K., Weber A.A. (2011). Mass Spectral and GC Data of Drugs, Poisons, Pesticides, Pollutants and Their Metabolites.

[275] NIST/EPA/NIH MS/MS Mass Spectral Library, 2014. https://www.sisweb.com/software/nist-msms-2014.htm.

[276] Zellner B.A., Bicchi C., Dugo P., Rubiolo P., Dugo G., Mondello L. (2008). Linear retention indices in gas chromatographic analysis: A review. Flavour Fragr. J..

[277] Kucharski Ł., Cybulska K., Kucharska E., Nowak A., Pełech R., Klimowicz A. (2022). Biologically Active Preparations from the Leaves of Wild Plant Species of the Genus *Rubus*. Molecules.

[278] Vickackaite V., Pilaityte K., Poskus V. (2025). Extraction, Isolation, and Purification of Furanocoumarins from Invasive *Heracleum sosnowskyi*. Separations.

[279] Jaccard P. (1901). Distribution de la flore alpine dans le basin des regions voisines. Bull. De La Société Vaudoise Des Sci. Nat..

[280] Czekanowski J. (1922). Coefficient of ratial likeness and durchschnittliche differenz. Anthropol. Anz..

[281] Sorensen T.A. (1948). A method of establishing groups of equal amplitude in plant sociology based on similarity of species content, and its application to analyses of the vegetation on Danish commons. K. Dan. Vidensk. Selsk. Biol. Skr..

[282] Morisita M. (1959). Measuring of interspecific association and similarity between communities. Mem. Fac. Sci. Kyushu Univ. Ser. E (Biol.).

[283] Pimentel-Gomes F. (2009). Curso de Estatística Experimental.

[284] Sallin V.P., Lima D.V.C., Rodrigues M.J.L., Rossi M.T., Oliveira V.S., Schmildt E.R. (2022). Classification to coefficient of variation in physical and chemical attributes of oranges. Braz. J. Exp. Des. Data Anal. Inferent. Stat. (BJEDIS).

[285] Kumar A., Sankalp S., Remesan R., Kasiviswanathan K.S., Soundharajan D., Patidar S., He J., Ojha C.S.P. (2023). Chapter 1—Spatiotemporal rainfall variability and trend analysis over all the districts of West Bengal during 1980–2021. Modeling and. Mitigation Measures for Managing Extreme Hydrometeorological Events Under a Warming Climate (Developments in Environmental Science).

[286] StatSoft, Inc (2011). STATISTICA (Data Analysis Software System), Version 10.

[287] Hu H., Hong Y. (2008). Algal-bloom control by allelopathy of aquatic macrophytes—A review. Front. Environ. Sci. Eng. China.

[288] Zhu X., Dao G., Tao Y., Zhan X., Hu H. (2021). A review on control of harmful algal blooms by plant-derived allelochemicals. J. Hazard. Mater..

