# Supplementary material for: Major Low-Molecular-Weight Metabolites from Freshwater Aquatic Macrophytes: Ecological Aspects
Source: Molecules. 2026 Mar 8;31(5):895. doi: 10.3390/molecules31050895 (PMC12985617; doi:10.3390/molecules31050895)
Supplement: Supplementary file 1 [file molecules-31-00895-s001.zip › molecules-4150810-supplementary.pdf]

Table S1. Component composition of major LMWOCs of investigated species (RI – retention index; % – maximum percentage content of a substance from the sum of all substances; C – maximum absolute content of substance, μg/g dry plant weight).

№	Substances / Chemical group	Formula	RI	*M. spicatum*		*S. × foliosum*		*P. amphibia*		*P. perfoliatus*		*N. lutea*		*S. gramineum*		*S. emersum*		*P. pectinatus*		*P. natans*		*L. dortmanna*		*C. demersum*	
				%	C	%	C	%	C	%	C	%	C	%	C	%	C	%	C	%	C	%	C	%	C
1	3,5-dimethylheptane / h	C_9H_{20}	787	–	–	–	–	–	–	–	–	–	–	–	–	–	–	–	–	–	–	3.06	6.08	–	–
2	hexan-3-one / k	$C_6H_{12}O$	791	–	–	–	–	–	–	3.04	0.98	–	–	–	–	–	–	–	–	–	–	–	–	–	–
3	2,3-dimethylheptane / h	C_9H_{20}	792	–	–	–	–	–	–	–	–	–	–	–	–	–	–	–	–	–	–	3.08	6.12	–	–
4	hexan-2-one / k	$C_6H_{12}O$	794	–	–	–	–	1.62	2.38	4.42	1.42	–	–	–	–	–	–	2.14	1.88	–	–	–	–	–	–
5	Hexanal / ald	$C_6H_{12}O$	799	–	–	–	–	4.28	5.74	3.35	1.08	–	–	–	–	–	–	4.29	5.22	–	–	–	–	5.08	2.84
6	hexan-2-ol / a	$C_6H_{14}O$	802	–	–	–	–	–	–	1.04	0.33	–	–	–	–	–	–	–	–	–	–	–	–	–	–
7	(*E*)-hex-2-en-1-ol / a	$C_6H_{12}O$	838	–	–	–	–	–	–	1.93	0.77	–	–	–	–	–	–	7.71	6.86	–	–	–	–	–	–
8	furan-2-carbaldehyde; [furfural] / ald	$C_5H_4O_2$	841	–	–	–	–	–	–	–	–	–	–	–	–	–	–	–	–	–	–	1.12	2.23	–	–
9	(*E*)-hex-2-enal / ald	$C_6H_{10}O$	843	–	–	–	–	3.02	2.30	10.86	5.64	–	–	–	–	–	–	–	–	–	–	–	–	–	–
10	heptan-2-one / k	$C_7H_{14}O$	894	–	–	–	–	–	–	–	–	–	–	–	–	–	–	–	–	–	–	–	–	1.68	0.94
11	1,2-xylene / ah	C_8H_{10}	897	–	–	–	–	–	–	1.12	1.33	–	–	–	–	–	–	–	–	–	–	–	–	–	–
12	heptanal / ald	$C_7H_{14}O$	902	–	–	1.46	2.33	–	–	–	–	–	–	–	–	–	–	3.10	2.72	–	–	–	–	2.26	1.26
13	hept-4-enal / ald	$C_7H_{12}O$	903	–	–	–	–	–	–	–	–	–	–	–	–	–	–	–	–	–	–	–	–	2.81	1.57
14	Benzaldehyde / ald	C_7H_6O	949	–	–	–	–	–	–	1.69	0.54	–	–	–	–	–	–	2.27	2.76	–	–	–	–	1.35	0.75
15	2,4-dimethylocta-2,6-diene / h	$C_{10}H_{18}$	967	–	–	–	–	–	–	1.15	0.37	–	–	–	–	–	–	–	–	–	–	–	–	–	–
16	octa-1,7-dien-3-ol / a	$C_8H_{14}O$	981	–	–	1.98	2.88	–	–	–	–	–	–	–	–	–	–	–	–	–	–	–	–	–	–
17	oct-7-en-2-one / k	$C_8H_{14}O$	984	–	–	–	–	–	–	2.92	0.63	–	–	–	–	–	–	–	–	–	–	–	–	–	–
18	2-pentylfuran / e	$C_9H_{14}O$	988	2.63	4.89	–	–	1.03	0.42	1.29	0.42	1.29	95.85	–	–	–	–	1.32	1.16	–	–	–	–	1.29	0.72
19	octane-2,3-dione / k	$C_8H_{14}O_2$	989	–	–	–	–	–	–	–	–	–	–	–	–	–	–	1.30	1.14	–	–	–	–	–	–
20	(*Z*)-oct-6-en-2-one / k	$C_8H_{14}O$	994	–	–	–	–	–	–	–	–	–	–	–	–	–	–	1.28	1.12	–	–	–	–	–	–
21	2-[(*Z*)-pent-2-enyl]furan / e	$C_9H_{12}O$	999	1.60	2.97	–	–	–	–	1.51	0.49	1.04	16.94	–	–	–	–	–	–	–	–	–	–	–	–
22	(2*E*,4*E*)-hepta-2,4-dienal / ald	$C_7H_{10}O$	1009	–	–	–	–	–	–	3.09	0.99	–	–	–	–	–	–	1.07	2.17	–	–	–	–	–	–
23	(4*R*)-1-methyl-4-prop-1-en-2-ylcyclohexene; [*D*-limonene] / h	$C_{10}H_{16}$	1019	–	–	–	–	–	–	1.13	0.36	–	–	–	–	–	–	–	–	–	–	–	–	–	–
24	2,2,4,4-tetramethyloctane / h	$C_{12}H_{26}$	1025	–	–	1.19	1.14	–	–	–	–	–	–	–	–	–	–	–	–	–	–	–	–	–	–
25	2-phenylacetaldehyde / ald	C_8H_8O	1036	–	–	–	–	–	–	1.23	0.40	–	–	–	–	–	–	1.68	1.48	–	–	–	–	–	–
26	5-ethyl-2,2,3-trimethylheptane / h	$C_{12}H_{26}$	1056	–	–	1.01	0.97	–	–	–	–	–	–	–	–	–	–	–	–	–	–	–	–	–	–
27	2,6,7-trimethyldecane / h	$C_{13}H_{28}$	1063	–	–	1.90	2.37	–	–	–	–	–	–	1.65	1.82	1.20	1.40	–	–	–	–	–	–	–	–
28	(*E*)-2-octen-1-ol / a	$C_8H_{16}O$	1067	–	–	2.98	4.33	–	–	–	–	–	–	–	–	–	–	–	–	–	–	–	–	–	–
29	(3*E*,5*E*)-octa-3,5-dien-2-one / k	$C_8H_{12}O$	1072	–	–	–	–	1.01	1.49	3.64	0.98	–	–	–	–	–	–	1.19	2.42	–	–	–	–	1.20	0.67
30	3,7-dimethyldecane / h	$C_{12}H_{26}$	1077	–	–	1.42	1.63	–	–	–	–	–	–	1.13	1.25	–	–	–	–	–	–	–	–	–	–
31	(3*E*,5*E*)-nona-3,5-dien-2-one / k	$C_9H_{14}O$	1092	–	–	–	–	–	–	–	–	–	–	–	–	–	–	1.23	2.50	–	–	–	–	–	–
32	2,6-dimethylcyclohexan-1-ol / a	$C_8H_{16}O$	1099	–	–	–	–	–	–	1.76	0.57	–	–	–	–	–	–	–	–	–	–	–	–	1.28	0.72
33	2-propan-2-ylcyclohexan-1-ol /a	$C_9H_{18}O$	1104	–	–	–	–	–	–	–	–	–	–	–	–	–	–	1.85	1.62	–	–	–	–	–	–
34	nonanal / ald	$C_9H_{18}O$	1105	–	–	4.32	4.14	–	–	–	–	–	–	7.58	12.68	1.36	1.38	–	–	–	–	–	–	–	–
35	2,6,6-trimethylcyclohex-2-ene-1,4-dione; [ketoisophorone] / k	$C_9H_{12}O_2$	1135	–	–	–	–	–	–	1.90	0.61	–	–	–	–	–	–	1.87	1.65	–	–	–	–	–	–
36	(2*E*,6*E*)-nona-2,6-dienal / ald	$C_9H_{14}O$	1160	–	–	–	–	–	–	–	–	–	–	1.62	2.71	–	–	–	–	–	–	–	–	–	–
37	dodecane / h	$C_{12}H_{26}$	1200	–	–	–	–	–	–	1.88	0.60	–	–	–	–	–	–	–	–	–	–	–	–	–	–
38	unidentified compound m/z 194 [M+], 112 (100) / un	–	1202	–	–	–	–	1.16	1.48	–	–	–	–	–	–	–	–	–	–	–	–	–	–	–	–

№	Substances / Chemical group	Formula	RI	*M. spicatum*		*S. × foliosum*		*P. amphibia*		*P. perfoliatus*		*N. lutea*		*S. gramineum*		*S. emersum*		*P. pectinatus*		*P. natans*		*L. dortmanna*		*C. demersum*	
				%	C	%	C	%	C	%	C	%	C	%	C	%	C	%	C	%	C	%	C	%	C
39	1,3-benzothiazole / s	C_7H_5NS	1209	–	–	–	–	2.09	3.08	1.05	0.23	–	–	–	–	–	–	–	–	–	–	–	–	–	–
40	5-methyl-2-propan-2-ylphenol; [thymol] / p	$C_{10}H_{14}O$	1300	–	–	–	–	–	–	1.95	0.42	–	–	–	–	–	–	–	–	–	–	–	–	–	–
41	tetradecane / h	$C_{14}H_{30}$	1400	–	–	–	–	–	–	2.05	0.66	–	–	–	–	–	–	–	–	–	–	–	–	–	–
42	6,10-dimethylundecan-2-one / k	$C_{13}H_{26}O$	1405	–	–	–	–	–	–	–	–	–	–	–	–	–	–	1.86	1.64	–	–	–	–	–	–
43	(5*E*)-6,10-dimethylundeca-5,9-dien-2-one; [geranylacetone] / k	$C_{13}H_{22}O$	1454	–	–	–	–	–	–	1.46	0.47	–	–	–	–	–	–	–	–	–	–	–	–	–	–
44	(*E*)-4-(2,6,6-trimethylcyclohexen-1-yl)but-3-en-2-one; [β-Ionone] / k	$C_{13}H_{20}O$	1478	–	–	–	–	1.06	1.55	5.28	1.75	–	–	–	–	–	–	3.11	6.31	–	–	–	–	6.74	3.77
45	(*E*)-4-(2,6,6-trimethylcyclohexen-1-yl)but-3-en-2-ol; [β-ionol] / a	$C_{13}H_{22}O$	1479	–	–	–	–	–	–	–	–	–	–	–	–	–	–	4.68	4.11	–	–	–	–	–	–
46	4-(2,6,6-trimethylcyclohexen-1-yl)but-3-en-2-one / k	$C_{13}H_{20}O$	1480	1.98	5.04	–	–	–	–	3.45	0.75	–	–	–	–	–	–	–	–	–	–	–	–	–	–
47	5,5-dimethyl-2-propylcyclohexane-1,3-dione / k	$C_{11}H_{18}O_2$	1509	–	–	–	–	–	–	–	–	–	–	–	–	–	–	–	–	–	–	–	–	2.23	1.25
48	4-[(1*R*,2*R*)-2-methyl-3-oxocyclohexyl]butanal / df	$C_{11}H_{18}O_2$	1511	–	–	–	–	–	–	1.51	0.33	–	–	–	–	–	–	–	–	–	–	–	–	–	–
49	8*a*-methyl-3,4,4*a*,5,6,7-hexahydro-2*H*-naphthalene-1,8-dione / k	$C_{11}H_{16}O_2$	1514	–	–	–	–	–	–	–	–	–	–	–	–	–	–	–	–	–	–	–	–	1.56	0.87
50	dodecanoic acid; [lauric acid] / fa	$C_{12}H_{24}O_2$	1584	1.00	2.86	–	–	1.78	2.62	–	–	–	–	–	–	–	–	–	–	–	–	–	–	–	–
51	hexadecane / h	$C_{16}H_{34}$	1600	–	–	–	–	1.12	1.44	–	–	–	–	–	–	–	–	–	–	–	–	–	–	–	–
52	tetradecanal / ald	$C_{14}H_{28}O$	1612	1.10	2.81	–	–	–	–	–	–	–	–	–	–	–	–	–	–	–	–	–	–	–	–
53	(*E*)-heptadec-8-ene / h	$C_{17}H_{34}$	1677	–	–	–	–	–	–	1.18	1.02	–	–	–	–	–	–	–	–	–	–	–	–	–	–
54	tetradecan-1-ol / a	$C_{14}H_{30}O$	1682	3.70	6.89	–	–	–	–	–	–	–	–	–	–	–	–	–	–	–	–	–	–	–	–
55	heptadecane /h	$C_{17}H_{36}$	1700	4.25	7.92	–	–	–	–	1.74	1.50	–	–	–	–	–	–	2.82	5.72	–	–	–	–	2.97	1.66
56	pentadecanal / ald	$C_{15}H_{30}O$	1711	5.82	10.84	5.64	5.41	–	–	2.12	1.55	1.00	74.28	1.56	2.45	1.67	1.95	–	–	2.28	10.95	–	–	3.37	1.88
57	(*Z*)-tetradec-11-enoic acid / fa	$C_{14}H_{26}O_2$	1745	–	–	1.21	1.43	–	–	–	–	–	–	–	–	–	–	–	–	–	–	–	–	–	–
58	tetradecanoic acid; [myristic acid] / fa	$C_{14}H_{28}O_2$	1763	6.63	32.08	4.74	5.58	8.33	12.25	4.11	11.05	5.01	229.91	1.27	1.15	1.81	1.84	14.55	22.37	1.76	18.74	3.46	6.88	1.12	0.63
59	6,10,14-trimethylpentadecan-2-one [phytone] / k	$C_{18}H_{36}O$	1845	4.57	8.52	1.91	2.04	5.23	7.69	4.25	3.77	–	–	–	–	2.62	2.66	9.09	8.55	1.48	15.79	–	–	4.71	2.63
60	pentadecanoic acid; [pentadecylic acid] / fa	$C_{15}H_{30}O_2$	1847	1.26	2.34	–	–	1.74	2.56	6.48	5.59	–	–	–	–	–	–	–	–	–	–	–	–	–	–
61	hexadecan-1-ol / a	$C_{16}H_{34}O$	1888	–	–	–	–	–	–	4.47	1.78	–	–	–	–	–	–	1.61	1.42	2.79	29.77	–	–	–	–
62	heptadeca-5,8,11-trien-1-ol / a	$C_{17}H_{30}O$	1889	1.66	4.23	–	–	–	–	–	–	–	–	–	–	–	–	–	–	–	–	–	–	–	–
63	nonadec-1-ene / h	$C_{19}H_{38}$	1890	–	–	–	–	–	–	–	–	–	–	–	–	–	–	–	–	1.56	5.93	–	–	–	–
64	heptadecan-1-ol / a	$C_{17}H_{36}O$	1890	–	–	–	–	–	–	4.37	5.17	–	–	–	–	–	–	–	–	–	–	–	–	–	–
65	(7*Z*,10*Z*,13*Z*)-hexadeca-7,10,13-trienal / ald	$C_{16}H_{26}O$	1891	2.11	5.36	–	–	–	–	1.41	0.56	–	–	–	–	–	–	–	–	–	–	–	–	1.23	0.69
66	2-methylhexadecan-1-ol /a	$C_{16}H_{34}O$	1893	–	–	–	–	–	–	1.29	0.28	–	–	–	–	–	–	–	–	–	–	–	–	–	–
67	5-(4,8-dimethylnonyl)-5-methyloxolan-2-one / e	$C_{16}H_{30}O_2$	1897	–	–	–	–	–	–	–	–	–	–	–	–	–	–	1.28	1.13	–	–	–	–	–	–
68	nonadecane / h	$C_{19}H_{40}$	1900	–	–	–	–	–	–	–	–	–	–	–	–	–	–	–	–	–	–	–	–	1.13	0.63
69	(5*E*,9*E*)-6,10,14-trimethylpentadeca-5,9,13-trien-2-one / k	$C_{18}H_{30}O$	1916	–	–	–	–	–	–	1.82	0.59	–	–	–	–	–	–	1.30	2.20	–	–	–	–	–	–
70	(4a*S*,4b*S*,7*R*,10a*S*)-7-ethenyl-1,1,4a,7-tetramethyl-3,4,4b,5,6,9,10,10a-octahydro-2H-phenanthrene; [Sandaracopimaradiene] / h	$C_{20}H_{32}$	1917	–	–	–	–	–	–	2.40	6.44	–	–	–	–	–	–	–	–	–	–	–	–	–	–
71	(4a*S*,8*S*,8a*S*)-4,4,8a-trimethyl-7-methylidene-8-(3-methylpenta-2,4-dienyl)-2,3,4a,5,6,8-hexahydro-1H-naphthalene; [biformene] / h	$C_{20}H_{32}$	1918	–	–	–	–	–	–	2.44	0.97	–	–	–	–	–	–	–	–	–	–	–	–	1.84	1.03
72	cyclohexadec-8-en-1-one / k	$C_{16}H_{28}O$	1951	–	–	–	–	–	–	–	–	–	–	–	–	–	–	–	–	–	–	4.75	9.45	–	–
73	3,7,11,15-tetramethylhexadec-1-en-3-ol; [isophytol] / a	$C_{20}H_{40}O$	1954	–	–	–	–	–	–	–	–	–	–	–	–	–	–	–	–	2.56	27.31	–	–	–	–
74	hexadecanoic acid; [palmitic acid] / fa	$C_{16}H_{32}O_2$	1960	10.56	56.84	11.03	16.82	42.53	54.45	13.55	32.14	29.06	2156.62	17.17	17.60	15.05	15.27	23.92	30.88	16.57	176.50	34.40	68.38	7.86	4.39

№	Substances / Chemical group	Formula	RI	*M. spicatum*		*S. × foliosum*		*P. amphibia*		*P. perfoliatus*		*N. lutea*		*S. gramineum*		*S. emersum*		*P. pectinatus*		*P. natans*		*L. dortmanna*		*C. demersum*	
				%	C	%	C	%	C	%	C	%	C	%	C	%	C	%	C	%	C	%	C	%	C
75	(2*S*,4a*S*,4b*S*,8a*S*)-2-ethenyl-2,4b,8,8-tetramethyl-3,4,4a,5,6,7,8a,9-octahydro-1H-phenanthrene; [Isopimaradiene] / h	$C_{20}H_{32}$	1970	–	–	–	–	–	–	2.39	1.17	–	–	–	–	–	–	–	–	–	–	–	–	–	–
76	cis-9-Hexadecenoic acid; [Palmitoleic acid] / fa	$C_{16}H_{30}O_2$	1971	11.38	68.61	1.19	1.41	5.43	7.99	–	–	4.75	352.35	–	–	–	–	–	–	–	–	2.10	4.17	–	–
77	(*Z*)-hexadec-11-enoic acid / fa	$C_{16}H_{30}O_2$	1977	–	–	–	–	–	–	4.90	5.80	–	–	–	–	–	–	2.51	5.10	–	–	–	–	–	–
78	unidentified compound m/z 244 [M+], 81 (100) / un	–	1995	1.31	3.33	–	–	–	–	–	–	–	–	–	–	–	–	–	–	–	–	–	–	–	–
79	(3*S*,5*S*,8*S*,9*S*,10*S*,13*S*,14*S*)-9,10,13-trimethyl-1,2,3,4,5,6,7,8,11,12,14,15,16,17-tetradecahydrocyclopenta[a]phenanthren-3-ol; [9-methylandrostan-3-ol] / df	$C_{20}H_{34}O$	1996	–	–	–	–	–	–	–	–	–	–	–	–	–	–	–	–	1.35	5.89	–	–	–	–
80	octadecanal / ald	$C_{18}H_{36}O$	2010	–	–	–	–	–	–	–	–	–	–	3.40	5.69	–	–	–	–	–	–	–	–	–	–
81	10-methylicosane / h	$C_{21}H_{44}$	2035	–	–	1.63	1.92	–	–	–	–	–	–	3.03	2.27	1.43	1.49	–	–	–	–	–	–	–	–
82	5-(5,5,8a-trimethyl-2-methylidene-3,4,4a,6,7,8-hexahydro-1H-naphthalen-1-yl)-3-methylpent-1-en-3-ol (isomer) / a	$C_{20}H_{34}O$	2042	–	–	–	–	–	–	9.35	2.02	–	–	–	–	–	–	–	–	–	–	–	–	–	–
83	(6*E*,10*E*)-3,7,11,15-tetramethylhexadeca-1,6,10,14-tetraen-3-ol; [geranyllinalool] / a	$C_{20}H_{34}O$	2044	–	–	–	–	–	–	–	–	–	–	–	–	–	–	7.14	13.48	1.19	5.18	–	–	–	–
84	(3*R*)-5-[(1*S*,4a*S*,8a*S*)-5,5,8a-trimethyl-2-methylidene-3,4,4a,6,7,8-hexahydro-1H-naphthalen-1-yl]-3-methylpent-1-en-3-ol; [manool] / a	$C_{20}H_{34}O$	2045	–	–	–	–	–	–	16.44	44.18	–	–	–	–	–	–	4.13	8.40	19.61	74.72	–	–	3.36	1.88
85	(5*S*,8*S*,9*S*,10*R*,13*S*,14*S*)-10,13-dimethyl-5,6,7,8,9,12,14,15,16,17-decahydro-4H-cyclopenta[a]phenanthrene-3,11-dione; [androst-1-ene-3,11-dione] / df	$C_{19}H_{26}O_2$	2065	–	–	–	–	–	–	2.54	6.84	–	–	–	–	–	–	–	–	–	–	–	–	–	–
86	(3*S*,5*R*,8*S*,9*S*,10*R*,13*S*,14*S*)-4,4,10,13-tetramethyl-1,2,3,5,6,7,8,9,11,12,14,15,16,17-tetradecahydrocyclopenta[a]phenanthren-3-ol; [4,4-dimethylandrostan-3-ol] / a	$C_{21}H_{36}O$	2068	–	–	–	–	–	–	2.99	2.02	–	–	–	–	–	–	–	–	–	–	–	–	–	–
87	octadecan-1-ol / a	$C_{18}H_{38}O$	2090	–	–	–	–	–	–	1.33	1.58	–	–	–	–	–	–	–	–	–	–	–	–	–	–
88	heneicosane / h	$C_{21}H_{44}$	2100	1.54	6.29	–	–	2.01	2.23	3.13	3.85	2.33	172.89	–	–	–	–	3.54	3.11	–	–	4.52	8.99	–	–
89	(*E*,7*R*,11*R*)-3,7,11,15-tetramethylhexadec-2-en-1-ol; [phytol] / a	$C_{20}H_{40}O$	2118	16.39	41.75	9.58	15.21	4.14	3.31	10.08	17.20	6.04	448.31	6.30	6.20	13.05	15.16	3.66	4.26	6.72	36.63	–	–	1.28	0.72
90	methyl octadecanoate / e	$C_{19}H_{38}O_2$	2126	–	–	–	–	–	–	2.69	2.92	–	–	–	–	–	–	4.54	3.98	–	–	–	–	6.28	3.51
91	(9*Z*,12*Z*)-octadeca-9,12-dienoic acid; [linoleic acid] / fa	$C_{18}H_{32}O_2$	2140	17.00	102.45	1.41	2.35	5.48	6.37	3.14	8.44	25.03	1857.81	1.86	1.39	1.06	1.07	–	–	5.19	22.69	4.07	8.08	3.03	1.70
92	(9*Z*,12*Z*,15*Z*)-octadeca-9,12,15-trienoic acid; [α-linolenic acid] / fa	$C_{18}H_{30}O_2$	2141	7.46	44.97	–	–	10.43	8.09	18.41	21.79	31.31	807.98	5.35	4.00	–	–	–	–	2.06	21.93	3.36	6.68	2.60	1.45
93	1,1-bis(prop-2-en-1-yloxy)tetradecane / ald	$C_{20}H_{38}O_2$	2143	–	–	–	–	–	–	1.02	0.88	–	–	–	–	–	–	–	–	–	–	–	–	–	–
94	(3*S*,4a*R*,6a*S*,10a*S*,10b*R*)-3-ethenyl-3,4a,7,7,10a-pentamethyl-2,5,6,6a,8,9,10,10b-octahydro-1H-benzo[f]chromene; [epimanoyl oxide] / e	$C_{20}H_{34}O$	2146	–	–	–	–	–	–	–	–	–	–	–	–	–	–	–	–	2.02	21.47	–	–	–	–
95	ethyl octadeca-9,12-dienoate / e	$C_{20}H_{36}O_2$	2168	–	–	–	–	–	–	–	–	–	–	–	–	–	–	–	–	2.50	10.91	–	–	–	–
96	nonadeca-1,18-diene-7,10-dione / k	$C_{19}H_{32}O_2$	2175	–	–	–	–	–	–	1.40	3.75	–	–	–	–	–	–	1.81	1.59	–	–	–	–	–	–
97	1-methyl-7-propan-2-ylphenanthrene; [retene] / ah	$C_{18}H_{18}$	2199	–	–	–	–	1.00	1.28	–	–	–	–	–	–	–	–	–	–	–	–	–	–	–	–
98	docosane / h	$C_{22}H_{46}$	2200	–	–	–	–	–	–	–	–	–	–	1.34	1.00	–	–	–	–	–	–	–	–	–	–
99	[(3*S*,8*R*,9*S*,10*R*,13*S*,14*S*)-10,13-dimethyl-17-oxo-1,2,3,4,7,8,9,11,12,14,15,16-dodecahydrocyclopenta[a]phenanthren-3-yl] acetate; [dehydroepiandrosterone acetate] / df	$C_{19}H_{28}O_2$	2204	–	–	–	–	–	–	–	–	–	–	–	–	–	–	–	–	2.67	10.17	–	–	–	–

№	Substances / Chemical group	Formula	RI	*M. spicatum*		*S. × foliosum*		*P. amphibia*		*P. perfoliatus*		*N. lutea*		*S. gramineum*		*S. emersum*		*P. pectinatus*		*P. natans*		*L. dortmanna*		*C. demersum*	
				%	C	%	C	%	C	%	C	%	C	%	C	%	C	%	C	%	C	%	C	%	C
100	methyl (1*R*,4a*R*,4b*S*,7*S*,10a*R*)-7-ethyl-1,4a,7-trimethyl-3,4,4b,5,6,9,10,10a-octahydro-2H-phenanthrene-1-carboxylate / e	$C_{21}H_{34}O_2$	2205	–	–	–	–	–	–	–	–	–	–	–	–	–	–	–	–	2.41	25.65	–	–	–	–
101	3,7,11,15-tetramethylhexadec-2-enyl acetate / e	$C_{22}H_{42}O_2$	2224	4.11	21.26	–	–	–	–	–	–	–	–	–	–	–	–	–	–	–	–	–	–	–	–
102	(Z)-icos-9-en-1-ol / a	$C_{20}H_{40}O$	2235	–	–	–	–	–	–	–	–	–	–	2.23	1.67	–	–	–	–	–	–	–	–	–	–
103	1-[(3*R*,5*R*,8*R*,9*S*,10*S*,13*S*,14*S*,17*S*)-3-hydroxy-10,13-dimethyl-2,3,4,5,6,7,8,9,11,12,14,15,16,17-tetradecahydro-1H-cyclopenta[a]phenanthren-17-yl]ethanone; [pregnanolone-like steroid; tentative library ID] / df	$C_{21}H_{34}O_2$	2244	–	–	–	–	–	–	7.59	1.64	–	–	–	–	–	–	–	–	–	–	–	–	1.67	0.93
104	(8*R*,9*S*,10*R*,13*S*,14*S*)-10,13-dimethyl-1,2,7,8,9,11,12,14,15,16-decahydrocyclopenta[a]phenanthrene-3,6,17-trione; [Androst-4-ene-3,6,17-trione] / k	$C_{19}H_{24}O_3$	2245	–	–	–	–	–	–	13.13	35.29	–	–	–	–	–	–	–	–	–	–	–	–	–	–
105	(2*E*)-5-[5-(Hydroxymethyl)-5,8a-dimethyl-2-methylenedecahydro-1-naphthalenyl]-3-methyl-2-penten-1-ol; [agathadiol] / a	$C_{20}H_{34}O_2$	2246	–	–	–	–	–	–	–	–	–	–	–	–	–	–	–	–	1.39	14.76	–	–	–	–
106	(1*R*,2*R*,4a*S*,8a*S*)-1-[(3*R*)-3-hydroxy-3-methylpent-4-enyl]-2,5,5,8a-tetramethyl-3,4,4a,6,7,8-hexahydro-1H-naphthalen-2-ol; [sclareol] / a	$C_{20}H_{36}O_2$	2249	–	–	–	–	–	–	–	–	–	–	–	–	–	–	–	–	3.77	16.49	–	–	–	–
107	[[(1*R*,4a*R*,4b*S*,7*R*,10a*R*)-7-ethenyl-1,4a,7-trimethyl-3,4,4b,5,6,9,10,10a-octahydro-2H-phenanthren-1-yl]methanol] / a	$C_{20}H_{32}O$	2280	–	–	–	–	–	–	1.21	1.04	–	–	–	–	–	–	–	–	–	–	–	–	–	–
108	[(1*R*,4a*R*,4b*S*,7*R*,10a*R*)-7-ethenyl-1,4a,7-trimethyl-3,4,4b,5,6,9,10,10a-octahydro-2H-phenanthren-1-yl]methanol; [sandaracopimarinol] / df	$C_{21}H_{32}O_2$	2285	–	–	–	–	–	–	–	–	–	–	–	–	–	–	7.35	14.93	–	–	–	–	–	–
109	tricosane / h	$C_{23}H_{48}$	2300	13.47	61.14	10.84	17.28	2.22	2.84	10.79	12.77	2.59	191.96	4.24	4.92	13.73	13.94	7.83	6.87	1.11	11.86	1.39	2.76	1.04	0.58
110	[(3*S*,5*R*,8*R*,9*S*,10*S*,13*S*,14*S*,17*S*)-17-acetyl-10,13-dimethyl-2,3,4,5,6,7,8,9,11,12,14,15,16,17-tetradecahydro-1H-cyclopenta[a]phenanthren-3-yl] acetate / df	$C_{23}H_{36}O_3$	2309	–	–	–	–	–	–	–	–	–	–	–	–	–	–	–	–	10.19	53.29	–	–	–	–
111	1-[(5*R*,8*R*,9*S*,10*S*,13*R*,14*S*,17*S*)-13-(hydroxymethyl)-10-methyl-2,3,4,5,6,7,8,9,11,12,14,15,16,17-tetradecahydro-1H-cyclopenta[a]phenanthren-17-yl]ethanol; [pregnane-18,20-diol] / a	$C_{21}H_{36}O_2$	2316	–	–	–	–	–	–	1.64	4.42	–	–	–	–	–	–	–	–	–	–	–	–	–	–
112	methyl lambertianate / df	$C_{21}H_{30}O_3$	2319	–	–	–	–	–	–	–	–	–	–	–	–	–	–	1.41	2.86	–	–	–	–	–	–
113	(*E*)-8-(2,5,5,8a-tetramethyl-1,4,4a,6,7,8-hexahydronaphthalen-1-yl)-6-methyloct-5-en-2-ol / a	$C_{23}H_{40}O$	2326	–	–	–	–	–	–	8.99	7.10	–	–	–	–	–	–	–	–	–	–	–	–	–	–
114	methyl (1*S*,4a*R*,5*S*,8a*R*)-1,4a-dimethyl-6-methylene-5-[2-(2-oxo-2,5-dihydro-3-furanyl)ethyl]decahydro-1-naphthalenecarboxylate / e	$C_{21}H_{30}O_3$	2336	–	–	–	–	–	–	–	–	–	–	–	–	–	–	–	–	2.49	11.39	–	–	–	–
115	pentadecyl propan-2-yl sulfite / s	$C_{18}H_{38}O_3S$	2342	–	–	1.59	2.31	–	–	–	–	–	–	–	–	–	–	–	–	–	–	–	–	–	–
116	tetracosane / h	$C_{24}H_{50}$	2400	3.00	12.75	1.50	2.37	–	–	–	–	–	–	3.23	2.41	1.68	1.96	–	–	–	–	–	–	–	–
117	methyl (1*S*,4a*R*,5*S*,8a*R*)-5-[(*E*)-5-methoxy-3-methyl-5-oxopent-3-enyl]-1,4a-dimethyl-6-methylidene-3,4,5,7,8,8a-hexahydro-2H-naphthalene-1-carboxylate; [methyl athecate] / e	$C_{22}H_{34}O_4$	2402	–	–	–	–	–	–	–	–	–	–	–	–	–	–	–	–	11.21	90.48	–	–	–	–
118	methyl (1*S*,4a*R*,5*S*,8a*R*)-5-[2-(furan-3-yl)ethyl]-1,4a-dimethyl-6-methylidenedecahydronaphthalene-1-carboxylate; [methyl daniellate] / df	$C_{21}H_{30}O_3$	2409	–	–	–	–	–	–	–	–	–	–	–	–	–	–	2.10	4.26	–	–	–	–	–	–
119	1-[(3*S*,5*S*,8*S*,9*S*,10*S*,11*S*,13*S*,14*S*,17*S*)-3,11-dihydroxy-10,13-dimethyl-2,3,4,5,6,7,8,9,11,12,14,15,16,17-tetradecahydro-1H-cyclopenta[a]phenanthren-17-	$C_{21}H_{34}O_3$	2411	–	–	–	–	–	–	1.90	3.00	–	–	–	–	–	–	–	–	–	–	–	–	–	–

№	Substances / Chemical group	Formula	RI	*M. spicatum*		*S. × foliosum*		*P. amphibia*		*P. perfoliatus*		*N. lutea*		*S. gramineum*		*S. emersum*		*P. pectinatus*		*P. natans*		*L. dortmanna*		*C. demersum*	
				%	C	%	C	%	C	%	C	%	C	%	C	%	C	%	C	%	C	%	C	%	C
	yl]ethanone; [(3α,5β,11α)-3,11-dihydroxy-5β-pregnan-20-one] / df																								
120	(*Z*)-docos-13-en-1-ol; [Erucyl alcohol] / a	$C_{22}H_{44}O$	2434	–	–	–	–	–	–	–	–	–	–	–	–	–	–	–	–	–	–	1.34	2.66	–	–
121	(1*S*,4a*S*,5*R*)-5-[2-(furan-3-yl)ethyl]-1,4a-dimethyl-6-methylidene-3,4,5,7,8,8a-hexahydro-2H-naphthalene-1-carboxylic acid; [polyalthic acid] / df	$C_{20}H_{28}O_3$	2452	–	–	–	–	–	–	–	–	–	–	–	–	–	–	–	–	6.31	51.24	–	–	–	–
122	unidentified compound m/z ? [M+], 121 (100) / un	$C_{23}H_{40}O_3Si$	2463	–	–	–	–	–	–	–	–	–	–	–	–	–	–	–	–	4.58	20.00	–	–	–	–
123	[(8*R*,9*S*,10*R*,13*S*,14*S*)-4,10,13-trimethyl-3-oxo-1,2,6,7,8,9,11,12,14,15,16,17-dodecahydrocyclopenta[a]phenanthren-17-yl] acetate; (tentative library ID) / df	$C_{22}H_{32}O_3$	2467	–	–	–	–	–	–	5.20	1.12	–	–	–	–	–	–	–	–	1.42	15.16	–	–	–	–
124	(*Z*)-pentacos-12-ene / h	$C_{25}H_{50}$	2481	–	–	1.88	2.74	–	–	–	–	–	–	4.54	3.39	–	–	–	–	–	–	–	–	–	–
125	pentacosane / h	$C_{25}H_{52}$	2500	13.44	62.22	39.35	65.39	–	–	8.73	10.34	–	–	31.42	51.25	28.66	33.47	2.26	1.99	1.33	14.17	11.92	23.69	–	–
126	[(1*S*,2*R*,5*S*,7*R*,9*S*,11*S*,12*S*,15*S*,16*S*)-15-acetyl-2,16-dimethyl-8-oxapentacyclo[9.7.0.02,7.07,9.012,16]octadecan-5-yl] acetate; [(3β,5α,6α)-3-acetyloxy-5,6-epoxypregnan-20-one] / df	$C_{23}H_{34}O_4$	2544	–	–	–	–	–	–	3.66	5.17	–	–	–	–	–	–	–	–	–	–	–	–	–	–
127	methyl (1*R*,4*aR*,4*bS*,7*R*,10*aR*)-7-ethenyl-1,4*a*,7-trimethyl-3,4,4*b*,5,6,9,10,10*a*-octahydro-2H-phenanthrene-1-carboxylate; [methyl sandaracopimarate] / e	$C_{21}H_{32}O_2$	2555	–	–	–	–	–	–	–	–	–	–	–	–	–	–	–	–	10.56	112.48	–	–	–	–
128	(3*R*,5*R*,7*R*,8*R*,9*S*,10*S*,13*S*,14*S*,17*S*)-7,10,13,17-tetramethyl-1,2,3,4,5,6,7,8,9,11,12,14,15,16-tetradecahydrocyclopenta[a]phenanthrene-3,17-diol / df	$C_{21}H_{36}O_2$	2557	–	–	–	–	–	–	–	–	–	–	–	–	–	–	–	–	1.26	5.49	–	–	–	–
129	unidentified compound m/z 316 [M+], 121 (100) / un	–	2576	–	–	–	–	–	–	–	–	–	–	–	–	–	–	–	–	1.40	14.87	–	–	–	–
130	hexacosane / h	$C_{26}H_{54}$	2600	–	–	3.17	5.02	–	–	–	–	–	–	–	–	–	–	–	–	–	–	–	–	–	–
131	octadecyl propan-2-yl sulfite / s	$C_{21}H_{44}O_3S$	2680	–	–	2.19	3.18	–	–	–	–	–	–	10.37	17.34	–	–	–	–	–	–	–	–	–	–
132	pentacosan-1-ol / a	$C_{25}H_{52}O$	2685	–	–	–	–	–	–	2.27	2.68	–	–	–	–	–	–	–	–	–	–	–	–	–	–
133	heptacosane / h	$C_{27}H_{56}$	2700	2.79	16.39	17.39	27.49	–	–	2.16	2.55	–	–	7.56	10.08	10.95	12.78	–	–	–	–	–	–	–	–
134	(3-acetyloxy-10,13-dimethyl-1-oxo-2,3,4,5,6,7,8,9,11,12,14,15,16,17-tetradecahydrocyclopenta[a]phenanthren-17-yl) acetate; [(5β)3,17-Diacetoxyandrostan-1-one] / df	$C_{23}H_{34}O_5$	2711	–	–	–	–	–	–	1.06	2.84	–	–	–	–	–	–	–	–	–	–	–	–	–	–
135	[(3*S*,5*S*,8*R*,9*S*,10*S*,13*R*,17*R*)-17-[(*E*,2*R*,5*S*)-5,6-dimethylhept-3-en-2-yl]-10,13-dimethyl-2,3,4,5,6,7,8,9,11,12,16,17-dodecahydro-1H-cyclopenta[a]phenanthren-3-yl] acetate; [ergosta-14,22-dien-3-ol, acetate, (3β,5α,22E)] / df	$C_{30}H_{50}O_2$	2712	–	–	–	–	–	–	2.78	1.18	–	–	–	–	–	–	–	–	2.42	25.79	–	–	–	–
136	(6*E*,10*E*,14*E*,18*E*)-2,6,10,15,19,23-hexamethyltetracosa-2,6,10,14,18,22-hexaene; [squalene] / h	$C_{30}H_{50}$	2822	–	–	–	–	–	–	2.06	0.62	–	–	–	–	–	–	–	–	–	–	–	–	–	–
137	hexacosan-1-ol / a	$C_{26}H_{54}O$	2878	–	–	–	–	–	–	2.28	2.70	–	–	–	–	–	–	–	–	–	–	–	–	–	–

Note: for some compounds, trivial or most frequently used names are given in square brackets; "–" – the component is absent as a major; a – alcohols; ald – aldehydes; h – hydrocarbons; fa – fatty acids; e – esters; k – ketones; df – diverse functional groups; p – phenols; ah – aromatic hydrocarbons; s – sulfur-containing; un – unidentified compounds.

Availability of raw GC–MS data and identification spectra. Raw GC–MS data (instrument raw files) and compound identification spectra/outputs are available from the corresponding author upon reasonable request.
